# The Effect of Non‐Surgical Periodontal Therapy on Subgingival Microbiota: A Systematic Review and Meta‐Analysis

**DOI:** 10.1111/jre.13409

**Published:** 2025-05-09

**Authors:** Anna Krajewski, Jeniffer Perussolo, Pinar Ercal, Nikolaos Gkranias, Nikos Donos

**Affiliations:** ^1^ Centre for Oral Clinical Research, Institute of Dentistry, Faculty of Medicine and Dentistry Queen Mary University of London London UK

**Keywords:** meta‐analysis, non‐surgical periodontal therapy, periodontitis, subgingival microbiota, systematic review

## Abstract

**Aim:**

The objective of this systematic review and meta‐analysis was to evaluate the influence of non‐surgical periodontal therapy (NSPT) on subgingival microbiota in systemically healthy patients with periodontitis.

**Methods:**

A systematic literature search was conducted in Medline, Embase, LILACS, and the Cochrane Library, supplemented by a manual search for studies investigating the effect of NSPT on subgingival microbiota in systemically healthy patients.

**Results:**

In this review, 115 studies were included, of which 41 used checkerboard DNA–DNA hybridisation technology, 30 real‐time quantitative PCR, 36 bacterial culture, and 8 16S ribosomal ribonucleic acid (rRNA) gene sequencing technology. Meta‐analyses showed a significant decrease in mean counts of *Aggregatibacter actinomycetemcomitans* ( *A. actinomycetemcomitans* ), *Treponema denticola* ( *T. denticola* ), *Porphyromonas gingivalis* ( *P. gingivalis* ), *Tannerella forsythia* ( *T. forsythia* ), *Eubacterium nodatum* ( *E. nodatum* ), *Prevotella intermedia* ( *P. intermedia* ), and *Campylobacter rectus* ( *C. rectus* ) after NSPT. Supportive periodontal care provided a further decrease in mean counts of *P. gingivalis* and *T. denticola* at 6 months after NSPT. Qualitative analysis of 16S rRNA gene sequencing studies indicated significant effects of NSPT on the relative abundance of selected disease‐and health‐associated species and genera, whereas findings on changes in alpha and beta diversity in the subgingival microbiome were heterogeneous.

**Conclusion:**

This systematic review showed that NSPT leads to significant reduction of mean abundance of disease‐associated microbial species. However, 16S rRNA gene sequencing studies reported heterogeneous findings, which may be associated with methodological aspects. To deepen the understanding of the effect of NSPT on subgingival microbiota, further studies, powered for microbiome changes and with clustered analysis of site‐specific samples, are needed.

## Graphical abstract

This paper provides crucial insights and evidence on the effect of non‐surgical periodontal therapy on subgingival microbiota. NSPT leads to significant reductions in disease‐associated microbial species. Supportive periodontal care provision provided further decrease in mean counts of disease‐associated species. Study methods may influence microbiological findings. Therefore, microbiome studies should perform sample size calculation on the basis of microbiological outcomes, collect site‐specific samples, and analyse data according to clinical outcomes from sampling sites.

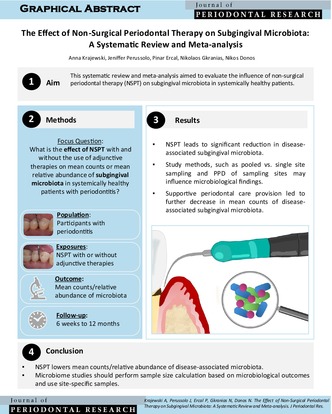

## 1 Introduction

Periodontal inflammation is driven by changes in the subgingival microbiome [1]. In a process called dysbiosis, the loss or reduction of potentially beneficial microorganisms in subgingival microbiota is accompanied by an increase of disease‐associated species [2]. The dysbiotic microbiome can disturb the balance of immune and inflammatory responses of the host [3] and periodontal tissue turnover, leading to tissue destruction and bone resorption [4].

Subgingival microorganisms and their influence on the health‐disease process have been widely studied with evolving technologies, such as microscope [5], anaerobic bacterial culture [6], polymerase chain reaction (PCR), [7] and checkerboard DNA–DNA hybridisation technology [8]. Certain bacterial species, such as the three red complex bacteria *Porphyromonas gingivalis* ( *P. gingivalis* ), *Treponema denticola* ( *T. denticola* ) and *Tannerella forsythia* ( *T. forsythia* ) or *Aggregatibacter actinomycetemcomitans* ( *A. actinomycetemcomitans* ) have been shown to be associated with the development and progression of periodontitis. An increase in the prevalence of red complex bacteria has been strongly associated with diseased sites in periodontitis [8, 9, 10]. *P. gingivalis* has been labelled a keystone pathogen, due to its ability to alter the innate immunity, which enables destructive changes in the host–microbial interplay [9]. More recently, next‐generation sequencing technologies have been used to analyse subgingival microbial communities [2, 11]. These novel and advanced technologies have shown that periodontitis dysbiosis is the result of wide community changes in a complex subgingival microbiome composed of hundreds of bacterial species and is not caused only by disease‐associated bacteria [1].

The European Federation of Periodontology (EFP) has recently published a S‐3 level clinical guideline, recommending a stepwise therapeutic pathway/approach for the management of periodontitis (EFP S3) [12, 13]. Non‐surgical periodontal therapy (NSPT) consists of Step 1, which addresses the patient's oral hygiene and the control of modifiable risk factors, such as smoking cessation [14] and/or blood sugar control [15, 16, 17, 18] and Step 2, which aims to thoroughly remove subgingival plaque. During this phase of periodontal therapy, adjunctive antimicrobial therapies may be considered [19, 20]. In cases with deep residual pockets, additional NSPT and/or periodontal surgery (Step 3) may be suggested [13, 21]. The main goal of this stepwise approach is to achieve the endpoints of periodontal therapy, which are no periodontal pockets > 4 mm with bleeding on probing or no deep periodontal pockets with PPD ≥ 6 mm [22, 23, 24], because these conditions have been shown to be associated with the long‐term stability of periodontal tissues [25].

NSPT has been shown to affect subgingival microbiota. A sharp three‐ to fourfold decrease in mean counts of viable cultivable gram‐negative anaerobic species was demonstrated after NSPT [26]. The changes occur in all periodontal sites independent of the initial probing pocket depth [27], but sites with a decrease in PPD of over 2 mm were more likely to experience significant reductions in mean counts of subgingival bacteria at 12 months after therapy [27]. Previously, it was demonstrated that greater initial mean counts of *P. gingivalis* are correlated to greater pocket depth reductions following NSPT [27]. Furthermore, total bacterial load as well as counts of red complex bacteria at baseline are both negatively associated with clinical attachment level (CAL) gains 1 year following treatment [28]. More recently, it has become of interest to understand how periodontal health‐associated microbiota may assist with long‐term periodontal stability after NSPT [29, 30].

Within the limits of the different microbiological technologies used, the current literature supports the evidence of a reduction in the total counts of subgingival bacteria, a decrease in the proportion of disease‐associated species and an increase in host‐compatible species. However, to the best of our knowledge, there is currently no systematic review that comprehensively evaluates and synthesises the results of a wide range of articles and microbiological detection technologies, while also assessing the quality of the studies. For the first time, this systematic review and meta‐analysis aims to evaluate the extensive literature on the effects of NSPT on subgingival microbiota in systemically healthy patients.

## 2 Methods

The study protocol was registered with the International Register of Systematic Reviews, PROSPERO (CRD42020167170; http://www.crd.york.ac.uk/PROSPERO) and was prepared in line with the Cochrane Handbook [31]. The instructions of the Preferred Reporting Items for Systematic Review and Meta‐analysis (PRISMA) were adopted.

### 2.1 Focused Question

The present systematic review addressed the following focused question:

- What is the effect of NSPT, with and without the use of adjunctive therapies (apart from antibiotics), on mean counts or mean relative abundance of subgingival microbiota in systemically healthy patients with periodontitis?

### 2.2 Eligibility Criteria

The following inclusion criteria (based on the PICOS) were considered:

Population: systemically healthy adults (≥ 18 years old) diagnosed with periodontitis.

Intervention: NSPT, including studies with and without the use of adjunctive therapy.

Comparison: mean counts/or mean relative abundance of subgingival microbiota before and after NSPT. The selection was limited to studies with a minimum follow‐up of 6 weeks after NSPT.

Outcomes:

*Primary outcome*: changes to mean counts or mean abundance of subgingival microbiota after treatment.

*Secondary outcomes*: changes to clinical parameters, such as periodontal probing depth (PPD), clinical attachment loss (CAL), bleeding on probing (BoP) and full‐mouth plaque scores (FMPS).

Study design: randomised controlled trials (RCTs), controlled trials (CT), prospective single‐arm clinical trials, longitudinal (long.) studies and case–control studies (CCS). Split‐mouth studies were also included.

#### 2.2.1 Exclusion Criteria

Studies that included patients with systemic diseases and pregnant or lactating women or which did not provide clinical data were excluded. In addition, patients who had received NSPT prior to the study and patients who had received local or systemic antibiotic therapy up to 3 months before the onset or during the study, or as part of the NSPT treatment protocol, were also excluded. Data from control groups or control study arms with healthy patients and no antibiotic use were included. Smoking was not an exclusion criterion.

### 2.3 Search Strategy and Data Management

The literature search was conducted on Medline (via OVID), EMBASE, LILACS, and Cochrane databases on 23 December 2019 and updated on 1 June 2024. The search strategy included Mesh terms and free text terms related to the Population, Intervention and Comparison investigated in this review, connected with the Boolean operator ‘AND’. Any study published in English, German, Spanish, Greek, or Portuguese was considered. Literature search results were uploaded to the Covidence platform (https://www.covidence.org/), which automatically deleted all duplicates.

### 2.4 Study Selection

Three independent reviewers (A.K., P.E. and J.P.) carried out a three‐stage screening. Prior to the formal screening process, a calibration exercise was undertaken to pilot and refine the screening questions. The first‐stage screening of titles and abstracts was carried out to eliminate studies which did not meet the inclusion criteria (A.K. and J.P.). At the second stage, screening all studies referring to NSPT were selected for full text screening (A.K. and J.P.). Whenever the full text study was not available, authors were contacted. Following proofreading of the full text, studies eligibility was verified independently by reviewers as a third step (A.K. and P.E.). Disagreements were resolved by consensus; if necessary, a fourth reviewer (N.G.) was consulted. The level of agreement between the reviewers was calculated using Cohen's Kappa statistics.

### 2.5 Data Collection Process

Two reviewers (A.K. and P.E.) performed data extraction in duplicates. Due to the high volume of studies identified, data extraction in two stages was implemented. First, general study characteristics (authors, year of publication, country and setting), study design, smoking status, and microbiological detection technique were extracted from all included studies. Considering that a meta‐analysis was to be performed, microbiological and clinical data were only extracted from checkerboard DNA–DNA hybridisation technology studies, real‐time quantitative PCR studies, bacterial culture studies, and 16S rRNA gene sequencing studies.

Whenever data were not available or additional information was required, the authors were contacted to retrieve missing data, such as standard deviations (SD) or standard errors (SE) from mean values. Data presented in figures/graphs, where numerical values were not available, were extracted using the validated [32, 33, 34] online tool WebPlotDigitizer (https://automeris.io/WebPlotDigitizer/), in line with the Cochrane Handbook recommendations (https://training.cochrane.org/handbook/current—Chapter 5.5.8: Extracting data from figures with software). To ensure high accuracy in data extraction from figures/graphs, the reviewers conducted a calibration exercise. The intraclass correlation coefficient (ICC) across different measurements exceeded 0.9, demonstrating excellent reliability between authors.

### 2.6 Quality Assessment and Risk of Bias

Two reviewers (A.K. and P.E.) assessed the methodological quality of checkerboard DNA–DNA hybridisation technology, real‐time quantitative PCR, bacterial culture, and 16S rRNA gene sequencing studies. For RCTs, the Cochrane Risk of Bias 2 tool (RoB2, updated on 22 August 2019, https://sites.google.com/site/riskofbiastool/welcome/rob‐2‐0‐tool/current‐version‐of‐rob‐2) was used. The tool assesses the risk of bias across five domains: randomisation process, deviation from intended interventions, missing outcome data, measurement of the outcome and selection of the reported results.

The remaining studies were assessed with the Risk of Bias in Non‐Randomized Studies of Interventions tool (ROBINS‐I, sites.google.com/site/riskofbiastool/welcome/home/current‐version‐of‐robins‐i). The tool assesses risk of bias across seven domains: confounding, classification of interventions, selection of participants, deviation from intended interventions, missing data, measurement of the outcome and selection of reported results. Each study was judged as at low, moderate, high or unclear risk of bias. Additional information on the statistical power of the study and sample size calculation was also assessed and recorded.

### 2.7 Data Synthesis and Meta‐Analyses

Meta‐analysis was performed when ≥ 5 studies reported on species with the same microbiological detection technique and the same follow‐up time‐point. Checkerboard DNA–DNA hybridisation technology, real‐time quantitative PCR and bacterial culture studies were used for meta‐analysis. There were not an adequate number of 16S rRNA gene sequencing studies to perform a meta‐analysis. Studies were clustered into two distinct groups: *Group 1* included only studies that did NOT use any adjunctive therapies and *Group 2* included studies that used adjunctive therapies as part of the NSPT protocol.

For studies presenting *p* values but neither SD nor SE, the SD was estimated by reversing *p* value calculations. Similarly, SD was calculated if confidence intervals (CI) were reported. Subgingival microbiota changes following NSPT were calculated with a longitudinal random‐effects model (DerSimonian‐Laird) implemented by using software package Stata (version 16.1). The effect size was measured as Hedges' g. A funnel plot was used to evaluate publication bias within the meta‐analysis.

A complementary sensitivity analysis was performed on a subset of studies restricting the use of CHX to long‐term use (6 to 8 weeks) as a twice daily mouthwash to assess a more homogenous subgroup of patients.

## 3 Results

### 3.1 Study Selection

Initial data search retrieved a total of 3067 studies. After removal of duplicates, studies were screened for eligibility. Following first and second stage screening, 517 studies qualified for full text screening. Of those, 196 studies met the inclusion and exclusion criteria. This included checkerboard DNA–DNA hybridisation technology studies (*n* = 41), real‐time quantitative PCR technology studies (*n* = 30), bacterial culture technology studies (*n* = 36), and 16S rRNA gene sequencing technology studies (*n* = 8) (Appendix S1). Kappa scores were calculated for the level of agreement for title/abstract and full text screening (kappa: 0.68 and 0.77, respectively) showing a substantial agreement between the three reviewers. The specific reasons for exclusion are listed in Appendix S2.

The included studies were published in a timespan of over 30 years, from 1983 [35] to 2024 [36]. The studies were conducted in 31 different countries. Most studies were carried out in Brazil (22%), followed by Germany (10%), India (9%), United States (8%), Turkey (7%), United Kingdom (5%), Italy (5%), Netherlands (4%), Belgium, Sweden (4%) and other countries (23%).

Most studies were RCTs (80%), followed by longitudinal studies (11%), CSS (7%) and CTs (2%). One hundred thirty‐five studies were based on a whole‐mouth design (70%), and 57 studies applied a split‐mouth design (30%). Eighty‐four studies (43%) included only non‐smoking subjects, 54 studies (28%) included both smoking and non‐smoking subjects, seven studies (4%) included smoking and non‐smoking subjects in a case–control design, six studies (3%) included only smoking subjects, and 43 studies (22%) did not report the smoking status of the study subjects (Figure 2A–C).

Six different microbiological analysis technologies were used in the included studies: PCR (4%), of which 30 used quantitative PCR (real‐time quantitative PCR, 15%), checkerboard DNA–DNA hybridisation technology (21%), bacterial culture (19%), dark field microscope (12%), 16S rRNA gene sequencing (4%), and other technologies (4%). Some studies used more than one microbiological technique. Microbiological outcomes were reported as either positive (bacterium detected) or negative (bacterium not detected) per case/per site or as mean values and changes over time. Supporting Information includes a figure illustrating the main characteristics of the included studies (Appendix S3A–D), a table providing an overview of study characteristics of all identified studies (Appendix S4) and the reference list of all identified studies (Appendix S5).

### 3.2 Results From Studies Employing Checkerboard DNA–DNA Hybridisation Technology

#### 3.2.1 Study Characteristics and Treatment Modalities

Forty‐one studies used checkerboard DNA–DNA hybridisation technology as the microbiological detection technique [37, 38, 39, 40, 41, 42, 43, 44, 45, 46, 47, 48, 49, 50, 51, 52, 53, 54, 55, 56, 57, 58, 59, 60, 61, 62, 63, 64, 65, 66, 67, 68, 69, 70, 71, 72, 73, 74, 75, 76, 77]. Thirty‐two studies (78%) were RCTs [37, 38, 41, 42, 43, 46, 47, 51, 52, 53, 54, 55, 56, 57, 58, 59, 60, 61, 62, 63, 64, 65, 67, 68, 69, 70, 71, 73, 74, 75, 76, 77]; four studies (10%) were CSS, [39, 47, 66, 72] and five studies (12%) were prospective longitudinal single‐arm studies (long.) [40, 44, 45, 49, 50]. Ten of the RCTs (32%) used a split‐mouth study design [42, 43, 46, 55, 57, 60, 63, 64, 69, 70] to compare the different treatment modalities, whereas the other 21 RCTs (68%) used whole‐mouth study designs [37, 38, 41, 48, 51, 53, 54, 56, 59, 61, 62, 65, 67, 68, 71, 73, 74, 75, 76, 77].

Based on the 1999 WWP classification [78], 34 studies (80%) included patients with a diagnosis of chronic periodontitis [37, 39, 40, 41, 42, 43, 44, 45, 46, 47, 48, 49, 50, 51, 53, 54, 55, 56, 57, 58, 59, 61, 65, 66, 67, 68, 69, 70, 71, 72, 74, 75, 76, 77, 79], seven studies (17%) included patients with aggressive periodontitis [38, 52, 60, 62, 63, 64, 73, 80] and one study (2%) included patients with both diagnoses [66]. The sample size ranged from 6 [56] to 57 subjects [49, 50].

All studies performed NSPT and assessed subgingival microbiota before and after treatment. Various adjunctive therapies were applied in addition to mechanical NSPT, including: CHX (17%) [40, 42, 43, 48, 52, 62, 74], photodynamic therapy (PDT) (12%) [38, 46, 63, 64, 75], povidone‐iodine (PVP) (5%) [57, 65], desiccant (2%) [55], diode laser (2%), [60] and controlled local delivery of chlorhexidine chips (2%) [67].

The most common time point to measure subgingival microbiota after NSPT was at 3 months, which was selected in 32 studies (78%) [37, 38, 40, 41, 44, 46, 48, 49, 50, 51, 52, 53, 54, 56, 57, 58, 59, 61, 62, 63, 64, 65, 66, 67, 69, 70, 71, 72, 73, 74, 75, 76]. This was followed by 6 months in 26 studies (63%) [37, 39, 40, 42, 43, 44, 48, 49, 51, 52, 53, 54, 55, 56, 57, 58, 60, 61, 67, 68, 70, 73, 74, 75, 76, 77], 12 months in 11 studies (27%) [45, 55, 58, 59, 61, 62, 68, 70, 73, 74, 75, 76], 9 months in six studies (14%) [44, 48, 49, 58, 61, 76], 1.5 months in five studies (10%), [40, 55, 60, 75, 77] and 24 months in one study (2%) [61]. Table 1 provides information about the study characteristics and treatment modalities of the checkerboard DNA–DNA hybridisation technology studies.

**TABLE 1:** Study characteristics and treatment modalities of checkerboard DNA–DNA hybridisation technology studies (*n* = 41), real‐time quantitative PCR studies (*n* = 30), microbial culture studies (*n* = 36), and 16S rRNA gene sequencing studies (*n* = 8).

Authors (year)	Country	Study design	Split‐ mouth	Sample size	Diagnosis	Smoking status	Treatment specifics	Adjunctive therapy	Follow‐up mths
**Checkerboard DNA–DNA hybridisation technology studies**									
Apatzidou et al. (2014) [37]	Greece	RCT	No	22	Chronic PD	Both	NSPT w or w/o weekly PC		3 & 6
Borekci et al. (2019) [38]	Turkey	RCT	No	12	Aggr. PD	Non‐smoker	NSPT w or w/o PDT	PDT	3
Carvalho et al. (2005) [41]	Brazil	RCT	No	10	Chronic PD	N/I	NSPT w or w/o weekly PC		3
Christgau et al. (2006) [42]	Germany	RCT	Yes	20	Chronic PD	Both	NSPT, hand vs. ultrasonic scalers	CHX	6
Christgau et al. (2007) [43]	Germany	RCT	Yes	20	Chronic PD	Both	NSPT, hand vs. ultrasonic scalers	CHX	6
De Melo Soares et al. (2019) [46]	Brazil	RCT	Yes	20	Chronic PD	Smoker	NSPT w or w/o PDT	PDT	3
Feres et al. (2009) [48]	Brazil	RCT	No	20	Chronic PD	Non‐smoker	NSPT w or w/o weekly PC or CHX	CHX	2 & 6
Haffajee et al. (2008) [51]	USA	RCT	No	43	Chronic PD	Both	NSPT w or w/o Periostat	Periostat	3 & 6
Heller et al. (2011) [52]	Brazil	RCT	No	15	Aggr. PD	Both	NSPT w or w/o AB	CHX	3 & 6
Ioannou et al. (2009) [53]	Greece	RCT	No	40	Chronic PD	Both	NSPT, hand vs. ultrasonic scalers		3 & 6
Ioannou et al. (2011) [54]	Greece	RCT	No	16	Chronic PD	Both	NSPT w or w/o loc. AB		3 & 6
Isola et al. (2018) [55]	Italy	RCT	Yes	36	Chronic PD	Non‐smoker	NSPT w or w/o desiccant	Desiccant	1.5, 6 & 12
Jones et al. (1994) [56]	USA	RCT	No	6	Chronic PD	N/I	NSPT w or w/o AB		3 & 6
Leonhardt et al. (2007) [57]	Sweden	RCT	Yes	20	Chronic PD	Non‐smoker	NSPT w or w/o PVP	PVP	3 & 6
Lopez et al. (2006) [58]	Chile	RCT	No	11	Chronic PD	Both	NSPT w or w/o AB		3, 6, 9 & 12
Matarazzo et al. (2008) [59]	Brazil	RCT	No	15	Chronic PD	Smoker	NSPT w or w/o AB		3
Matarese et al. (2017) [60]	Italy	RCT	Yes	31	Aggr. PD	Non‐smoker	NSPT w or w/o Laser	Laser	1.5, 6 & 12
Mdala et al. (2013) [61]	Norway/USA	RCT	No	26	Chronic PD	Both	NSPT w or w/o AB		3, 6, 9, 12 & 24
Mestnik et al. (2010) [62]	Brazil	RCT	No	15	Aggr. PD	Non‐smoker	NSPT w or w/o AB	CHX	3
Moreira et al. (2015) [63]	Brazil	RCT	Yes	20	Aggr. PD	Non‐smoker	NSPT w or w/o PDT	PDT	3
Novaes et al. (2012) [64]	Brazil	RCT	Yes	10	Aggr. PD	Non‐smoker	NSPT w or w/o PDT	PDT	3
Perrella et al. (2016) [65]	Brazil	RCT	No	29	Chronic PD	Non‐smoker	NSPT w or w/o PVP	PVP	3
Sakellari et al. (2010) [67]	Greece	RCT	No	25	Chronic PD	Both	NSPT w or w/o PerioChip	PerioChip	3 & 6
Sampaio et al. (2011) [68]	Brazil	RCT	No	20	Chronic PD	Both	NSPT w or w/o AB		6 & 12
Shiloa et al. (1997) [69]	USA	RCT	Yes	12	Chronic PD	Both	NSPT vs. surgery		3
Shiloa et al. (1998) [70]	USA	RCT	Yes	10	Chronic PD	Both	NSPT vs. surgery		3, 6 & 12
Silva et al. (2011) [71]	Brazil	RCT	No	17	Chronic PD	Non‐smoker	NSPT w or w/o AB		3
Silva‐Senem et al. (2013) [73]	Brazil	RCT	No	17	Aggr. PD	Both	NSPT w or w/o AB		3, 6 & 12
Soares et al. (2014) [74]	Brazil	RCT	No	40	Chronic PD	Non‐smoker	NSPT w or w/o AB	CHX	3, 6 & 12
Tabenski et al. (2017) [75]	Germany	RCT	No	15	Chronic PD	Both	NSPT w or w/o PDT	PDT	1.5, 3, 6 & 12
Timmerman et al. (1996) [76]	Netherlands	RCT	No	20	Chronic PD	N/I	NSPT w or w/o AB		3, 6, 9 & 12
Xajigeorgiou et al. (2006) [77]	Greece	RCT	No	37	Chronic PD	Both	NSPT w or w/o AB		1.5 & 6
Brochut et al. (2005) [40]	Switzerland	Long	No	10	Chronic PD	Non‐smoker	NSPT	CHX	1.5, 3 & 6
Colombo et al. (2005) [44]	Brazil	Long	No	25	Chronic PD	Both	NSPT		3, 6 & 9
Cugini et al. (2000) [45]	USA	Long	No	57	Chronic PD	Both	NSPT		12
Haffajee et al. (1997) [49]	USA	Long	No	57	Chronic PD	Both	NSPT		3, 6 & 9
Haffajee et al. (1997) [50]	USA	Long	No	57	Chronic PD	Both	NSPT		3
Bozoglan et al. (2017) [39]	Turkey	CCS	No	20	Chronic PD	Non‐smoker	NSPT RA vs. healthy		6
Feres et al. (2015) [47]	Brazil	CCS	No	15	Chronic PD	Both, Case–Control	NSPT, smokers vs. non‐smokers		2, 3 & 7
Rosalem et al. (2011) [66]	Brazil	CCS	No	34	Both	Both	NSPT chronic vs. aggr.		3
Silva‐Boghossian et al. (2014) [72]	Brazil	CCS	No	20	Chronic PD	Non‐smoker	NSPT healthy vs. diabetic		3
**Real‐time quantitative PCR studies**									
Bergamaschi et al. (2016) [81]	Brazil	RCT	No	10	Chronic PD	Smoker	NSPT w or w/o AB		3
Chitsazi et al. (2014) [82]	Iran	RCT	Yes	24	Aggr. PD	Non‐smoker	NSPT w or w/o diode laser	Laser	3
Cirino et al. (2019) [79]	Brazil	RCT	Yes	16	Aggr. PD	Non‐smoker	NSPT vs. surgery		3 & 12
Cortelli et al. (2015) [83]	Brazil	RCT	No	100	Chronic PD	Both	NSPT w or w/o CHX	CHX	3 & 6
Del Peloso Ribeiro et al. (2008) [84]	Brazil	RCT	No	25	Chronic PD	Non‐smoker	NSPT vs. FMD		3
Do Vale et al. (2016) [85]	Brazil	RCT	No	28	Aggr. PD	Non‐smoker	NSPT w or w/o PVP	PVP	1.5 & 3
Eick et al. (2013) [86]	Germany	RCT	No	18	Chronic PD	Non‐smoker	NSPT w or w/o hyaluronic acid	Hyaluronic acid	3 & 6
Fonseca et al. (2015) [87]	Brazil	RCT	No	85	Chronic PD	Both	NSPT w or w/o CHX	CHX	3 & 6
Grzech‐Lesniak et al. (2018) [88]	Poland	RCT	No	84	Chronic PD	Non‐smoker	NSPT w or w/o diode laser	Laser	3 & 6
Guentsch et al. 2008 [89]	Germany	RCT	No	21	Chronic PD	Both	NSPT w or w/o AB		3, 6 & 12
Han et al. (2012) [90]	Turkey	RCT	No	14	Chronic PD	Both	NSPT w or w/o AB		1.5 & 6
Hayakumo et al. (2013) [91]	Japan	RCT	No	21	Chronic PD	Non‐smoker	NSPT w or w/o ozone	Ozone	1 & 2
Jervoe‐Storm et al. (2007) [92]	Germany	RCT	No	20	Chronic PD	Both	NSPT vs. FMD		6
Luchesi et al. (2013) [93]	Brazil	RCT	No	37	Chronic PD	Non‐smoker	NSPT w or w/o PDT	PDT	3 & 6
Park et al. (2018) [94]	Korea	RCT	Yes	21	Chronic PD	Both	NSPT w or w/o erythritol powder	Erythritol powder	3
Pulikkotil et al. (2016) [95]	Malaysia	RCT	Yes	20	Chronic PD	Non‐smoker	NSPT w or w/o PDT	PDT	3
Ramiro et al. (2018) [96]	Brazil	RCT	No	59	Chronic PD	Non‐smoker	NSPT w or w/o AB		
Saglam et al. (2013) [97]	Turkey	RCT	No	45	Chronic PD	Non‐smoker	NSPT w or w/o CHX or boric acid	CHX or boric acid	1.5 & 3
Saglam et al. (2017) [98]	Turkey	RCT	YEs	25	Chronic PD	Non‐smoker	NSPT w or w/o laser	Laser	3
Soeroso et al. (2017) [99]	Indonesia	RCT	No	39	Chronic PD	Non‐smoker	NSPT w or w/o AB		3 & 6
Swierkot et al. (2009) [100]	Germany	RCT	No	25	Chronic PD	Both	NSPT w or w/o CHX	CHX	1.5
Teughels et al. (2013) [101]	Turkey	RCT	No	30	Chronic PD	Non‐smoker	NSPT w or w/o probiotics	Probiotics	1.5 & 3
Theodoro et al. 2018 [102]	Brazil	RCT	No	51	Chronic PD	Smoker	NSPT w or w/o PDT	PDT	3 & 6
Zengin Celik et al. (2019) [103]	Turkey	RCT	No	38	Chronic PD	Non‐smoker	NSPY w or w/o laser	Laser	3 & 6
Spooner et al. (2016) [104]	USA	Long.	No	15	Chronic PD	Both	NSPT		Not given
Buzinin et al. (2014) [105]	Malaysia	CCS	No	30	Chronic PD	Both	NSPT diabetic vs. healthy		3
Cosgarea et al. (2019) [106]	Romania	CCS	No	36	Chronic PD	Both	NSPT health vs. RA	CHX	3 & 6
Liu et al. (2013) [107]	China	CCS	No	130	Both	Non‐smoker	NSPT chronic vs. aggr.		3
Peralta et al. (2020) [108]	Brazil	CCS	No	94	Chronic PD	Both	Non‐obese vs. obese	CHX	3, 6 & 9
Tanaka et al. (2015) [101]	Brazil	CCS	No	35	Chronic PD	Non‐smoker	Healthy vs. Down syndrome		1.5
**Bacterial culture studies**									
Annaji et al. (2016) [109]	India	RCT	1	15	Aggr. PD	Non‐Smoker	NSPT w. or w/o laser or PDT	Laser, PDT	1 & 3
Ardila et al. (2015) [110]	Colombia	RCT	0	36	Aggr. PD	Non‐Smoker	NSPT w. or w/o AB		3 & 6
Ardila et al. (2017) [111]	Colombia	RCT	0	36	Aggr. PD	Non‐Smoker	NSPT w. or w/o AB		6
Berglundh et al. (1998) [112]	Sweden	RCT	0	16	Chronic PD	N/I	NSPT w. or w/o AB		2, 12 & 24
Bhatia et al. (2014) [113]	India	RCT	1	25	Chronic PD	Non‐Smoker	NSPT w. or w/o curcuma gel	Curcuma gel	1, 3 & 6
Bizzarro et al. (2016) [109]	Netherlands	RCT	0	53	Chronic PD	Both	NSPT w. or w/o NaCl	NaCl	3, 6 & 12
De Micheli et al. (2011) [114]	Brazil	RCT	1	28	Chronic PD	Non‐Smoker	NSPT w. or w/o laser	Laser	1.5
De Soete et al. (2005) [115]	Belgium	RCT	0	71	Chronic PD	Both	NSPT vs. FMD (w. or w/o CHX)	CHX (test group)	2, 4 & 8
Dhaliwal et al. (2017) [116]	India	RCT	0	30	Chronic PD	N/I	NSPT w. or w/o probiotic	Probiotic	1 & 3
Dominguez et al. (2010) [117]	Spain	RCT	0	30	Chronic PD	Non‐Smoker	NSPT w. or w/o laser	Laser	1 & 2
Euzebio Alves et al. (2013) [118]	Brazil	RCT	1	36	Chronic PD	Non‐Smoker	NSPT w. or w/o laser	Laser	1.5 & 6
Gibson et al. (1994) [119]	UK	RCT	1	24	N/I	N/I	NSPT w. or w/o methylene blue gel	Methylene blue gel	1, 2 & 3
Gomez et al. (2011) [120]	Spain	RCT	0	30	Chronic PD	Non‐Smoker	NSPT w. or w/o laser	Laser	1 & 2
Lombardo et al. (2015) [121]	Italy	RCT	1	16	Chronic PD	Non‐Smoker	NSPT w. or w/o desiccant	Desiccant	3
Manikandan et al. (2016) [122]	India	RCT	1	11	Chronic PD	N/I	NSPT w. or w/o CHX varnish	CHX varnish	1 & 2
Martande et al. (2016) [123]	India	RCT	0	70	Chronic PD	Non‐Smoker	NSPT w. or w/o AB		3, 6 & 12
Moeintaghavi et al. (2007) [124]	Iran	RCT	0	50	Chronic PD	Non‐Smoker	NSPT w. or w/o AB		2
Morales et al. (2018) [125]	Chile	RCT	0	31	Chronic PD	Both	NSPT w. or w/o probiotic	Probiotic	3, 6 & 9
Pradeep et al. (2014) [126]	India	RCT	0	69	Chronic PD	N/I	NSPT w. or w/o AB		3 & 6
Quirynen et al. (1995) [127]	Belgium	RCT	0	10	Chronic PD	Both	NSPT vs. FMD (w. CHX)	CHX	1 & 2
Quirynen et al. (1999) [128]	Belgium	RCT	0	40	Chronic PD	Both	NSPT vs. FMD (w. CHX)	CHX	1, 2, 4 & 8
Quirynen et al. (2000) [129]	Belgium	RCT	0	36	Chronic PD	Both	NSPT vs. FMD (w. or w/o CHX)	CHX	1, 2, 4 & 8
Roman‐Torres et al. (2018) [130]	Brazil	RCT	0	230	Chronic PD	Non‐Smoker	NSPT vs. FMD (w. CHX)	CHX	2
Rooney et al. (2002) [131]	UK	RCT	0	66	Chronic PD	N/I	NSPT w. or w/o AB		1, 3 & 6
Sanz‐Sanchez et al. (2016) [132]	Spain	RCT	0	40	Chronic PD	Both	NSPT w. or w/o laser	Laser	3 & 12
Sefton et al. (1996) [133]	UK	RCT	0	46	Chronic PD	N/I	NSPT w. or w/o AB		1.5, 2.5 & 6
Sreedhar et al. (2015) [134]	India	RCT	1	15	Chronic PD	Non‐Smoker	NSPT w. or w/o curcuma gel	Curcuma gel	3
Suchetha et al. (2013) [135]	India	RCT	0	75	Chronic PD	Non‐Smoker	NSPT w. or w/o Periocare Gum Massage	Periocare Gum Massage	0.5, 1 & 1.5
Suryaprasanna et al. (2018) [136]	India	RCT	0	50	Chronic PD	Non‐Smoker	NSPT w. or w/o AB		3 & 6
Tekce et al. (2015) [137]	Turkey	RCT	0	40	Chronic PD	Non‐Smoker	NSPT w. or w/o probiotic	Probiotic	3, 6 & 12
Winkel et al. (2001) [138]	Netherlands	RCT	0	49	Chronic PD	Both	NSPT w. or w/o AB		3
Yilmaz et al. (2012) [139]	Turkey	RCT	1	27	Chronic PD	N/I	NSPT w. or w/o AB or laser	Laser	3
Yilmaz et al. (2013) [140]	Tureky	RCT	1	30	Chronic PD	Non‐Smoker	NSPT w. or w/o laser or ozone	Laser or ozone	3
Ali et al. (1992) [141]	Norway	CT	1	10	Chronic PD	N/I	NSPT vs. surgery	CHX	3
Preber et al. (1995) [5]	Sweden	CCS	0	32	Chronic PD	Case–Control	NSPT, smokers vs. non‐smokers		2
Renvert et al. (1998) [142]	Sweden	CCS	0	28	Chronic PD	Both	NSPT, smokers vs. non‐smokers		6
**16S rRNA gene sequencing studies**									
Bizzarro et al. (2016) [109]	Netherlands	RCT	No	37	Chronic PD	Both	NSPT w o w/o AB	CHX	3 & 6
Hagenfeld et al. (2018) [143]	Germany	RCT	No	47	Chronic PD	Non‐smokers	NSPT w o w/o AB		2
Lu et al. (2021) [144]	China	RCT	No	14	Severe PD	Non‐smokers	NSPT w o w/o AB		3 & 6
Nie et al. (2024) [36]	China	RCT	Yeso	17	Chronic PD	Non‐smokers	NSPT w o w/o PDT	PDT	2 weeks, 2 months
Belstrom et al. (2018) [29]	Denmark	Long.	No	25	Chronic PD	Both	NSPT		1.5 & 3
Izidoro et al. (2023) [145]	Portugal	Long.	No	20	Both	Both	NSPT		2
Liu et al. (2018) [146]	China	Long.	No	12	Aggr. PD	Non‐smokers	NSPT		1.5
Martelli et al. (2016) [147]	Italy	CSS	No	14	Both	Both	NSPT w o w/o laser	Laser	1–4, 4–12, 12–24

Abbreviations: AB, systemic antibiotics; Aggr., aggressive; CHX, chlorhexidine; CSS, case–control study; FMD, full‐mouth disinfection; Long., longitudinal study; mths., months; N/I, no information; NSPT, non‐surgical periodontitis therapy; PC, professional weekly plaque control; PD, periodontal disease; PDT, photodynamic therapy; PVP, Povidone‐iodine; RA, rheumatoid arthritis; RCT, randomised clinical trial; w or w/o, with or without; wks, weeks.

#### 3.2.2 Sampling and Primary Outcomes Reporting

Sampling was performed either with sterile curettes (68%) [37, 41, 44, 45, 46, 47, 48, 49, 50, 51, 52, 53, 54, 58, 59, 61, 62, 63, 64, 65, 66, 67, 68, 71, 72, 73, 74, 77] or paper points (26%) [38, 39, 40, 42, 43, 55, 57, 60, 69, 70, 76], whereas two studies did not provide information about the sampling procedure (5%) [56, 75]. Most studies (83%) analysed site‐specific subgingival plaque samples [40, 41, 44, 45, 46, 47, 48, 49, 50, 51, 52, 53, 54, 55, 56, 57, 58, 59, 60, 61, 63, 64, 65, 66, 67, 68, 69, 70, 72, 73, 74, 77], six studies (15%) analysed pooled samples, [37, 38, 39, 42, 43, 76] and one study (2%) did not provide further information [75].

Twenty‐three studies (56%) reported microbiological data on the whole checkerboard DNA–DNA hybridisation technology panel [41, 44, 45, 47, 48, 49, 50, 51, 52, 55, 58, 59, 60, 62, 63, 64, 65, 66, 68, 71, 72, 73, 74]; seven studies (41%) reported on selected species [37, 38, 39, 40, 42, 43, 53, 54, 56, 57, 61, 67, 69, 70, 75, 76, 77] and one study (2%) [46] reported the mean Socransky complexes [8]. Appendix S6 provides details on primary outcome reporting of studies using checkerboard DNA–DNA hybridisation technology, including the specific reasons for exclusion of studies from the meta‐analysis.

#### 3.2.3 Changes in Mean Counts of Reported Species

**FIGURE 1:**
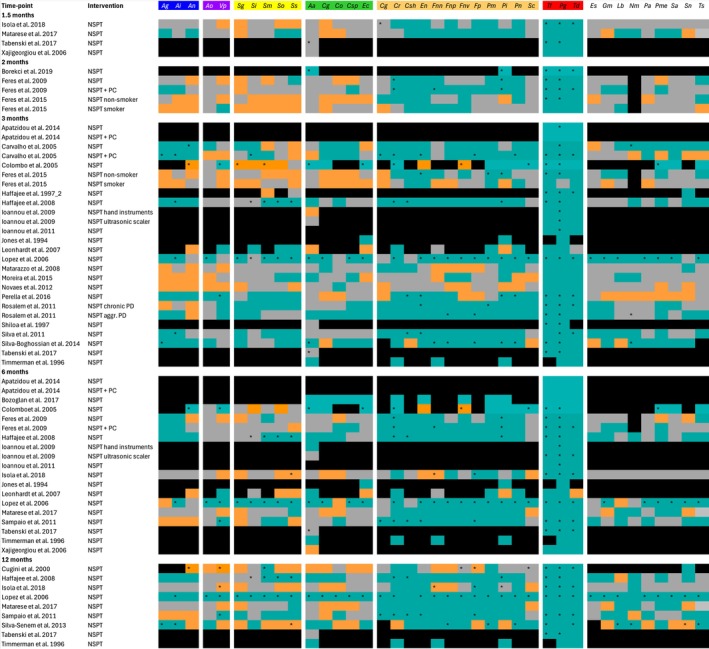
Microbiological findings of checkerboard DNA–DNA hybridisation technology studies without adjunctive therapy at various time‐points after NSPT. Turquoise indicates a decrease in mean counts, grey indicates no changes in mean counts, and orange indicates an increase in mean counts of subgingival bacteria after NSPT, black indicates that microbiological data is not reported, * indicates results that were reported as statistically significant (*p* value ≤ 0.05). PC, Weekly professional plaque control; PD, Periodontitis. Bacterial abbreviations: *Ag*, *A. gerencseriae* ; *Ai*, *A. israelli*; *An*, *A. naeslundii* ; *Ao*, *A. odontolyticus* ; *Vp*, *V. parvula* ; *Sg*, *S. gordonii* ; *Si*, *S. intermedius* ; *Sm*, *S. mitis* ; *So*, *S. oralis* ; *Ss*, *S. sanguis*; *Aa*, *A. actinomycetemcomitans* ; *Cg*, *C. gingivalis* ; *Co*, *C. ochracea* ; *Csp*, *C. sputigena* ; *Ec*, *E. corrodens* ; *Cr*, *C. rectus* ; *Csh*, *C. showae* ; *En*, *E. nodatum* ; *Fnn*, *F. nucleatum* ; *Fnp*, *F. n. polymorphum*; *Fnv*, *F. n. vincentii*; *Fp*, *F. periodonticum* ; *Pm*, *P. micra* ; *Pi*, *P. intermedia* ; *Pn*, *P. nigrescens* ; *Sc*, *S. constellatus* ; *Tf*, *T. forsythia* ; *Pg*, *P. gingivalis* ; *Td*, *T. denticola* ; *Es*, *E. saburreum* ; *Gm*, *G. morbillorum* ; *Lb*, *L. buccalis* ; *Nm*, *N. mucosa* ; *Pa*, *P. acnes* ; *Pme*, *P. melaninogenica* ; *Sa*, *S. anginosus* ; *Sn*, *S. noxia* ; *Ts*, *T. socranskii*.

Figure 1 illustrates changes in mean counts of subgingival microbiota measured with checkerboard DNA–DNA hybridisation technology at different time points after NSPT in studies without the use of antimicrobial adjunctive therapies. The follow‐up time ranged from 1.5 months to 12 months after NSPT and showed a similar pattern of change in mean counts of bacterial species. Therefore, only the changes in mean counts of bacterial species at the most common time point (at 3 months after NSPT) are described in more detail below.

Mean counts of the species belonging to the blue complex showed a decrease in 48%, an increase in 39% and no change in 11% of the treatment arms. Mean counts of species belonging to the purple complex showed a decrease in 29%, an increase in 25% and no change in 45% of the treatment arms. Mean counts of species belonging to the yellow complex showed a decrease in 43%, an increase in 20% and no change in 38% of the treatment arms. Mean counts of species belonging to the green complex showed a decrease in 40%, an increase in 16%, and no change in 41% of the treatment arms. Mean counts of *A. actinomycetemcomitans* were reported in 21 treatment arms, of which a decrease in mean counts was reported in 33%, an increase of *A. actinomycetemcomitans* in 14%, and no change of mean counts of *A. actinomycetemcomitans* in 52% of the treatment arms. Mean counts of species belonging to the orange complex showed a decrease in 68%, an increase in 14% and no change in 18% of the treatment arms. Species belonging to the red complex presented a consistent decrease in mean counts after NSPT; 3 months after NSPT, 97% of the treatment arms showed a decrease in mean counts, and 2% showed no change. Lastly, mean counts of other species or species belonging to the white complex showed a decrease in 45%, an increase in 45%, and no change in 14% of the treatment arms.

Furthermore, the influence of the periodontal diagnosis and the influence of smoking status on mean count of subgingival microbiota was evaluated in studies without adjunctive therapies at 3 months after NSPT. Patients with the diagnosis of aggressive periodontitis and patients with the diagnosis of chronic periodontitis showed a similar pattern of changes in mean counts of subgingival microbiota induced by NSPT. When comparing the data reported from studies which included smokers versus studies which did not include smokers, noticeably, the studies with smokers did not report any statistically significant changes over time. In addition, a decrease in mean counts after NSPT was reported for fewer species in the smoking subjects. However, these findings are based on only two studies which included only smokers (Appendix S7).

In addition, studies which used adjunctive therapy were compared with studies which did not use such adjunctive therapy; they showed a similar pattern of changes in mean counts of subgingival microbiota induced by NSPT at 3 months (Appendix S8).

Lastly, the influence of supportive periodontal care (SPC) provision during the clinical trial on changes in mean counts was investigated in the pool of studies which did not use any adjunctive therapy at 6 and 12 month follow‐ups after NSPT. It was noticeable that at both time points, studies with patients receiving SPC reported more frequently significant findings of changes in mean counts and reported more frequently a decrease in the mean counts of disease‐associated bacterial species (Appendix S9).

#### 3.2.4 Changes in Relative Proportions of Socransky Complexes

Twelve checkerboard DNA–DNA hybridisation technology studies [41, 46, 47, 48, 58, 59, 62, 63, 65, 66, 68, 74] reported changes to relative proportions of Socransky complexes [8] (Appendix S10). Averages for all time points were calculated. Qualitative analysis showed a decrease in red complex bacteria combined with an increase in *actinomyces* spp. (blue) and other (white) bacteria at 3, 6, 9 and 12 months after NSPT (Appendix S11).

#### 3.2.5 Meta‐Analyses

Twenty‐six checkerboard DNA–DNA hybridisation technology studies [37, 38, 40, 41, 42, 43, 44, 47, 48, 51, 52, 53, 54, 55, 58, 60, 62, 63, 64, 65, 66, 67, 68, 69, 72, 74, 76] were included in a longitudinal model comparing mean counts at baseline with various follow‐up time points. The meta‐analysis showed a significant decrease in mean counts of *A. actinomycetemcomitans* at 3 months after NSPT. The subgroup analysis between studies with and without the use of adjunctive therapy showed similar effect sizes for both groups.

The meta‐analysis also showed a significant decrease in mean counts of *P. gingivalis* and *T. forsythia* at 3 and 6 months after NSPT. The subgroup analysis between NSPT studies with and without the use of adjunctive therapy showed smaller effect sizes for *P. gingivalis* and *T. forsythia* in the studies using adjunctive therapy, but the difference did not reach statistical significance.

A significant decrease in mean counts of *T. denticola* in studies without the use of adjunctive therapy at 3 months after NSPT was found. Furthermore, it showed a significant decrease in mean counts of *T. denticola* 6 months after NSPT in both studies with and without the use of adjunctive therapies. The subgroup analysis between studies with and without the use of adjunctive therapies for the 6 month time point showed slightly smaller effect sizes for the studies using adjunctive therapies; the difference did not reach statistical significance.

The meta‐analysis showed a significant decrease in mean counts of *E. nodatum* and *P. intermedia* at 3 months after NSPT and a significant decrease in mean counts of *C. rectus* at 6 months after NSPT. Table 2 shows the number of studies included in each model, effect sizes, CI and *p* values of all meta‐analyses described above. Appendix S12 includes figures of all individual forest plots.

**TABLE 2:** Meta‐analysis of checkerboard DNA–DNA hybridisation technology studies and real‐time quantitative PCR studies after applying a longitudinal random‐effects model DerSimonian‐Laird).

Species		1.5 months		3 months		6 months	
		No adjunctive therapy	Adjunctive therapy	No adjunctive therapy	Adjunctive therapy	No adjunctive therapy	Adjunctive therapy
**Checkerboard DNA–DNA hybridisation technology studies**							
*A. actinomycetemcomitans*	*N*			7	5		
	Effect size			0.68	0.7		
	CI			0.41–0.95	0.36–1.04		
	*p*			0.001	0.001		
*P. gingivalis*	*N*			17	7	11	6
	Effect size			0.78	0.67	0.85	0.61
	CI			0.56–0.99	0.39–0.95	0.44–1.22	0.23–1.22
	*p*			0.001	0.001	0.001	0.001
*T. forsythia*	*N*			11	7	10	6
	Effect size			0.85	0.78	0.81	0.7
	CI			0.51–1.18	0.51–1.18	0.49–1.14	0.29–1.12
	*p*			0.00	0.00	0.01	0.04
*T. denticola*	*N*			11		10	5
	Effect size			0.86		0.78	0.65
	CI			0.59–1.13		0.46–1.10	0.28–1.03
	*p*			0.001		0.01	0.001
*E. nodadum*	*N*			5			
	Effect size			0.61			
	CI			0.29–0.93			
	*p*			0.001			
*P. intermedia*	*N*			5			
	Effect size			0.46			
	CI			0.18–0.74			
	*p*			0.001			
*C. rectus*	*N*					5	
	Effect size					0.49	
	CI					0.22–0.75	
	*p*					0.001	
**Real‐time quantitative PCR technology studies**							
*A. actinomycetemcomitans*	*N*			14	7	10	
	Effect size			0.58	0.52	1.46	
	CI			0.08–1.07	0.26–0.78	0.51–2.40	
	*p*			0.001	0.41	0.001	
*P. gingivalis*	*N*	7	5	17	10	13	5
	Effect size	1.65	2.01	0.78	1.12	1.33	2.01
	CI	0.60–2.70	1.24–2.79	0.00–1.57	0.47–1.77	0.28–2.39	0.82–3.20
	*p*	0.001	0.001	0.001	0.001	0.001	0.001
*T. forsythia*	*N*	7	5	15	8	11	
	Effect size	2.08	1.85	1	0.48	0.93	
	CI	1.07–3.09	0.85–2.84	0.26–1.74	0.11–0.86	0.23–1.64	
	*p*	0.001	0.001	0.001	0.01	0.01	
*T. denticola*	*N*			9	6	6	
	Effect size			1.51	1.27	0.54	
	CI			0.35–2.68	0.33–2.21	−0.17–1.25	
	*p*			0.001	0.001	0.001	

*Note:* Results of longitudinal meta‐analyses at different time‐point after NSPT: *N*, number of studies which reported on the specific follow‐up time‐point included into the model all empty fields did either not report on the species at this time‐point or < 5 studies reported on the species, effect size: Hedges's g, CI, confidence interval, *p* values highlighted in red were not significant.

##### 3.2.5.1 The Influence of CHX Use on Mean Counts of *P. gingivalis* Following NSPT

**FIGURE 2:**
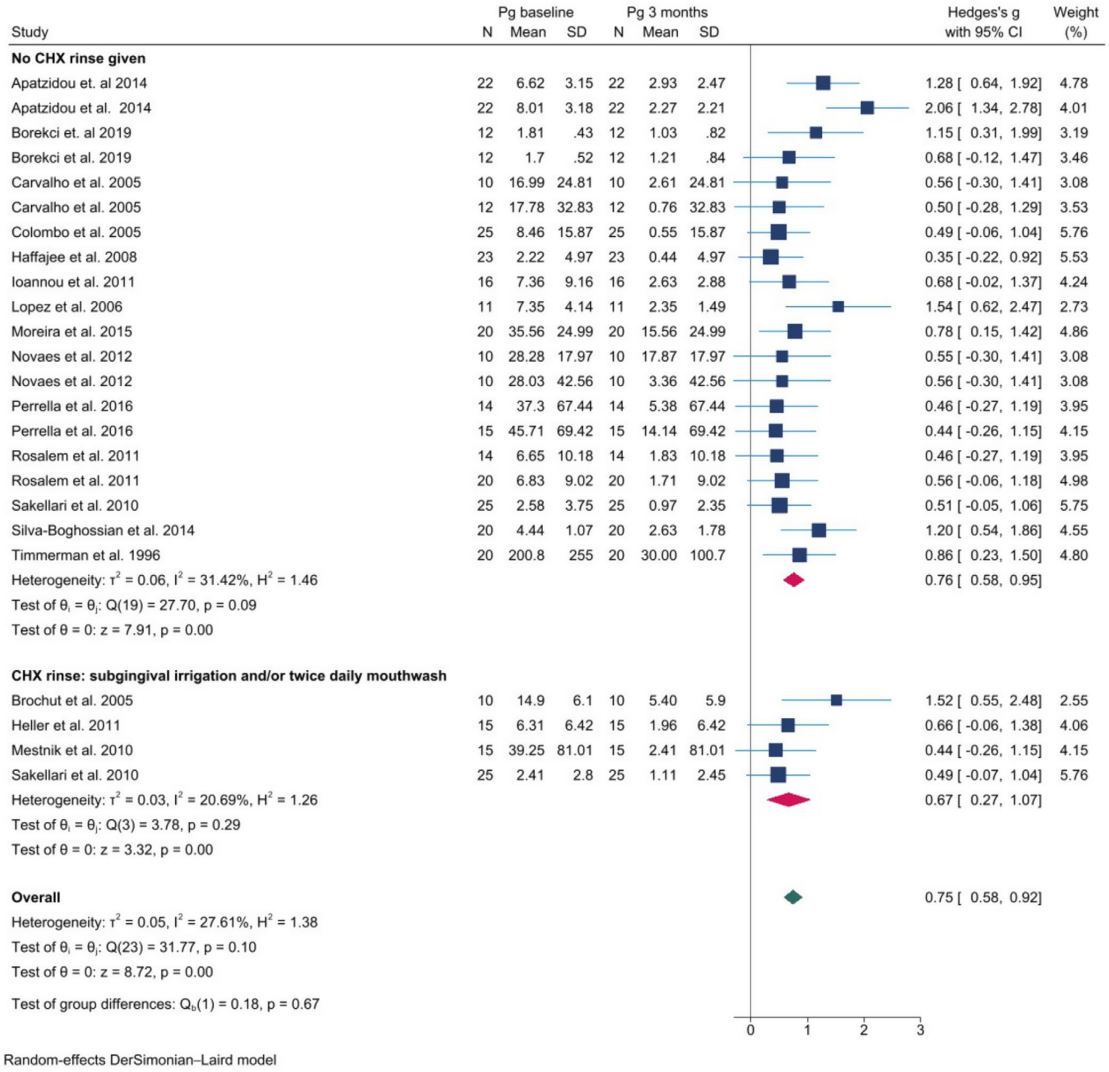
Longitudinal meta‐analysis of checkerboard DNA–DNA hybridisation technology studies comparing mean counts of *P. gingivalis* (Pg) at baseline and at 3 months after NSPT. The subgroup analysis differentiates between studies with and without the use chlorhexidine (CHX). Squares on the left side of the vertical zero line indicate fewer bacteria found at baseline, squares on the right side of the vertical zero line indicate fewer bacteria found 3 months after NSPT. The red rhombus indicates the effect size for each group independently, the green rhombus the combined effect size. No significant difference was found between the subgroup (red) and combined (green) effect sizes.

Meta‐analysis comparing checkerboard DNA–DNA hybridisation technology studies that used [40, 52, 62] or that did not use [37, 38, 41, 44, 51, 54, 58, 63, 64, 65, 66, 72, 76] chlorhexidine either as a subgingival irrigation during treatment and/or as a twice daily mouthwash in the weeks following the treatment was performed on mean counts of *P. gingivalis* at 3 months after NSPT. The meta‐analysis showed an overall significant decrease in mean counts of *P. gingivalis* after NSPT, but the use of CHX did not demonstrate a significant additional effect on this change (Figure 2).

In a complementary sensitivity analysis, the specific CHX applications in each study were examined. Out of the seven studies that applied CHX, one study used a subgingival Periochip [67], two studies applied CHX 0.1% as a mouthwash for 1 to 7 days following NSPT, [40, 43] and four studies applied CHX 0.1% mouthwash long‐term, for 6 to 8 weeks following NSPT [47, 52, 62, 74]. An additional meta‐analysis on the studies only using long‐term CHX mouthwash application was not performed because there were less than five studies that could be included.

##### 3.2.5.2 The Influence of Supportive Periodontal Care (SPC) on Mean Counts of Subgingival Microbiota Following NSPT

To test the influence of SPC on mean counts of subgingival microbiota following NPST, a subgroup meta‐analysis was performed in the cohort of checkerboard DNA–DNA hybridisation technology studies. The subgroup analysis was performed for *P. gingivalis* , *T. denticola,* and *T. forsythia* at 6 months following NSPT. In studies where SPC was provided, all three bacterial species presented with higher effect sizes than in the studies in which SPC was not provided. The effect reached statistical significance for *P. gingivalis* and *T. denticola* , but not for *T. forsythia* (Appendix S13).

### 3.3 Results From Studies Employing Real‐Time Quantitative PCR Technology

#### 3.3.1 Study Characteristics and Treatment Modalities

Thirty studies [79, 81, 82, 83, 84, 85, 86, 87, 88, 89, 90, 91, 92, 93, 94, 95, 96, 97, 98, 99, 100, 101, 102, 103, 104, 105, 106, 107, 108, 148] used real‐time quantitative PCR as the technique to measure mean counts of subgingival microbiota before and after NSPT [81, 82, 83, 84, 85, 86, 87, 88, 89, 90, 91, 92, 93, 94, 95, 96, 97, 98, 99, 100, 101, 102, 103, 104, 106, 107, 108, 148, 149]. Out of the 30 studies, 24 studies (80%) were RCTs [79, 81, 82, 83, 84, 85, 86, 87, 88, 89, 90, 91, 92, 93, 94, 95, 96, 97, 98, 99, 100, 102, 103, 148], five studies (17%) were CSSs [101, 105, 106, 107, 108] and one study (3%) had a prospective longitudinal single‐arm design [104]. Four studies (13%) [82, 94, 95, 98] used a split‐mouth design to compare the different treatment modalities, whereas the other 26 studies (87%) used whole‐mouth study designs [81, 83, 84, 85, 86, 87, 88, 89, 90, 91, 92, 93, 96, 98, 99, 100, 103, 148].

Based on the 1999 WWP classification [78], 26 studies (87%) included patients with a diagnosis of chronic periodontitis [81, 83, 84, 86, 87, 88, 89, 90, 91, 92, 93, 94, 95, 96, 97, 98, 99, 100, 101, 102, 103, 104, 105, 106, 148], three studies (10%) included patients with aggressive periodontitis, [79, 82, 85] and one study included patients with both (3%) [107]. The sample size ranged from 10 [81] to 130 [107] subjects.

Subgingival microbiota were assessed before and after NSPT was performed. Various adjunctive therapies were applied in addition to mechanical NSPT, including CHX (13%) [79, 87, 106, 108], laser (3%) [82, 88, 98, 103], PDT (10%) [93, 95, 102], PVP (3%) [85], probiotics (3%) [148], ozone (3%) [91], hyaluronic acid (3%) [86], boric acid (3%) [97], and erythritol (3%) [94].

The most common time point to measure subgingival microbiota was at 3 months after NSPT in 23 studies (77%) [81, 82, 83, 84, 85, 86, 87, 88, 89, 93, 94, 95, 97, 98, 99, 102, 103, 105, 106, 107, 108, 148, 149], followed by at 6 months in 13 studies (43%) [83, 86, 87, 89, 90, 92, 93, 96, 99, 102, 103, 106, 108], at 1.5 months in six studies (20%) [85, 90, 97, 100, 101, 148], at 1 month in three studies (10%) [91, 94, 95], and at 2 [91], 9 [108] and 12 [89] months in one study (3%) each. Table 1 provides information about the study characteristics and treatment modalities of the 30 studies [79, 81, 82, 83, 84, 85, 86, 87, 88, 89, 90, 91, 92, 93, 94, 95, 96, 97, 98, 99, 100, 101, 102, 103, 104, 105, 106, 107, 108, 148] which used real‐time quantitative PCR technology to measure subgingival microbiota before and after NSPT [81, 82, 83, 84, 85, 86, 87, 88, 89, 90, 91, 92, 93, 94, 95, 96, 97, 98, 99, 100, 101, 102, 103, 104, 106, 107, 108, 148, 149].

#### 3.3.2 Sampling and Primary Outcome Reporting

Sampling was performed either with paper points (73%) [79, 81, 82, 83, 84, 85, 86, 87, 88, 89, 90, 91, 93, 95, 100, 101, 102, 103, 104, 105, 106, 107], with sterile curettes (17%) [94, 96, 97, 98, 148] or with both (7%) [92, 108]. One study did not provide information about the sampling procedure (3%) [99]. Fourteen studies (47%) analysed site‐specific subgingival plaque samples [79, 82, 84, 85, 90, 91, 92, 93, 94, 96, 97, 98, 101, 102]; 12 studies (40%) analysed pooled samples [81, 83, 86, 87, 95, 100, 103, 105, 106, 107, 108, 148], and four studies (13%) did not provide any further information [88, 89, 99, 104].

Real‐time quantitative PCR technologies were used to investigate the effect of NSPT on subgingival microbiota on the following bacterial species: *Actinomyces naeslundii* ( *A. naeslundii* ), *Streptococcus oralis* ( *S. oralis* ), *A. actinomycetemcomitans* , *Eikenella corrodens* ( *E. corrodens* ), *Capnocytophaga gingivalis* ( *C. gingivalis* ), *Campylobacter rectus* ( *C. rectus* ), *Fusobacterium nucleatum* ( *F. nucleatum* ), *Eubacterium nodatum* ( *E. nodatum* ), *P. intermedia* , *Prevotella nigrescens* ( *P. nigrescens* ), *T. forsythia* , *P. gingivalis* and *T. denticola* . Appendix S14 provides details on primary outcome reporting of real‐time quantitative PCR studies, including the specific reasons for exclusion of studies from the meta‐analysis.

#### 3.3.3 Changes in Mean Counts of Reported Species

**FIGURE 3:**
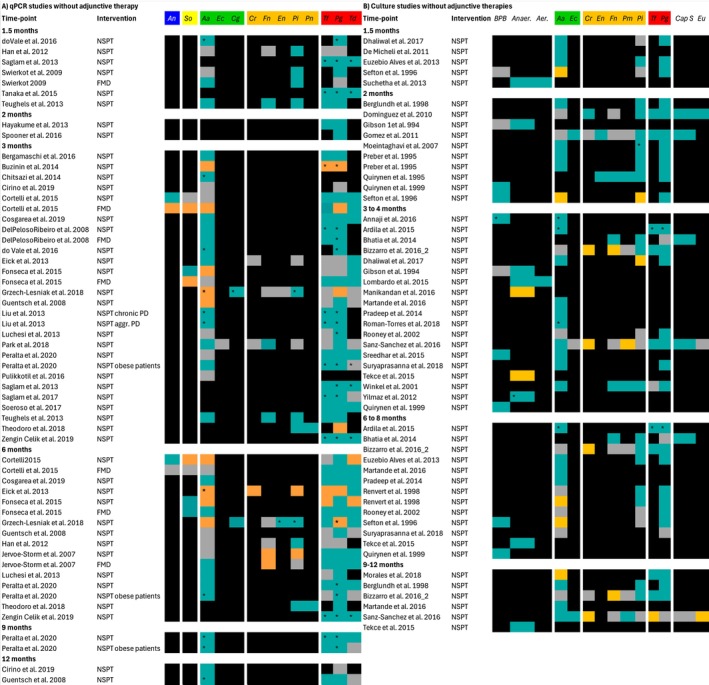
Microbiological findings of real‐time quantitative PCR studies (A) and microbial culture studies (B) without adjunctive therapy at various time‐points after NSPT. Turquoise indicates a decrease in mean counts, grey indicates no changes in mean counts and orange an increase in mean counts of subgingival bacteria after NSPT, black indicates bacteria are not reported, * indicates results, that were reported as statistically significant (*p* value ≤ 0.05), FMD: Full‐mouth disinfection. Bacterial abbreviations: *An*, *A. naeslundii* ; *So*, *S. oralis* ; *Aa*, *A. actinomycetemcomitans* ; *Cg*, *C. gingivalis* ; *Ec*, *E. corrodens* ; *Cg*, *C. gracilis* ; *Cr*, *C. rectus* ; *En*, *E. nodatum* ; *Fn*, *F. nucleatum* ; *Pi*, *P. intermedia* ; *P.m*, *P. micra* ; *Pn*, *P. nigrescens* ; *Tf*, *T. forsythia* ; *Pg*, *P. gingivalis* ; *Td*, *T. denticola* ; *BPB*, *black‐pigmented bacteria*; *Eu*, *Eubacterium* spp. and *Cap*, *Capnocytophaga* spp.

Figure 3A illustrates changes in mean counts of various subgingival bacterial species measured with real‐time quantitative PCR at different follow‐up time‐points after NSPT without the use of antimicrobial adjunctive therapies. Follow‐up time points ranged from 1.5 months to 12 months after NSPT and showed a similar pattern of changes in mean counts of bacterial species. The most frequently reported follow‐up time point was at 3 months after NSPT. Therefore, this time point is described in more detail below.

In studies without adjunctive therapy, changes in mean counts of *A. actinomycetemcomitans* after NSPT were reported in 23 treatment arms of the real‐time quantitative PCR studies, of which 12 treatment arms (52%) [81, 82, 84, 85, 86, 94, 106, 107, 108, 148] reported a decrease, five (22%) [83, 87, 88, 89, 105] reported an increase and six (26%) [79, 83, 87, 93, 95, 108] reported no change in mean counts of *A. actinomycetemcomitans* after NSPT.

Changes in mean counts of *P. gingivalis* were reported in 26 treatment arms, of which 19 treatment arms (73%) [81, 83, 84, 85, 87, 89, 93, 94, 97, 99, 103, 106, 107, 108, 148] reported a decrease, four (16%) [83, 88, 102, 105] reported an increase, and three (12%) [79, 86, 87] reported no change in mean counts of *P. gingivalis* after NSPT.

Changes in mean counts of *T. forsythia* were reported in 23 treatment arms, of which 15 study arms (65%) [81, 83, 84, 87, 97, 99, 103, 106, 107, 108, 148] reported a reduction, one study arm (4%) [105] an increase, and six study arms (26%) [86, 87, 88, 89, 93, 94] reported no change in mean counts after NSPT.

Changes in mean counts of *T. denticola* were reported in 15 treatment arms, of which 10 treatment arms (67%) [83, 86, 87, 97, 99, 103, 106, 108] reported a decrease in mean counts of *T. denticola* , and five treatment arms (33%) [88, 89, 94, 98, 108] reported no change in mean counts after NSPT.

Other species were less commonly reported. Changes in mean counts of *A. naeslundii* were reported in two treatment arms, one time with an increase and one time with a reduction in mean counts after NSPT [83]. Changes in mean counts of *S. oralis* were reported in four treatment arms, one time with no change [83], two times with an increase [83, 87], and one time with a reduction [87] in mean counts after NSPT. Changes in mean counts of *E. corrodens* were reported in one treatment arm with no change in mean counts after NSPT [94]. Changes in mean counts of *C. gingivalis* were reported in one treatment arm with a significant decrease in mean counts after NSPT [86]. Changes in mean counts of *C. rectus* were reported in two treatment arms with no change in mean counts after NSPT [86, 94]. Changes in mean counts of *F. nucleatum* were reported in three treatment arms, one time with no change in mean counts [88] and two times with a reduction in mean counts after NSPT [94, 148]. Changes in mean counts of *E. nodatum* were reported in one treatment arm with no change in mean counts after NSPT [88]. Changes in mean counts of *P. intermedia* were reported in five treatment arms, three times with a reduction in mean counts [88, 102, 148] and twice with no change in mean counts after NSPT [85, 94]. Finally, changes in mean counts of *P. nigrescens* were reported in one treatment arm with a reduction in mean counts after NSPT [102].

Furthermore, the influence of smoking status on mean counts of subgingival microbiota at 3 months after NSPT in studies without adjunctive therapy was evaluated. Studies showed a similar pattern in changes to mean counts of subgingival microbiota, but studies with only smokers reported no statistically significant results. However, these findings are based on only two studies. In addition, the influence of periodontal diagnosis on mean counts of subgingival microbiota at 3 months after NSPT in studies without adjunctive therapy was examined. These studies showed a similar pattern in changes to mean counts of subgingival microbiota induced by NSPT.

Moreover, the influence of supportive periodontal care (SPC) provision during the clinical trial on changes in mean counts was investigated in the pool of studies that did not use any adjunctive therapies at 6 month follow‐up after NSPT. It was noticeable that at both time‐points, studies with patients receiving SPC reported more often significant findings of changes in mean counts and reported more often a decrease in the mean counts of disease‐associated bacterial species.

The influence of adjunctive therapies on mean counts of subgingival microbiota at 3 months after NSPT was also evaluated. The studies that used adjunctive therapies presented a similar pattern in changes to mean counts of subgingival microbiota to studies that did not use such adjunctive therapies (Appendix S15A–D).

#### 3.3.4 Meta‐Analyses

Seventeen real‐time quantitative PCR studies were included in the long meta‐analyses [81, 84, 85, 87, 90, 93, 94, 95, 97, 98, 101, 102, 105, 106, 108, 148]. The meta‐analysis showed a significant decrease in mean counts of *A. actinomycetemcomitans* three and 6 months after NSPT. A subgroup analysis between studies with or without adjunctive therapy was performed for 3 months after NSPT and showed a similar effect size in both groups.

The meta‐analysis showed a significant decrease in mean counts of *P. gingivalis* and *T. forsythia* at 1.5 months, 3 months, and 6 months after NSPT. The subgroup analysis on *P. gingivalis* between studies with and without the use of adjunctive therapy showed larger effect sizes for the studies using adjunctive therapy for all three time‐points (no statistical difference). The subgroup analysis between studies with and without adjunctive therapy on *T. forsythia* showed smaller effect sizes for the studies using adjunctive therapy at 1.5 and 3 months after NSPT (no statistical difference).

A significant decrease in mean counts of *T. denticola* at 3 and 6 months after NSPT was also observed. The subgroup analysis for the 3‐month time point showed a slightly smaller effect size for the studies using adjunctive therapy, but the difference did not reach statistical significance. Table 2 shows the number of studies included in the model, effect sizes, CI and *p* values of all meta‐analyses described above. Appendix S16 includes figures of all individual forest plots.

### 3.4 Results From Studies Employing Bacterial Culture Technology

#### 3.4.1 Study Characteristics and Treatment Modalities

Thirty‐six studies [6, 109, 110, 112, 113, 114, 115, 116, 117, 118, 119, 120, 121, 122, 123, 124, 125, 126, 127, 128, 129, 130, 131, 132, 133, 134, 135, 136, 137, 138, 139, 140, 141, 142, 149, 150] used bacterial culture techniques to measure mean counts of cultivable subgingival bacteria. Thirty‐three studies (92%) were RCTs [6, 109, 110, 111, 112, 113, 114, 115, 116, 117, 118, 119, 120, 121, 122, 123, 124, 125, 126, 127, 128, 129, 130, 131, 132, 133, 134, 135, 136, 137, 138, 139, 140, 141, 142, 150], two studies (6%) were CSS [6, 142], and one study (3%) had a prospective single‐arm design [141]. Eleven studies (31%) [113, 114, 118, 119, 121, 122, 134, 139, 140, 141, 150] used a split‐mouth design to compare the different treatment modalities, whereas the other 25 studies (69%) [6, 109, 110, 112, 115, 116, 117, 120, 123, 124, 125, 126, 127, 128, 129, 130, 131, 132, 133, 135, 136, 137, 138, 142] used a whole‐mouth study design. The sample size ranged from 10 [141] to 230 subjects [130].

Based on the 1999 WWP classification [78], 26 studies (89%) included patients with a diagnosis of chronic periodontitis [6, 109, 112, 113, 114, 115, 116, 117, 118, 120, 121, 122, 123, 124, 125, 126, 127, 128, 129, 130, 131, 132, 133, 134, 135, 136, 137, 138, 139, 140, 141, 142], three studies (8%) [110, 111, 150] included patients with aggressive periodontitis [79, 82, 85], and one study (3%) [119] did not provide information about the diagnosis of the study participants.

Various adjunctive therapies were applied in addition to mechanical NSPT, including CHX (19%) [115, 122, 127, 128, 129, 130, 141], Curcuma (6%) [113, 134], Desiccant (3%) [121], laser (17%) [114, 117, 118, 120, 132, 139, 140, 150], PDT (3%) [150], Periocare (natural anti‐inflammatory ingredients) (3%) [135], probiotics (8%) [116, 125, 137], methylene blue gel (3%) [119], NaOCL (3%) [109] or ozone (3%) [140].

The most common follow‐up time point to measure subgingival microbiota after NSPT was at 3 months, selected in 19 studies (53%), followed by 2 months in 12 studies (33%), 6 months in 11 studies (31%), 1.5 months in four studies (11%) and 9 or 24 months in one study (3%) each.

Table 1 provides information about the study characteristics and treatment modalities of the 36 studies that used bacterial culture as the technology to measure subgingival microbiota before and after NSPT.

#### 3.4.2 Sampling and Primary Outcome Reporting

Sampling was performed either with paper points (78%) [6, 109, 110, 111, 112, 114, 115, 116, 117, 118, 119, 120, 123, 125, 126, 127, 128, 129, 130, 132, 133, 135, 137, 138, 139, 140, 141, 142] or by sterile curettes (22%) [113, 121, 122, 124, 131, 134, 136, 150]. Eighteen studies (50%) [109, 110, 111, 112, 115, 118, 123, 126, 127, 128, 129, 130, 131, 132, 137, 138, 140, 141] analysed pooled subgingival plaque samples, 13 studies (36%) [6, 114, 116, 117, 120, 121, 122, 125, 133, 134, 136, 142, 150] analysed site‐specific samples, and five studies (14%) [113, 119, 124, 135, 139] did not provide any information about the sampling procedure [88, 89, 99, 104]. Culturing times ranged from 2 days [135] to 14 days [109, 117, 125, 132, 138].

Bacterial culture technique was used to investigate the effect of NSPT on subgingival microbiota on the following bacterial species: *A. actinomycetemcomitans* , *E. corrodens* , *C. rectus* , *E. nodatum* , *F. nucleatum* , *Parvimonas micra* ( *P. micra* ), *P. intermedia* , *T. forsythia* , *P. gingivalis* , *Eubacterium* spp. *and Capnocytophaga* spp., *black‐pigmented bacteria*, *anaerobes* and *aerobes*. Appendix S17 provides details about primary outcome reporting of bacterial culture studies, including the specific reasons for exclusion of studies from the meta‐analysis.

#### 3.4.3 Changes in Mean Counts of Reported Species

Figure 3B illustrates changes in mean counts of colony forming units (CFU) of subgingival bacterial species measured with bacterial culture technology at different time points after NSPT without the use of adjunctive therapy. Follow‐up time‐points ranged from 6 weeks to 12 months. Three to 4 months was the most frequently reported follow‐up time‐point, and the reported changes are described in detail below.

*A. actinomycetemcomitans* was reported in 12 treatment arms, of which 10 treatment arms (83%) [111, 116, 123, 126, 130, 132, 134, 136, 138, 150] reported a decrease in mean counts of CFU of *A. actinomycetemcomitans* after NSPT, and two treatment arms (17%) [109, 131] reported no change in mean counts of CFU after NSPT.

*P. gingivalis* was reported in eight treatment arms, of which seven treatment arms (88%) [109, 110, 116, 131, 132, 136, 138] reported a decrease in mean counts of CFU of *P. gingivalis* after NSPT, whereas one treatment arm (13%) [113] reported no change in mean counts of CFU.

*T. forsythia* was reported in four treatment arms, of which three treatment arms (75%) [109, 110, 132] reported a decrease in mean counts of CFU of *T. forsythia* , and one treatment arm (25%) [138] reported no change in mean counts of CFU after NSPT.

*P. intermedia* was reported in six treatment arms, of which three treatment arms (50%) [109, 113, 138] reported a decrease in mean counts of CFU of *P. intermedia* , one treatment arm (17%) [116] reported an increase in mean counts of CFU, and two treatment arms (33%) [131, 132] reported no change in mean counts of CFU after NSPT.

Other species were less frequently reported. Changes in mean counts of CFU of *black‐pigmented bacteria* were reported in four treatment arms, once with no change in mean counts [119] and three times with a reduction in mean counts of CFU after NSPT [129, 134, 150]. Changes in mean counts of CFU of *anaerobes* were reported in five treatment arms, twice with an increase in mean counts of CFU [122, 137] and three times with a reduction in mean counts of CFU after NSPT [119, 121, 139]. Changes in mean counts of CFU of aerobes were reported in one treatment arm with a reduction following NSPT [121]. Changes in mean counts of CFU in *E. corrodens* were reported in one treatment arm with no change in mean counts after NSPT [132]. Changes in mean counts of CFU of *C. rectus* were reported in two treatment arms with an increase in mean counts after NSPT [109, 132]. Changes in mean counts of CFU of *F. nucleatum* were reported in four treatment arms, twice with a reduction in mean counts [113, 138], one with no change in mean counts [132], and once with an increase in mean counts after NSPT [109]. Changes in mean counts of CFU of *P. micra* were reported in three treatment arms, once with no change in mean counts [109], once with a reduction in mean counts [132], and once with an increase in mean counts after NSPT [138]. Changes in mean counts of CFU of *Eubacterium* spp. were reported in two treatment arms, both with a reduction in mean counts after NSPT [113, 132]. And finally, changes in mean counts of CFU of *Capnocytophaga* were reported in one treatment arm with no changes in mean counts after NSPT [132].

In addition, the influence of the smoking status on mean counts of CFU in studies without adjunctive therapies was investigated at 3 months after NSPT. When smokers were included in the study population, no significant changes in mean counts of CFU were reported (*p* ≤ 0.05). However, these findings are based on a few studies.

In addition, the influence of the periodontal diagnosis on mean CFU counts in studies without adjunctive therapies was investigated at 3 months after NSPT. Patients with aggressive periodontitis had more results in which the changes in mean CFU counts were reported as statistically significant, compared with studies on patients with chronic periodontitis. However, only two studies recruited patients exclusively with aggressive periodontitis.

Moreover, the influence of SPC provision during the clinical trial was investigated in the pool of studies which did not use any adjunctive therapy at 6–8 months follow‐up and 9–12 months follow‐up after NSPT. No difference was noticed in the reporting of changes on mean count in CFU in studies with or without SPC provision.

Finally, the influence of adjunctive therapies on mean counts in CFU of subgingival microbiota was examined. There seems to be a similar pattern in changes to mean counts in CFU in subgingival microbiota in studies with and without adjunctive therapies at 3 months after NSPT (Appendix S18A–D).

#### 3.4.4 Meta‐Analysis

Eight bacterial culture studies [109, 113, 116, 134, 138, 141, 142, 150] were included in the longitudinal meta‐analysis. At 3 months after NSPT, five studies without the use of adjunctive therapy reported on *A. actinomycetemcomitans* . The meta‐analysis showed a significant decrease in mean counts of CFU after NSPT (Appendix S19).

### 3.5 Results From Studies Employing 16S rRNA Gene Sequencing Technology

#### 3.5.1 Study Characteristics and Treatment Modalities

Eight studies using 16S rRNA gene sequencing technology fulfilled the specific inclusion criteria for the systematic review [19, 29, 30, 47, 143, 145, 146, 147]. One study (13%) [147] had a CSS design, three studies (38%) [29, 145, 146] were prospective longitudinal studies, three studies (38%) [36, 143, 144] were a RCTs, and one study (13%) [30] was a controlled trial (CT). One of the studies applied a split‐mout design.

Based on the 1999 WWP classification [78], four of these studies (50%) [29, 30, 36, 43] included patients with a diagnosis of chronic periodontitis, one study (38%) [146] included patients with aggressive periodontitis, and three studies (33%) [144, 145, 147] included both. The sample size ranged from 12 [146] to 47 [143] subjects.

All studies performed NSPT and assessed subgingival microbiota before and after treatment. Adjunctive therapies, such as Nd: YAG laser (13%) [147], CHX (13%) [30], or PDT (13%) [36], were applied in three studies.

Various follow time points were used to investigate the influence of NSPT on subgingival microbiota. Two studies (25%) reported data at 6 weeks [29, 146], three studies (38%) reported at 2 months [36, 143, 145], three studies (38%) at 3 months [29, 30, 144] and one study (13%) at 6 months [30]. One study (13%) reported the time points in ranges of 1 to 4 months, 4 to 12 months, and 12 to 24 months [147]. Table 1 provides information about the study characteristics and treatment modalities of the eight 16S rRNA gene sequencing studies.

#### 3.5.2 Sampling and Primary Outcome Reporting

Selection of sampling site and method was reported in five studies [29, 143, 145, 146, 147]. Samples were collected in two studies from the deepest pockets [29, 147], in one study from sites with a defined PPD (40%) [143] and in two studies from a specified selected area in the dentition [144, 146]. Three studies did not provide information about the sampling sites [30, 36, 145]. Information about the sampling procedures was reported in seven studies [29, 36, 143, 144, 145, 146, 147]. Six studies (63%) pooled samples before analysis [29, 143, 144, 145, 146, 147] and one study analysed site‐specific samples [36]. Samples were collected either with sterile curettes/scalers in four studies [29, 36, 144, 145] or with paper points in three studies [143, 146, 147]. Four studies reported on relative abundance of species [29, 143, 144, 146] and four studies reported on relative abundance of genera [30, 143, 144, 146]. Furthermore, various microbiome indices, which describe the structure of the overall microbial community found in the subgingival plaque samples, were used. Appendix S20 provides details about primary outcome reporting of the eight 16S rRNA gene sequencing studies.

#### 3.5.3 Microbiological Findings—Qualitative Analysis

Table 3 provides details about the qualitative analysis of microbiological findings in 16S rRNA gene sequencing studies.

**TABLE 3:** Qualitative analysis of the microbiological results from 16S rRNA gene sequencing studies.

Author (year)	*N*	Variable	Mean relative abundance after NSPT		α‐diversity	ß‐diversity
		Region	Genera	Species		
Belstrom et al. (2018) [29]	25	V3 to V4	↑ *Streptococcus* ↑ *Rothia* ↑ *Actinomyces* ↓ *Porhyromonas* ↓ *Treponema*	↑ *Rothia aeria* ↑ *Rothia dentocariosa* ↓ *P. gingivalis* ↓ *T. forsythia* ↓ *T. denticola* (at 2 weeks)	Shannon index: ↓at 2 and 6 weeks no sig. change at 3 mths.	Not reported
Bizzarro et al. (2016) [109]	19	V5 to V7	↑ *Neissera* ↑ *Streptococcus* ↑ *Rothia* ↑ *Caphnocytophaga* ↓ *Filifactor* ↓ *Tannerella* ↓ *Synergistaceae* ↓ *Porhyromonas* ↓ *Treponema*	Not reported	Shannon index: no sig. changes	Bray–Curtis dissimilarity index: sig. differences at 3 mths. PCA: sig. differences at 3 mths. Microbial co‐occurrence network: no sig. changes at 12 mths.
Hagenfeld et al. (2018) [143]	47	V3 to V4	Changes in mean RA are reported only based on differences between test and control		Species richness: no sig. changes Shannon index: no sig. changes Evenness: no sig. changes	Bray–Curtis dissimilarity index: no sig. differences PCoA: no sig. difference
Izidoro et al. (2023) [145]	20	V3 to V4	No sig. changes	↑ *C. sputigena* ↓ *F. nucleatum* ↓ *C. gingivalis* ↓ *C. showae*	Not reported	Not reported
Liu et al. (2018) [146]	12	V3 to V4	↑ *Streptococcus* ↑ *Lautropia* ↑ *Haemophilus* ↑ *Actinomyces* ↓ *Porhyromonas* ↓ *Treponema* ↓ *Fretibacterium*	↑ *H. parainfluenzae* ↑ *A. naeslundii* ↓ *P. gingivalis*	Shannon index: no sig. change Observed species index: no sig. changes Chao 1 index: no sig. changes Simpson index: no sig. changes	Correlation network: OTUs with a prevalence > 50% increased after treatment, the post‐treatment‐associated network was more complicated PCA: no sig. differences PCoA: no sig. differences
Lu et al. (2021) [144]	14	V3 to V4	↑ *Actinomyces* ↑ *Rothia* ↑ *Neisseria* ↑ *Capnocytophaga* ↑ *Lautropia* ↑ *Cardiobacterium* ↓ *Porphyromonas* ↓ *Treponema* ↓ *Filifactor* ↓ *TM7 G‐5* ↓ *Peptostreptococcaceae* ↓ *Fretibacterium* ↓ *Dialister* ↓ *Peptococcus*	Not reported	Shannon index: ↑ at 3 mths. and ↓ at 6 mths. Chao‐1 index: ↓ at 3 to 6 mths.	PCoA: sig. differences at 3 and 6 mths
Martelli et al. (2016) [147]	14	V4	Not reported	↓ *C. gracilis* ↓ *P. gingivalis* ↓ *T. forsythia* ↓ *T. denticola*	Not reported	Not reported
Nie et al. (2024) [36]	17	V3 to V4	↑ *Streptococcus* ↑ *Selenomonas* ↓ *Porhyromonas* ↓ *Fretibacterium* ↓ *Parvimonas* ↓ *Selenomonas*	Not reported	Species richness: ↓ at 2 and 8 weeks Shannon index: ↓ at 2 weeks	PCoA: sig. differences at 2 and 8 weeks Microbial co‐occurrence network:

Abbreviations: ↑, significant increase after NSPT; ↓, significant decrease after NSPT; mths., months; *N*, number of participants; PCA, principal component analysis; PCoA, principal coordinate analysis; RA, relative abundance; sig., significant.

Different variable regions of the 16S gene were chosen for the sequencing of microbial DNA. Five studies (63%) sequenced the region between V3 to V4 [29, 36, 143, 144, 145, 146]; one study (13%) sequenced the variable region V4 (17%) [147] and one study (13%) sequenced the variable region V5–V7 [30].

Four studies (50%) reported changes in relative abundance of species. They detected between 307 [146] and 507 [29] different species, based on detected OTUs in subgingival plaque samples. All reported a significant decrease of *P. gingivalis* after NSPT. Other species were reported with significant decreases or increases in relative abundance following NSPT. However, these findings were heterogeneous among the studies.

Seven studies (88%) [29, 30, 36, 143, 144, 145, 146] reported relative abundance changes in genera and detected between 87 [143] to 195 [30] genera in subgingival plaque samples. Four studies (57%) [29, 30, 36, 146] reported a significant decrease of the genus *Porphyromonas* and the genus *Treponema* [29, 30, 144, 146]. Four studies (57%) [29, 30, 36, 146] reported a significant increase of *Streptococcus*. Three studies (43%) [29, 30, 144] reported a significant increase of *Rothia*.

Changes to alpha diversity of the subgingival microbiome were described using various metrics. Shannon index was reported in six studies (75%) [29, 30, 36, 143, 144, 146]. Three studies (50%) [30, 143, 146] did not have any significant findings regarding Shannon index after treatment. One study (13%) reported a significant decrease from three to 6 months after treatment [144]. Two studies (33%) reported a significant decrease short term 2 weeks after treatment [29, 36].

Chao 1 index was reported in two studies (25%), one study reported a significant decrease from three to 6 months after treatment [144] and one study did not report significant changes [146].

Observed species and Simpson index were reported in one study (13%) with no significant changes [146].

Changes to beta diversity was analysed in five studies (63%) [30, 36, 143, 144, 146]. The Bray–Curtis dissimilarity index was considered in two studies (40%) [30, 143]. One study (20%) [30] reported a significant change after 3 months; the other study (20%) [143] reported not finding a significant change of the Bray–Curtis dissimilarity index after 2 months.

Principal component analysis (PCA) was used in two studies (25%) [30, 146] to visualise and analyse changes to the microbiome. Among the studies, only one [30] found significant changes after NSPT. Meanwhile, principal coordinate analysis (PCoA) was used in five studies (63%) [30, 36, 143, 144, 146] to visualise and analyse changes to the microbiome. Two studies (40%) found significant shifts after 8 weeks [36] and 3 and 6 months [144].

A correlation network analysis was performed in one study [146] which concluded that highly prevalent OTUs significantly increased in abundance after NSPT.

### 3.6 The Influence of Split‐Mouth Design on Microbiological Findings

The influence of study design (whole‐mouth versus split‐mouth) on mean count changes of periodontitis associated species at 3 months after NSPT alone was analysed in a longitudinal subgroup meta‐analysis of real‐time quantitative PCR and Checkerboard DNA–DNA hybridisation technology studies. When comparing whole‐mouth and split‐mouth studies, *A. actinomycetemcomitans* , *P. gingivalis* , *T. forsythia* , *T. denticola* showed bigger effect size in studies employing a whole‐mouth design. The difference between the groups was statistically significant for *T. denticola* in the cohort of real‐time quantitative PCR studies (*p* = 0.03) (Appendix S21).

### 3.7 Secondary Outcome Reporting and Clinical Findings Across All Four Microbiological Detection Technologies

Most studies reported on gingival inflammation by using various indices: Full‐mouth bleeding on probing (full‐mouth bleeding scores, FMBS) (71%) [29, 30, 37, 39, 40, 41, 44, 45, 46, 47, 48, 49, 50, 51, 52, 55, 56, 57, 58, 59, 60, 61, 62, 63, 64, 65, 66, 67, 68, 71, 72, 73, 74, 75, 77, 79, 82, 84, 85, 86, 88, 89, 90, 91, 92, 93, 94, 95, 97, 98, 100, 101, 102, 103, 104, 105, 106, 109, 110, 111, 112, 114, 117, 118, 119, 120, 121, 122, 124, 125, 126, 131, 132, 137, 141, 142, 143, 147, 148, 150]; Gingival index (GI) [151] (14%) [6, 69, 70, 87, 108, 116, 119, 121, 122, 123, 127, 130, 135, 136, 137, 139]; Papilla bleeding index (PBI) [152] (4%) [42, 43, 75, 76, 103]; Gingival bleeding index (GBI) [152] (4%) [38, 53, 54, 81, 146]; Sulcus bleeding index (SBI) [152] (4%) [113, 129, 133, 134, 138, 140, 150]; or Bleeding index (BI) [153] (3%) [133, 138, 146]. Five studies (4%) [83, 96, 115, 128, 145] did not report on gingival inflammation.

Supragingival plaque levels were reported on using various indices: Full‐mouth plaque scores (FMPS) (42%) [29, 30, 37, 41, 44, 45, 46, 47, 48, 49, 50, 51, 52, 53, 54, 55, 57, 58, 59, 60, 61, 62, 63, 65, 68, 71, 72, 73, 74, 79, 85, 86, 88, 89, 90, 92, 93, 94, 95, 101, 105, 106, 109, 110, 111, 125, 142, 148], Plaque index (PI) (37%) [6, 38, 39, 40, 56, 64, 69, 70, 76, 83, 87, 97, 98, 99, 108, 112, 113, 114, 116, 117, 118, 119, 120, 122, 123, 124, 126, 127, 129, 130, 131, 132, 133, 134, 135, 137, 138, 139, 140, 146, 150, 154], Approximal plaque index (API) (4%) [42, 43, 75, 100] and Visual plaque index (VPI) (3%) [81, 84, 121]. Sixteen studies (14%) [56, 66, 67, 77, 82, 91, 96, 102, 104, 107, 115, 129, 136, 141, 143, 145, 147] did not report on supragingival plaque levels of study participants.

Full‐mouth probing pocket depths (FM‐PPD) were reported in 107 studies (95%) [6, 14, 38, 39, 40, 41, 42, 43, 44, 45, 46, 47, 48, 49, 50, 51, 52, 53, 54, 55, 56, 57, 58, 59, 60, 61, 62, 63, 64, 65, 66, 67, 68, 71, 72, 74, 76, 77, 79, 81, 82, 83, 84, 85, 86, 87, 88, 89, 90, 91, 92, 93, 94, 95, 96, 97, 98, 99, 100, 101, 102, 103, 104, 105, 106, 107, 108, 109, 110, 111, 112, 113, 114, 115, 116, 117, 118, 119, 120, 121, 122, 123, 124, 125, 126, 127, 128, 129, 130, 132, 133, 134, 136, 137, 138, 139, 140, 141, 142, 148, 150] and Full‐mouth clinical attachment level (FM‐CAL) was reported in 85 studies (75%) [37, 38, 39, 41, 44, 45, 46, 47, 48, 49, 50, 51, 52, 53, 54, 55, 58, 59, 60, 61, 62, 63, 64, 65, 66, 67, 68, 71, 72, 74, 76, 77, 79, 82, 83, 86, 87, 88, 89, 90, 91, 94, 95, 96, 97, 98, 99, 100, 101, 102, 103, 104, 105, 106, 107, 108, 109, 110, 111, 112, 113, 114, 116, 117, 118, 121, 123, 124, 125, 126, 127, 129, 130, 132, 136, 138, 139, 140, 142, 148, 150].

The results indicate a reduction in FMBS, FMPS, FM‐PPD and FM‐CAL at 3 months after NSPT. Heterogeneity was noted across the studies regarding supragingival plaque; at 3 months after NSPT, FMPS was ranging from 10% to 64%. Please refer to Appendix S22 and S23 for secondary outcome reporting and illustration of the clinical changes after NSPT of the included studies.

### 3.8 Risk of Bias Assessment of Checkerboard DNA–DNA Hybridisation Technology Studies, Real‐Time Quantitative PCR Studies, Bacterial Culture Studies and 16S rRNA Gene Sequencing Studies

Risk of bias for all randomised trials was analysed using the Cochrane RoB2 tool for randomised controlled trial.

In the cohort of checkerboard DNA–DNA hybridisation technology RCTs, one study was graded as high risk because of concerns regarding the randomisation process [54] and eight studies had some concerns because of the randomisation process, due to deviations from the intended intervention and/or due to the measurement of the outcome [38, 56, 57, 58, 61, 64, 67, 70]. In the cohort of real‐time quantitative PCR RCTs, there were some concerns in eight studies with the randomisation process, and/or the measurement of the outcome [82, 88, 94, 95, 96, 99]. In the cohort of bacterial culture RCTs, there were some concerns in 13 studies with the randomisation process, and/or with the measurement of the outcome [112, 113, 115, 116, 122, 127, 129, 130, 133, 134, 135, 136]. Finally, in the cohort of 16S rRNA gene sequencing RCT study, no risk of bias was raised (Appendix S24A–D).

Risk of bias for all other study designs was assessed using the ROBINS‐1 tool. In the cohort of checkerboard studies, three studies were graded high risk because of concerns regarding measurement of the outcome [39] and/or the classification of the intervention [49, 50] and in two studies there were concerns regarding measurement of the outcome [44, 45]. In the cohort of qPCR studies, one study was graded high risk because of concerns regarding the measurement of the outcome [104], and one study was graded moderate risk for concerns in the same domain [108]. In the cohort of bacterial culture studies, one study was graded high risk because of concerns regarding the measurement of the outcome [141], and two studies were graded moderate risk for concerns in the same domain [6, 142]. In the cohort of 16S rRNA gene sequencing studies, one study was graded high risk because of concerns regarding the measurement of the outcome and lack of adjustment for smoking as a confounding factor [147]. Two studies were graded moderate risk for concerns with measuring the outcome and lack of adjustment for smoking as a confounding factor [29, 145] (Appendix S25A–D).

Sample size calculation across all four microbiological detection technologies was not reported frequently or was only based on clinical outcomes. Few studies used sample size calculation based on microbiological outcomes. Among checkerboard DNA–DNA hybridisation technology studies, 51% did not report sample size calculations, 39% used either CAL or PPD, and 10% used microbiological outcomes for it. Among the real‐time quantitative PCR studies, 30% did not report sample size calculation, 60% used clinical outcomes, such as CAL or PPD, no studies used microbiological outcomes, and 10% used other outcomes, such as c‐reactive protein. Among checkerboard DNA–DNA hybridisation technology studies, 51% did not report sample size calculation, 39% used either CAL or PPD, and 10% used microbiological outcomes. Sixty‐four percent of the bacterial culture studies did not report sample size calculation, 33% used clinical outcomes, such as CAL or PPD, no studies used microbiological outcomes, and 3% used another outcome not further clarified in the study. Meanwhile, among checkerboard DNA–DNA hybridisation technology studies, 51% did not report sample size calculations, 39% used either CAL or PPD, and 10% used microbiological outcomes for calculating sample size. Among the 16S rRNA gene sequencing studies, 63% did not report sample size calculation, one study used PPD, and two studies used microbiological outcomes for sample size calculation. One study used expected changes to alpha diversity in saliva as the microbiological outcome [29] and one study used predicted changes to mean relative abundance of *P. gingivalis* as the microbiological outcome [144].

## 4 Discussion

To the best of the authors' knowledge, this is the first systematic review and meta‐analysis that comprehensively summarises the results of studies which measured the effect of NSPT on subgingival microbiota with various microbiological technologies. Changes to subgingival microbiota following NSPT were assessed in studies using checkerboard DNA–DNA hybridisation technology, bacterial culture, real‐time quantitative PCR or 16S rRNA gene sequencing as microbiological detection technologies.

Many similarities were observed between the results reported in the studies across the four different microbiological detection technologies. Overall, the results suggest that NSPT leads to a decrease in periodontitis‐associated subgingival microbiota at various time points after therapy. This result mirrors previous findings, which highlighted the importance of NSPT in driving shifts in the subgingival microbiota after periodontal therapy [155]. Similar time points were chosen, and 3 months after NSPT was the most frequently presented time point at which changes of mean counts in microbiota were analysed. All time points appeared to show a similar effect of NSPT on mean counts of subgingival microbiota.

Most participants were diagnosed with chronic periodontitis compared to aggressive periodontitis, and the diagnosis did not seem to influence the findings of the effect of NSPT on subgingival microbiota. This reflects the findings of other studies, which suggested that there were limited differences in the composition of subgingival microbiota between aggressive and chronic periodontitis [156]. Recently, the Consensus report of Workgroup 2 from the 2017 World Workshop on the Classification of Periodontal and Peri‐Implant Diseases and Conditions highlighted that the current evidence does not support distinguishing chronic and aggressive periodontitis as separate conditions. Instead, it suggests that distinct disease trajectories may be identified on the basis of differences in exposure and/or susceptibility [157].

In studies conducted exclusively in smokers, no statistically significant changes in mean microbial counts were found at 3 months after NSPT. Within the limitation of the small cohort of studies performed exclusively in smokers, the results indicate a poorer response in smokers regarding the decrease in mean counts of disease‐associated species. It has been previously reported that the microbial community of smoking‐associated periodontitis is less diverse and therefore distinct from that of non‐smokers [158, 159], and our results support previous findings where it has been shown that the clinical response to periodontal therapy is not as optimal in smokers [160].

When comparing studies with and without adjunctive therapies, a similar profile of changes in mean count of bacteria was observed. The subgroup meta‐analyses, which compared effect sizes in mean count reduction of disease‐associated species of studies with and without adjunctive therapies, did not show any significant differences between the two groups. In addition, a subgroup analysis of studies using CHX mouthrinse in the cohort of checkerboard DNA–DNA hybridisation technology studies was performed, which did not show an added effect in lowering mean counts of *P. gingivalis* . Clinically, however, CHX has shown an additional benefit in reducing PPD when used as a daily mouthwash after NSPT [19] and may be recommended as an adjunct in NSPT [12, 13]. A limitation of this systematic review is that the influence of systemic or local antibiotics was not assessed as an adjunct to the NSPT. A recent study has demonstrated similar microbial shifts with and without the use of systemic antibiotics. However, the addition of systemic antibiotics may have an added effect on single bacteria [161].

Furthermore, the impact of SPC provision during the clinical study was analysed for all studies with a duration of more than 3 months. SPC provision positively impacted the overall reduction of disease‐associated species. These findings were supported by a subgroup meta‐analysis which showed significantly higher effect sizes for the decrease in mean counts of *P. gingivalis* and *T. denticola*. The importance of SPC in the long‐term management of patients with a reduced but healthy periodontium following active periodontal therapy has been documented in numerous publications [162, 163, 164, 165]. This systematic review provides additional evidence that SPC can affect mean counts of subgingival microbiota as early as 6 months following NSPT and should be considered already during active periodontal therapy.

Changes of mean count of subgingival microbiota were considered as our primary outcome, because of more recent findings in periodontitis aetiology, which were described in the ‘Polymicrobial synergy and dysbiosis (PSD) model’ of periodontal disease [166]. According to this model, dysbiosis in periodontitis is caused by reductions of potentially beneficial species, combined with increases of proportions of other harmful species due to the changes in the subgingival environment. Many of the disease‐associated microbiota are therefore present in the subgingival microbiome, both in health and disease, and the key change towards disease is likely linked to their increase in relative abundance. However, frequency data regarding the detection of disease‐associated microbiota have not been examined in the present systematic review. Therefore, eradication of such species was not considered, although it may be a relevant endpoint for further investigation.

Overall, the qualitative and quantitative analysis of the effects of NSPT on subgingival microbiota across all four microbiological detection technologies suggests a decrease in disease‐associated species, such as *A. actinomycetemcomitans* , *T. denticola* , *P. gingivalis* and *T. forsythia* after NSPT. This finding reflects the current understanding of the influence of NSPT on subgingival microbiota [167].

Checkerboard DNA–DNA hybridization technology studies often present microbiological data by reporting mean values of the Socransky complexes [8] either as percentages or as pie charts. Such data were available from 12 studies [41, 46, 47, 48, 58, 59, 62, 63, 65, 66, 68, 74] and were combined to create averages across the studies. The qualitative analysis indicated a change in the relative proportions of the Socransky complexes. More specifically, there was a decrease in overall red and orange complex bacteria accompanied by an increase of *Actinomyces species* (blue) and other species (white). These changes were maintained over the investigation period of 12 months. These findings are in line with recent publications which investigated changes to the subgingival microbiota after NSPT, reporting a decrease of red complex bacteria accompanied by an increase of *Actinobacteria* which includes *Actinomyces species* [147].

Differences among the four microbial detection technologies were noticed in relation to the most frequently reported bacterial species. Although bacterial culture studies most frequently investigated the effect of NSPT on *A. actinomycetemcomitans* , DNA‐based technologies, such as real‐time quantitative PCR or checkerboard DNA–DNA hybridisation technology, reported on *P. gingivalis* or *T. forsythia* most frequently. 16S rRNA gene sequencing technology did not select species to assess the effect but instead made use of its ability to look at the subgingival microbiome by choosing appropriate analysis methods.

Also, sampling methods varied across all four microbiological detection technologies. Subgingival plaque samples were collected either with sterile paper points or with sterile curettes; both methods have been reported as leading to the same microbiological results [168]. Beyond this, samples were either collected as site‐specific samples or as pooled samples, when samples are collected from different sites and then combined. Pooled samples were used in 67% of the 16S studies, 60% of the bacterial culture, 40% of the real‐time quantitative PCR and only in 15% of the checkerboard DNA–DNA hybridisation technology studies. Checkerboard DNA–DNA hybridisation technology studies mostly analysed site‐specific samples and noticeably reported most often significant findings regarding a decrease in mean counts of *P. gingivalis* following periodontal therapy compared with the other microbiological detection technologies (66% of the studies reported significant decrease, compared with 34% in real‐time quantitative PCR studies, 50% in of 16S rRNA gene sequencing studies, and only 2% in bacterial culture studies). Site and pooled samples have been compared previously on the basis of the relative abundance of species and alpha diversity, and it was concluded that site‐specific information was in general not reflected by pooled subgingival samples [169]. Therefore, the use of pooled samples may mask site‐specific changes in subgingival microbiota following periodontal therapy and may explain some of the variability of data and the lack of statistical significance in the microbiological findings.

The influence of the study design, split‐mouth versus parallel‐arm designs, affected effect sizes, as shown in a subgroup meta‐analysis. Smaller effect sizes were noticed in the studies applying a split‐mouth design when analysing the decrease in mean counts of *A. actinomycetemcomitans* and *P. gingivalis* after NSPT. This could be an indication that a carry‐across effect is taking place in split‐mouth studies. Such carry‐across effects have been previously shown in relation to clinical outcomes [170, 171]. Overall, 30% of the studies included in this systematic review were split‐mouth design. The suggestion that carry‐across effects may take place which may influence the microbiological changes highlights the importance of the study design for future clinical studies and therefore, parallel‐arm studies are suggested as the appropriate study design.

A qualitative analysis was performed on the eight studies using 16S rRNA gene sequencing [29, 30, 36, 143, 144, 145, 146, 147] as a microbial detection technology. Studies' methodologies, such as the choice of the variable region, the number of subjects and microbiological reporting, were heterogeneous among the included studies. The choice of variable region can have an important influence on the composition of the detected microbiota. Among the studies assessed, the variable region V3–V4 was most often chosen [29, 143, 145, 146]. However, the V4 and V3–V4 regions have a higher degree of sequence conservation, making them more suitable for differentiating microbial communities up to the genus level [172, 173] and less able to distinguish between individual species. For example, species from the phylum Bacteroidetes, such as *Porphyromonas* and *Prevotella* spp. as well as *Streptococcus* spp., are not well distinguished in these regions [173, 174, 175]. Similarly, the variable region V5–V7 also has limitations in differentiating between microbiota at the species level [172]. The variable regions V1–V2 or V2–V3, on the other hand, contain greater sequence heterogeneity and have therefore greater power to distinguish between species [172]. The variable region V1–V2 has been recommended for oral samples, as these microbial communities have been well described at the species level [173] (please see Appendix S26 for information about advantage and disadvantages of commonly used variable regions).

Overall, the choice of variable region may lead to bias in the findings of 16S rRNA gene sequencing studies. In the pool of 16S rRNA gene sequencing studies, sample sizes ranged from seven to 47 participants per group. Out of those, only two studies determined the sample size on the basis of microbiological outcomes.

The changes in relative abundance of either genus or species presented few similar findings. While at the genus level, all studies reported an increase in the relative abundance of the genus *Streptococcus*, and two studies [29, 30] reported an increase in the genus *Rothia*. Previously, both genera have been associated with periodontal health [176]. In contrast, 75% of the studies [29, 30, 177] reported a decrease in the relative abundance of the genus *Porphyromonas*, a genus previously associated with periodontitis [176]. Findings at the species level were more heterogeneous than at the genera level, and a decrease in mean counts of *P. gingivalis* was the only consistent finding among the studies [29, 147, 177]. This agrees with the results of our meta‐analysis, which showed a significant reduction in *P. gingivalis* after NSPT.

Changes in alpha diversity were mostly reported using the Shannon index [29, 30, 143, 146]. Only two studies reported significant changes to the Shannon index; one study reported short‐term shifts after 6 weeks [29], and another study observed significant changes at 3 and 6 months after NSPT [144]. Periodontitis has been associated with an increase in alpha diversity [1, 11], but according to the data of the present systematic review, the effect of NSPT on alpha diversity are heterogenous. One study used expected changes to the Shannon index in saliva for sample size considerations. With a sample size of 31, they reported significant findings [29]. Most studies included in this systematic review had smaller sample sizes, and we suggest that they may have been underpowered to show significant effects to the Shannon index after NSPT. Changes in beta diversity were investigated by the Bray–Curtis dissimilarity index [30, 143], co‐occurrence networks [146] and UniFrac distance [178]. The findings were heterogeneous, and only one study [30] reported a significant change to beta diversity 3 months after NSPT.

Across all microbiological detection technologies, a lack of statistically significant differences (*p* < 0.05) was noticed. Overall, only 10%–20% of the reported changes in mean counts of subgingival microbiota after NSPT were reported as statistically significant, apart from *P. gingivalis* , which was reported in up to 50% of the studies as significantly decreased after NSPT. It is important to highlight that although the sample size of a study influences its ability to identify significant changes, only 5% of the studies included in this review performed sample size calculation on the basis of expected microbiological outcomes. Instead, 41% used clinical outcomes for their sample size calculation, and 50% did not provide information about this. Hence, the lack of sufficient statistical power could have influenced the results of the individual studies [179]. Future studies should consider the microbiological outcomes for sample size calculation. Specifically, for the 16S rRNA gene sequencing studies, various sample size calculations have been recommended, for example based on expected changes to diversity indices or mean relative abundances [180]. We propose to use expected changes to Shannon index for sample size calculation because of its wide reporting among 16S rRNA gene sequencing studies. Beyond this, the selection of the variable region should be considered when planning 16S rRNA gene sequencing studies, to enable data from different studies to be compared or pooled and analysed together, overcoming some of the limitations raised from small sample sizes.

Although this review does not focus on analysing clinical outcomes in depth (secondary outcome), studies showed that NSPT leads to a reduction in the FM‐PPD, FM‐CAL, FMBS and FMPS. These findings align with previous literature and current clinical evidence [181]. However, heterogeneity was observed in the reporting of certain clinical outcomes, in particularly large standard deviations in FMPS 3 months after NSPT. High supragingival plaque levels significantly influence the microbiological findings of subgingival plaque samples, as reported in earlier studies [182, 183]. Additionally, clinical endpoints, such as frequency of deep or residual pockets following NSPT, were rarely reported and therefore data were not extracted. Another important consideration is the clinical characteristics of the sampling site after treatment. Three of the 16S rRNA gene sequencing studies reported on PPD of the sampling sites [29, 30, 144]. In one of these studies, PPD ranged from 3.3 mm to 7.3 mm at 3 months after NSPT [29], indicating that NSPT treatment did not lead to pocket closure. Highly sensitive methods like 16S rRNA gene sequencing may not be able to detect changes to the subgingival microbiome, such as alpha diversity, if sampling is conducted at a combination of sites with varying PPD and including sites with persisting deep PPD. Therefore, we recommend single‐site sampling and clustered analysis according to PPD after treatment for future microbiome studies.

## 5 Conclusions

### 5.1 Implications for Clinical Practice

The present systematic review confirms that NSPT lowers overall mean counts of disease‐associated species found in the subgingival plaque. Furthermore, SPC provision has been demonstrated to decrease the mean counts of disease‐associated species. These changes may be maintained for up to 12 months after NSPT and may be accompanied by an increase in health‐associated species. Such shifts in the subgingival microbiome could explain potential variabilities in the clinical outcomes, such as pocket closure following NSPT. Alternatively, shallow clinical pockets may create an environment necessary for establishing a subgingival microbiome that supports periodontal health.

### 5.2 Implications for Research

A better understanding of how periodontal therapy reduces dysbiosis in the subgingival microbiome and which clinical factors, such as pocket closure following active periodontal therapy support these shifts are needed. We suggest that future microbiome studies perform sample size calculations based on microbiological outcomes (e.g., Shannon index), collect site‐specific plaque samples, and analyse microbiome data according to the clinical outcomes of study participants, such as PPD at the sampling sites post‐treatment.

## Conflicts of Interest

The authors declare no conflicts of interest.

## Supporting information


**Appendices** **S1–S26.**

## Data Availability

The raw/processed data required to reproduce the above findings can be available upon reasonable request.
