# Supplementary material for: The Effect of Non‐Surgical Periodontal Therapy on Subgingival Microbiota: A Systematic Review and Meta‐Analysis
Source: J Periodontal Res. 2025 May 9;60(10):963–93. doi: 10.1111/jre.13409 (PMC12640219; doi:10.1111/jre.13409)
Supplement: Supplementary file 1 — Appendices S1–S26. [file JRE-60-963-s001.docx]

**Supplementary Material**

**Appendix S1.** PRISMA for study selection process


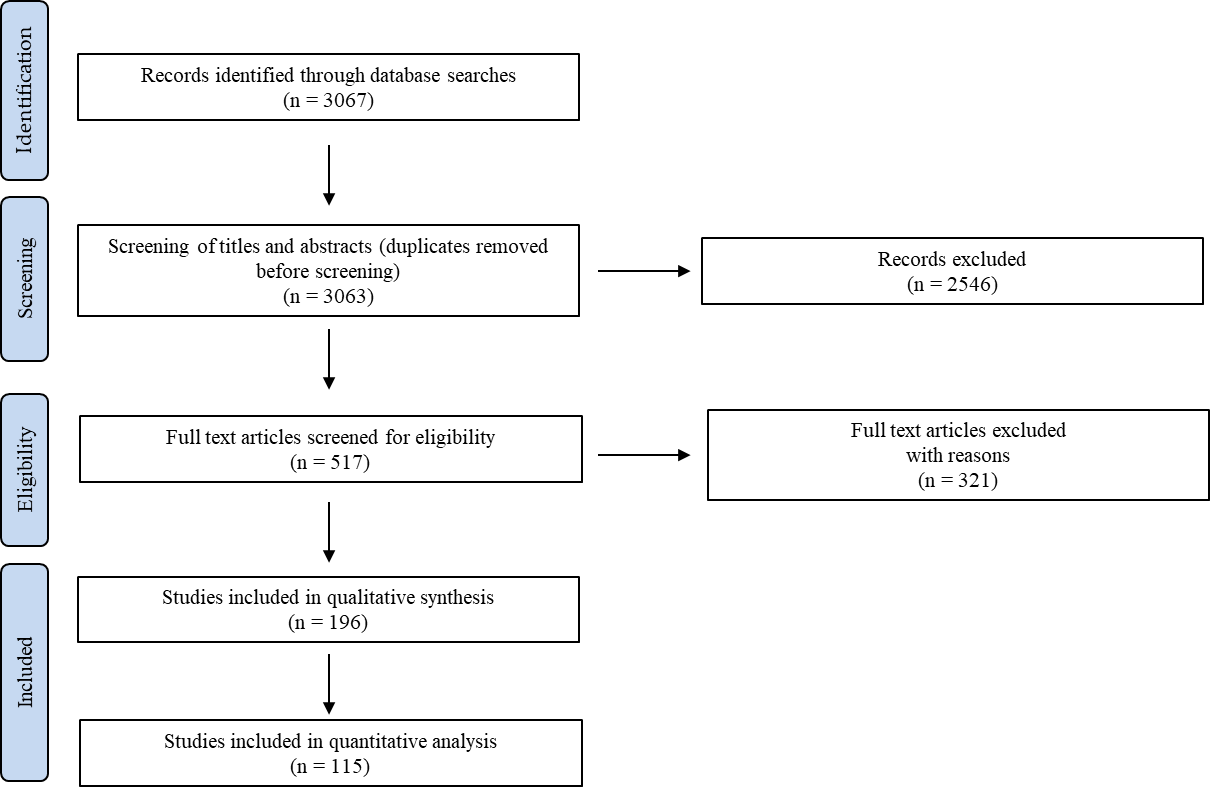


**Appendix S2.** Reason for exclusion from systematic review after full text screening

| **Reason** | **Number** |
| --- | --- |
| Use of systemic or local antibiotic within 3 months before start of the study or during the study or not part of the exclusion criteria | 58 |
| Inclusion and exclusion criteria not sufficiently defined | 36 |
| Time-point for microbiological samples collection less than 6 weeks after NSPT | 30 |
| Patients suffering from systemic diseases | 30 |
| Patients did not receive NSPT | 6 |
| Patients in supportive periodontal care | 22 |
| Patients did receive NSPT before baseline data collection | 21 |
| Patients receive periodontal surgeries during study | 22 |
| No full text available | 19 |
| Language | 18 |
| No clinical data reported | 12 |
| Age of study subjects | 12 |
| No microbiological data reported | 6 |
| Other | 18 |

**Appendix S3.** Characteristics of the included studies. (A) Study design, (B) split-mouth versus whole-mouth study design, (C) smoking status of study participants, and (D) microbiological detection technique

**Appendix S4.** Characteristics of all studies included in the first-stage data extraction.

| Authors, year | Study design | Split mouth | Country | Microbiologic detection technique | Smoking |
| --- | --- | --- | --- | --- | --- |
| Abreu et al. 2019 | CSS | No | Brazil | PCR | Both (case-control) |
| Ali et al. 1992 | CT | Yes | Norway | Culture | No information |
| Alzoman et al. 2016 | RCT | No | Saudi Arabia | PCR | Non-smokers |
| Ambrosini et al. 2005 | RCT | Yes | France | PCR | Both |
| Annaji et al. 2016 | RCT | Yes | India | Culture | Non-smokers |
| Apatzidou et al. 2004 | RCT | No | UK | PCR | No information |
| Apatzidou et al. 2005 | RCT | No | UK | PCR | Both (case-control) |
| Apatzidou et al. 2014 | RCT | No | Greece | Checkerboard | Both |
| Ardila et al. 2015 | RCT | No | Colombia | Culture | Non-smokers |
| Ardila et al. 2017 | RCT | No | Colombia | Culture | Non-smokers |
| Beikler et al. 2005 | Long. | No | Germany | PCR | Both |
| Belstrom et al. 2018 | Long. | No | Germany | 16S | Both |
| Bergamaschi et al. 2016 | RCT | No | Brazil | qPCR | Smokers |
| Berglundh et al. 1998 | RCT | Yes | Sweden | Culture | No information |
| Bhatia et al. 2014 | RCT | Yes | India | Culture | Non-smokers |
| Bizzarro et al. 2016 | RCT | No | Netherlands | 16S | Both |
| Bizzarro et al. 2016_2 | RCT | No | Netherlands | Culture | Both |
| Boia et al. 2019 | RCT | No | Romania | PCR | Both |
| Bollen et al. 1996 | RCT | No | Belgium | Microscope, culture | No information |
| Bollen et al. 1998 | RCT | No | Belgium | Microscope, culture | Both |
| Borekci et al. 2019 | RCT | No | Turkey | Checkerboard | Non-smokers |
| Bozoglan et al. 2017 | CSS | No | Turkey | Checkerboard | Non-smokers |
| Brochut et al. 2005 | Long. | No | Swiss | Checkerboard | Non-smokers |
| Buzini et al. 2014 | CCS | No | Malaysia | qPCR | Non-smokers |
| Calderini et al. 2013 | RCT | Yes | Italy | PCR | Non-smokers |
| Camargo et al. 2016 | CSS | Yes | Brazil | PCR | Both (case-control) |
| Carvalho et al. 2005 | RCT | No | Brazil | Checkerboard | No information |
| ChinQuee et al. 1988 | RCT | No | Canada | Microscope | No information |
| Chitsazi et al. 2014 | RCT | Yes | Iran | PCR | Non-smokers |
| Christgau et al. 1998 | Long. | No | Germany | Enzymes | Both |
| Christgau et al. 2006 | RCT | Yes | Germany | Checkerboard | Both |
| Christgau et al. 2007 | RCT | Yes | Germany | Checkerboard | Both |
| Christodoulides et al. 2008 | RCT | No | Netherlands | PCR | Both |
| Cirino et al. 2019 | RCT | Yes | Brazil | Checkerboard | No information |
| Colombo et al. 2005 | Long. | No | Brazil | Checkerboard | Both |
| Cortelli et al. 2008 | RCT | No | Brazil | PCR | No information |
| Cortelli et al. 2015 | RCT | No | Brazil | qPCR | Both |
| Cosgarea et al. 2019 | CSS | No | Romania | qPCR | Both |
| Cosyn et al. 2007 | RCT | No | Belgium | PCR | Non-smokers |
| Cugini et al. 2000 | Long. | No | USA | Checkerboard | Both |
| D'Ercole et al. 2006 | CT | Yes | Italy | PCR | Non-smokers |
| da Cruz et al. 2008 | CSS | No | Brazil | PCR | Non-smokers |
| Darby et al. 2001 | Long. | No | UK | PCR | Both |
| Darby et al. 2005 | CSS | No | UK | PCR | Both (case-control) |
| Del Peloso Ribeiro et al. 2008 | RCT | No | Brazil | qPCR | Non-smokers |
| de Melo Soares et al. 2019 | RCT | Yes | Brazil | Checkerboard | Smokers |
| de Micheli et al. 2011 | RCT | Yes | Brazil | Culture | Non-smokers |
| Derdilopoulou et al. 2007 | RCT | Yes | Germany | PCR | No information |
| De Soete et al. 2005 | RCT | No | Belgium | Culture | Both |
| Dhaliwal et al. 2017 | RCT | No | India | Culture | No information |
| Dominguez et al. 2010 | RCT | No | Spain | Culture | Non-smokers |
| Do Vale et al. 2016 | RCT | No | Brazil | qPCR | Non-smokers |
| Eckles et al. 1990 | RCT | Yes | USA | Microscope | No information |
| Ehmke et al. 1999 | RCT | No | Germany | PCR | No information |
| Eick et al. 2013 | RCT | No | Germany | qPCR | Non-smokers |
| Eick et al. 2017 | Long. | No | Germany | PCR, qPCR | Both |
| Euzebio Alves et al. 2013 | RCT | Yes | Brazil | Culture | Non-smokers |
| Everett et al. 2017 | RCT | Yes | USA | PCR | Non-smokers |
| Faveri et al. 2006 | RCT | No | Brazil | BANA | Non-smokers |
| Feres et al. 2009 | RCT | No | Brazil | Checkerboard | Non-smokers |
| Feres et al. 2015 | CSS | No | Brazil | Checkerboard | Both (case-control) |
| Flemmig et al. 2011 | RCT | No | Germany | PCR | Both |
| Fonseca et al. 2015 | RCT | No | Brazil | qPCR | Both |
| Forgas et al. 1987 | Long. | No | USA | Microscope | No information |
| Gibson et al. 1994 | RCT | Yes | UK | Microscope | No information |
| Gomez et al. 2011 | RCT | No | Spain | Culture | Non-smokers |
| Gomi et al. 2007 | RCT | No | Japan | PCR | Non-smokers |
| Gottumukkala et al. 2013 | RCT | No | India | BANA | Non-smokers |
| Greenwell et al. 1984 | CT | No | USA | Microscope | No information |
| Grisi et al. 2002 | RCT | No | Brazil | BANA | Non-smokers |
| Grzech-Lesniak et al. 2018 | RCT | No | Poland | qPCR | Non-smokers |
| Guentsch et al. 2008 | RCT | No | Germany | qPCR | Both |
| Guerrero et al. 2014 | RCT | No | UK | PCR | Both |
| Haffajee et al. 1997 | Long. | No | USA | Checkerboard | Both |
| Haffajee et al. 1997_2 | Long. | No | USA | Checkerboard | Both |
| Haffajee et al. 2008 | RCT | No | USA | Checkerboard | Both |
| Hagenfeld et al. 2018 | Long. | No | Germany | 16S | No information |
| Han et al. 2012 | RCT | No | Turkey | qPCR | Both |
| Hayakumo et al. 2013 | RCT | No | Japan | qPCR | Non-smokers |
| Heller et al. 2011 | RCT | No | Brazil | Checkerboard | Both |
| Husejnagic et al. 2019 | RCT | Yes | Austria | PCR | Both |
| Ioannou et al. 2009 | RCT | No | Greece | Checkerboard | Both |
| Ioannou et al. 2011 | RCT | No | Greece | Checkerboard | Both |
| Isola et al. 2018 | RCT | Yes | Italy | Checkerboard | Non-smokers |
| Iziodoro et al. 2023 | Long. | No | Portugal | 16S | Both |
| Jeong et al. 1994 | RCT | No | Korea | Microscope | No information |
| Jervoe-Storm et al. 2007_2 | RCT | No | Germany | qPCR | Both |
| Jones et al. 1994 | RCT | No | USA | Checkerboard | No information |
| Knofler et al. 2011 | RCT | No | Germany | qPCR | No information |
| Koshy et al. 2005 | RCT | No | Japan | PCR | Non-smokers |
| Leonhardt et al. 2007 | RCT | Yes | Italy/ Sweden | Checkerboard | Non-smokers |
| Lie et al. 1998 | RCT | Yes | Norway | Enzymes | No information |
| Lindhe et al. 1983 | RCT | Yes | Sweden | Microscope | No information |
| Lindhe et al. 1985 | RCT | Yes | Sweden | Microscope | No information |
| Lira et al. 2013 | RCT | Yes | Brazil | qPCR | Non-smokers |
| Liu et al. 2013 | Long. | No | China | qPCR | Non-smokers |
| Liu et al. 2018 | Long. | No | China | 16S | Non-smokers |
| Lombardo et al. 2015 | RCT | Yes | Italy | Culture | Non-smokers |
| Lopes et al. 2010 | RCT | Yes | Brazil | PCR | Non-smokers |
| Lopez et al. 2006 | RCT | No | Chile | Checkerboard | Both |
| Luchesi et al. 2013 | RCT | No | Brazil | qPCR | Non-smokers |
| Lu et al. 2021 | RCT | No | China | 16S | Non-smokers |
| Mahendra et al. 2017 | RCT | No | India | PCR | Non-smokers |
| Machion et al. 2004 | RCT | No | Brazil | PCR | Smokers |
| Malali et al. 2012 | RCT | No | Turkey | Microscope | Non-smokers |
| Manikandan et al. 2016 | RCT | Yes | India | Culture | No information |
| Martande et al. 2016 | RCT | No | India | Culture | Non-smokers |
| Martelli et al. 2016 | Long. | No | Germany | 16S | No information |
| Matarese et al. 2017 | RCT | Yes | Italy | Checkerboard | Non-smokers |
| Matarazzo et al. 2008 | RCT | No | Brazil | Checkerboard | Smokers |
| Mdala et al. 2013 | RCT | No | Norway | Checkerboard | Both |
| Mestnik et al. 2010 | RCT | No | Brazil | Checkerboard | Non-smokers |
| Mizrak et al. 2006 | RCT | No | Turkey | Microscope | Non-smokers |
| Moeintaghavi et al. 2007 | RCT | No | Iran | Culture | Non-smokers |
| Morales et al. 2018 | RCT | No | Chile | Culture | Both |
| MShaddox et al. 2007 | RCT | No | USA | PCR | Smokers |
| Nie et al. 2024 | RCT | No | USA | 16S | Non-smokers |
| Novaes et al. 2012 | RCT | Yes | Brazil | Checkerboard | Non-smokers |
| Oosterwaal et al. 1991 | RCT | Yes | Netherlands | Microscope | No information |
| Ower et al. 1995 | RCT | Yes | UK | BANA | No information |
| Paolantonio et al. 2008 | RCT | Yes | Italy | PCR | Non-smokers |
| Paolantonio et al. 2009 | RCT | Yes | Italy | PCR | Non-smokers |
| Park et al. 2018 | RCT | Yes | Korea | qPCR | No information |
| Patel et al. 2012 | RCT | Yes | India - UK | PCR | Non-Smoker |
| Penala et al. 2016 | RCT | No | Saudi Arabia  India | BANA | Non-smokers |
| Peralta et al. 2020 | CSS | No | Brazil | qPCR | Both |
| Perayil et al. 2016 | CT | Yes | India | PCR | Non-smokers |
| Pereira et al. 2012 | CT | No | Brazil | PCR | Non-smokers |
| Perrella et al. 2016 | RCT | No | Brazil | Checkerboard | Non-smokers |
| Polansky et al. 2009 | RCT | No | Austria | PCR | Both |
| Pradeep et al. 2008 | RCT | No | India | Microscope | No information |
| Pradeep et al. 2011 | RCT | No | India | PCR | Non-smokers |
| Pradeep et al. 2013 | RCT | No | India | PCR | Non-smokers |
| Pradeep et al. 2014 | RCT | No | India | Culture | No information |
| Pradeep et al. 2015 | RCT | No | India | PCR | Non-smokers |
| Preber et al. 1995 | CSS | No | Sweden | Culture | Both (case-control) |
| Priyanka et al. 2015 | RCT | No | India | PCR | Non-smokers |
| Pulikkotil et al. 2016 | RCT | Yes | Malaysia | qPCR | Non-smokers |
| Querido et al. 2004 | RCT | No | Brazil | PCR | No information |
| Quirynen et al. 1995 | RCT | No | Belgium | Microscope, culture | Both |
| Quirynen et al. 1999 | RCT | No | Belgium | Microscope, culture | Both |
| Quirynen et al. 2000 | RCT | No | Belgium | Microscope, culture | Both |
| Raj et al. 2016 | RCT | No | India | PCR | Non-smokers |
| Ramiro et al. 2018 | RCT | No | Brazil | qPCR | Non-smokers |
| Rassameemasmaung et al. 2008 | RCT | No | Thailand | Culture | Non-smoker |
| Ready et al. 2008 | Long. | No | UK/ Switzerland | PCR | Both |
| Renvert et al. 1992 | Long. | No | Ireland/ Sweden | Microscope | No information |
| Renvert et al. 1997 | RCT | Yes | Sweden | Enzyme | No information |
| Renvert et al. 1998 | CSS | No | Sweden | Culture | Both (case-control) |
| Roman-Torres et al. 2018 | RCT | No | Brazil | Culture | Non-smokers |
| Rooney et al. 2002 | RCT | No | UK | Culture | No information |
| Rosalem et al. 2011 | CSS | No | Sweden/ Brazil | Checkerboard | Both |
| Rosling et al. 1983 | RCT | Yes | USA | Microscope | No information |
| Saglam et al. 2013 | RCT | No | Turkey | qPCR | Non-smokers |
| Saglam et al. 2017 | RCT | Yes | Turkey | qPCR | Non-smokers |
| Sakellari et al. 2010 | RCT | No | Greece | Checkerboard | Both |
| Sampaio et al. 2011 | RCT | No | Brazil | Checkerboard | Both |
| Sanz-Sanchez et al. 2016 | RCT | No | Spain | Culture | Both |
| Sbordone et al. 1990 | Long. | No | Italy/USA | Microscope | No information |
| Schwarz et al. 2001 | RCT | Yes | Germany | Microscope | No information |
| Schwarz et al. 2003_1 | RCT | Yes | Germany | Microscope | No information |
| Schwarz et al. 2003_2 | RCT | Yes | Germany | Microscope | No information |
| Sefton et al. 1996 | RCT | No | UK | Culture | No information |
| Shiloah et al. 1997 | RCT | Yes | USA | Checkerboard | Both |
| Shiloah et al. 1998 | RCT | Yes | USA | Checkerboard | Both |
| Silva et al. 2011 | RCT | No | Brazil | Checkerboard | Non-smokers |
| Silva-Boghossian et al. 2014 | CSS | No | Brazil | Checkerboard | Non-smokers |
| Silva-Senem et al. 2013 | RCT | No | Brazil | Checkerboard | Both |
| Sindhura et al. 2017 | RCT | Yes | India | PCR | No information |
| Soares et al. 2014 | RCT | No | Brazil | Checkerboard | Non-smokers |
| Soeroso et al. 2017 | RCT | No | Indonesia | qPCR | Non-smokers |
| Spooner et al. 2016 | Long. | No | USA | qPCR | No information |
| Sreedhar et al. 2015 | RCT | Yes | India | Culture | Non-smokers |
| Suchetha et al. 2013 | RCT | No | India | Culture | Non-smokers |
| Suryaprasanna et al. 2018 | RCT | No | India | Culture | Non-smokers |
| Swierkot et al. 2009 | RCT | No | Germany | qPCR | Both |
| Tabenski et al. 2017 | RCT | No | Germany | Checkerboard | Both |
| Talebi et al. 2016 | RCT | Yes | Iran | PCR | Non-smokers |
| Tanaka et al. 2015 | RCT | No | Brazil | qPCR | No information |
| Tekce et al. 2015 | RCT | No | Turkey | Culture | Non-smokers |
| Teughels et al. 2013 | RCT | No | Turkey, Belgium | qPCR | Non-smokers |
| Theodoro et al. 2012 | RCT | Yes | Brazil | PCR | Non-smokers |
| Theodoro et al. 2018 | RCT | Yes | Brazil | qPCR | Smokers |
| Timmerman et al. 1996 | RCT | No | Netherlands | Checkerboard | No information |
| Uraz et al. 2019 | RCT | Yes | Turkey | qPCR | Non-smokers |
| Winkel et al. 1999 | RCT | No | Netherlands | Microscope | Both |
| Winkel et al. 2001 | RCT | No | Netherlands | Culture | Both |
| Xajigeorgiou et al. 2006 | RCT | No | Greece | Checkerboard | Both |
| Yashima et al. 2009 | RCT | No | Japan | PCR | Non-smokers |
| Yek et al. 2010 | RCT | No | USA/ Turkey | PCR | Non-smokers |
| Yeom et al. 1997 | RCT | Yes | Korea | Microscope | No information |
| Yilmaz et al. 2012 | RCT | Yes | Turkey | Culture | No information |
| Yilmaz et al. 2013 | RCT | Yes | Turkey | Culture | Non-smokers |
| ZenginCelik et al. 2019 | RCT | No | Turkey | qPCR | Non-smokers |
| Zijnge et al. 2010 | RCT | No | Netherlands | PCR | Non-smokers |
| RCT: randomized controlled trial, CT: controlled trial, Long: longitudinal study, CSS: case-control study, PCR: polymerase chain reaction, qPCR: quantitative polymerase chain reaction, 16S: 16S gene sequencing  Both: smoker and non-smoker | | | | | |

**Appendix S5.** Reference list of all identified studies meeting the inclusion and exclusion criteria, in alphabetic order.

1. Abreu, M. G. L., Kawamoto, D., Mayer, M. P. A., Pascoal, V. D. B., Caiaffa, K. S., Zuza, E. P., Duque, C., & Camargo, G. (2019). Frequency of Porphyromonas gingivalis fimA in smokers and nonsmokers after periodontal therapy. J Appl Oral Sci, 27, e20180205. https://doi.org/10.1590/1678-7757-2018-0205
2. Ali, R. W., Lie, T., & Skaug, N. (1992). Early effects of periodontal therapy on the detection frequency of four putative periodontal pathogens in adults. J Periodontol, 63(6), 540-547. https://doi.org/10.1902/jop.1992.63.6.540
3. Alzoman, H. A., & Diab, H. M. (2016). Effect of gallium aluminium arsenide diode laser therapy on Porphyromonas gingivalis in chronic periodontitis: a randomized controlled trial. Int J Dent Hyg, 14(4), 261-266. https://doi.org/10.1111/idh.12169
4. Ambrosini, P., Miller, N., Briancon, S., Gallina, S., & Penaud, J. (2005). Clinical and microbiological evaluation of the effectiveness of the Nd:Yap laser for the initial treatment of adult periodontitis. A randomized controlled study. J Clin Periodontol, 32(6), 670-676. https://doi.org/10.1111/j.1600-051X.2005.00738.x
5. Annaji, S., Sarkar, I., Rajan, P., Pai, J., Malagi, S., Bharmappa, R., & Kamath, V. (2016). Efficacy of Photodynamic Therapy and Lasers as an Adjunct to Scaling and Root Planing in the Treatment of Aggressive Periodontitis - A Clinical and Microbiologic Short-Term Study. J Clin Diagn Res, 10(2), ZC08-12. https://doi.org/10.7860/JCDR/2016/13844.7165
6. Apatzidou, D. A., Riggio, M. P., & Kinane, D. F. (2004). Quadrant root planing versus same-day full-mouth root planing. II. Microbiological findings. J Clin Periodontol, 31(2), 141-148. https://doi.org/10.1111/j.0303-6979.2004.00462.x
7. Apatzidou, D. A., Riggio, M. P., & Kinane, D. F. (2005). Impact of smoking on the clinical, microbiological and immunological parameters of adult patients with periodontitis. J Clin Periodontol, 32(9), 973-983. https://doi.org/10.1111/j.1600-051X.2005.00788.x
8. Apatzidou, D. A., Zygogianni, P., Sakellari, D., & Konstantinidis, A. (2014). Oral hygiene reinforcement in the simplified periodontal treatment of 1 hour. J Clin Periodontol, 41(2), 149-156. https://doi.org/10.1111/jcpe.12200
9. Ardila, C. M., & Guzman, I. C. (2017). Benefits of adjunctive moxifloxacin in generalized aggressive periodontitis: a subgroup analyses in Aggregatibacter actinomycetemcomitans-positive/negative patients from a clinical trial. J Investig Clin Dent, 8(2). https://doi.org/10.1111/jicd.12197
10. Ardila, C. M., Martelo-Cadavid, J. F., Boderth-Acosta, G., Ariza-Garces, A. A., & Guzman, I. C. (2015). Adjunctive moxifloxacin in the treatment of generalized aggressive periodontitis patients: clinical and microbiological results of a randomized, triple-blind and placebo-controlled clinical trial. J Clin Periodontol, 42(2), 160-168. https://doi.org/10.1111/jcpe.12345
11. Beikler, T., Peters, U., Prior, K., Ehmke, B., & Flemmig, T. F. (2005). Sequence variations in rgpA and rgpB of Porphyromonas gingivalis in periodontitis. J Periodontal Res, 40(3), 193-198. https://doi.org/10.1111/j.1600-0765.2005.00783.x
12. Belstrøm, D., Grande, M. A., Sembler-Møller, M. L., Kirkby, N., Cotton, S. L., Paster, B. J., & Holmstrup, P. (2018). Influence of periodontal treatment on subgingival and salivary microbiotas. J Periodontol, 89(5), 531-539. https://doi.org/10.1002/JPER.17-0377
13. Bergamaschi, C. C., Santamaria, M. P., Berto, L. A., Cogo-Muller, K., Motta, R. H., Salum, E. A., Nociti Junior, F. H., Goodson, J. M., & Groppo, F. C. (2016). Full mouth periodontal debridement with or without adjunctive metronidazole gel in smoking patients with chronic periodontitis: A pilot study. J Periodontal Res, 51(1), 50-59. https://doi.org/10.1111/jre.12278
14. Berglundh, T., Krok, L., Liljenberg, B., Westfelt, E., Serino, G., & Lindhe, J. (1998). The use of metronidazole and amoxicillin in the treatment of advanced periodontal disease. A prospective, controlled clinical trial. J Clin Periodontol, 25(5), 354-362. https://doi.org/10.1111/j.1600-051x.1998.tb02455.x
15. Bhatia, M., Urolagin, S. S., Pentyala, K. B., Urolagin, S. B., K, B. M., & Bhoi, S. (2014). Novel therapeutic approach for the treatment of periodontitis by curcumin. J Clin Diagn Res, 8(12), ZC65-69. https://doi.org/10.7860/JCDR/2014/8231.5343
16. Bizzarro, S., Laine, M. L., Buijs, M. J., Brandt, B. W., Crielaard, W., Loos, B. G., & Zaura, E. (2016). Microbial profiles at baseline and not the use of antibiotics determine the clinical outcome of the treatment of chronic periodontitis. Sci Rep, 6, 20205. https://doi.org/10.1038/srep20205
17. Bizzarro, S., Van der Velden, U., & Loos, B. G. (2016). Local disinfection with sodium hypochlorite as adjunct to basic periodontal therapy: a randomized controlled trial. J Clin Periodontol, 43(9), 778-788. https://doi.org/10.1111/jcpe.12578
18. Boia, S., Boariu, M., Baderca, F., Rusu, D., Muntean, D., Horhat, F., Boia, E. R., Borza, C., Anghel, A., & Stratul, S. I. (2019). Clinical, microbiological and oxidative stress evaluation of periodontitis patients treated with two regimens of systemic antibiotics, adjunctive to non-surgical therapy: A placebo-controlled randomized clinical trial. Exp Ther Med, 18(6), 5001-5015. https://doi.org/10.3892/etm.2019.7856
19. Bollen, C. M., Mongardini, C., Papaioannou, W., Van Steenberghe, D., & Quirynen, M. (1998). The effect of a one-stage full-mouth disinfection on different intra-oral niches. Clinical and microbiological observations. J Clin Periodontol, 25(1), 56-66. https://doi.org/10.1111/j.1600-051x.1998.tb02364.x
20. Bollen, C. M., Vandekerckhove, B. N., Papaioannou, W., Van Eldere, J., & Quirynen, M. (1996). Full- versus partial-mouth disinfection in the treatment of periodontal infections. A pilot study: long-term microbiological observations. J Clin Periodontol, 23(10), 960-970. https://doi.org/10.1111/j.1600-051x.1996.tb00519.x
21. Borekci, T., Meseli, S. E., Noyan, U., Kuru, B. E., & Kuru, L. (2019). Efficacy of adjunctive photodynamic therapy in the treatment of generalized aggressive periodontitis: A randomized controlled clinical trial. Lasers Surg Med, 51(2), 167-175. https://doi.org/10.1002/lsm.23010
22. Bozoglan, A., Ertugrul, A. S., Taspinar, M., & Yuzbasioglu, B. (2017). Determining the relationship between atherosclerosis and periodontopathogenic microorganisms in chronic periodontitis patients. Acta Odontol Scand, 75(4), 233-242. https://doi.org/10.1080/00016357.2017.1280739
23. Brochut, P. F., Marin, I., Baehni, P., & Mombelli, A. (2005). Predictive value of clinical and microbiological parameters for the treatment outcome of scaling and root planing. J Clin Periodontol, 32(7), 695-701. https://doi.org/10.1111/j.1600-051X.2005.00730.x
24. Buzinin, S. M., Alabsi, A. M., Tan, A. T., Vincent-Chong, V. K., & Swaminathan, D. (2014). Effects of nonsurgical periodontal therapy on clinical response, microbiological profile, and glycemic control in Malaysian subjects with type 1 diabetes. ScientificWorldJournal, 2014, 232535. https://doi.org/10.1155/2014/232535
25. Calderini, A., Pantaleo, G., Rossi, A., Gazzolo, D., & Polizzi, E. (2013). Adjunctive effect of chlorhexidine antiseptics in mechanical periodontal treatment: first results of a preliminary case series. Int J Dent Hyg, 11(3), 180-185. https://doi.org/10.1111/idh.12009
26. Camargo, G. A., Abreu, M. G., Cordeiro Rdos, S., Wenderoscky Lde, F., & Duque, C. (2016). Prevalence of periodontopathogens and Candida spp. in smokers after nonsurgical periodontal therapy - a pilot study. Braz Oral Res, 30(1), e92. https://doi.org/10.1590/1807-3107BOR-2016.vol30.0092
27. Carvalho, L. H., D'Avila, G. B., Leao, A., Goncalves, C., Haffajee, A. D., Socransky, S. S., & Feres, M. (2005). Scaling and root planing, systemic metronidazole and professional plaque removal in the treatment of chronic periodontitis in a Brazilian population II--microbiological results. J Clin Periodontol, 32(4), 406-411. https://doi.org/10.1111/j.1600-051X.2005.00720.x
28. Chin Quee, T., Al-Joburi, W., Lautar-Lemay, C., Chan, E. C., Iugovaz, I., Bourgouin, J., & Delorme, F. (1988). Comparison of spiramycin and tetracycline used adjunctively in the treatment of advanced chronic periodontitis. J Antimicrob Chemother, 22 Suppl B, 171-177. https://doi.org/10.1093/jac/22.supplement_b.171
29. Chitsazi, M. T., Shirmohammadi, A., Pourabbas, R., Abolfazli, N., Farhoudi, I., Daghigh Azar, B., & Farhadi, F. (2014). Clinical and Microbiological Effects of Photodynamic Therapy Associated with Non-surgical Treatment in Aggressive Periodontitis. J Dent Res Dent Clin Dent Prospects, 8(3), 153-159. https://doi.org/10.5681/joddd.2014.028
30. Christgau, M., Manner, T., Beuer, S., Hiller, K. A., & Schmalz, G. (2006). Periodontal healing after non-surgical therapy with a modified sonic scaler: a controlled clinical trial. J Clin Periodontol, 33(10), 749-758. https://doi.org/10.1111/j.1600-051X.2006.00981.x
31. Christgau, M., Manner, T., Beuer, S., Hiller, K. A., & Schmalz, G. (2007). Periodontal healing after non-surgical therapy with a new ultrasonic device: a randomized controlled clinical trial. J Clin Periodontol, 34(2), 137-147. https://doi.org/10.1111/j.1600-051X.2006.01031.x
32. Christgau, M., Palitzsch, K. D., Schmalz, G., Kreiner, U., & Frenzel, S. (1998). Healing response to non-surgical periodontal therapy in patients with diabetes mellitus: clinical, microbiological, and immunologic results. J Clin Periodontol, 25(2), 112-124. https://doi.org/10.1111/j.1600-051x.1998.tb02417.x
33. Christodoulides, N., Nikolidakis, D., Chondros, P., Becker, J., Schwarz, F., Rossler, R., & Sculean, A. (2008). Photodynamic therapy as an adjunct to non-surgical periodontal treatment: a randomized, controlled clinical trial. J Periodontol, 79(9), 1638-1644. https://doi.org/10.1902/jop.2008.070652
34. Cirino, C., Vale, H. F. D., Casati, M. Z., Sallum, E. A., Casarin, R. C. V., & Sallum, A. W. (2019). Clinical and Microbiological Evaluation of Surgical and Nonsurgical Treatment of Aggressive Periodontitis. Braz Dent J, 30(6), 577-586. https://doi.org/10.1590/0103-6440201902930
35. Colombo, A. P., Teles, R. P., Torres, M. C., Rosalem, W., Mendes, M. C., Souto, R. M., & Uzeda, M. (2005). Effects of non-surgical mechanical therapy on the subgingival microbiota of Brazilians with untreated chronic periodontitis: 9-month results. J Periodontol, 76(5), 778-784. https://doi.org/10.1902/jop.2005.76.5.778
36. Cortelli, J. R., Aquino, D. R., Cortelli, S. C., Carvalho-Filho, J., Roman-Torres, C. V., & Costa, F. O. (2008). A double-blind randomized clinical trial of subgingival minocycline for chronic periodontitis. J Oral Sci, 50(3), 259-265. https://doi.org/10.2334/josnusd.50.259
37. Cortelli, S. C., Costa, F. O., Rodrigues, E., Cota, L. O., & Cortelli, J. R. (2015). Periodontal Therapy Effects on Nitrite Related to Oral Bacteria: A 6-Month Randomized Clinical Trial. J Periodontol, 86(8), 984-994. https://doi.org/10.1902/jop.2015.140678
38. Cosgarea, R., Tristiu, R., Dumitru, R. B., Arweiler, N. B., Rednic, S., Sirbu, C. I., Lascu, L., Sculean, A., & Eick, S. (2019). Effects of non-surgical periodontal therapy on periodontal laboratory and clinical data as well as on disease activity in patients with rheumatoid arthritis. Clin Oral Investig, 23(1), 141-151. https://doi.org/10.1007/s00784-018-2420-3
39. Cosyn, J., De Bruyn, H., & Sabzevar, M. M. (2007). [Subgingival application of chlorhexidine in the treatment of periodontitis]. Rev Belge Med Dent (1984), 62(4), 176-182. https://www.ncbi.nlm.nih.gov/pubmed/18506992 (Application sous-gingivale de chlorhexidine dans le traitement de la parodontite.)
40. Cugini, M. A., Haffajee, A. D., Smith, C., Kent, R. L., Jr., & Socransky, S. S. (2000). The effect of scaling and root planing on the clinical and microbiological parameters of periodontal diseases: 12-month results. J Clin Periodontol, 27(1), 30-36. https://doi.org/10.1034/j.1600-051x.2000.027001030.x
41. D'Ercole, S., Piccolomini, R., Capaldo, G., Catamo, G., Perinetti, G., & Guida, L. (2006). Effectiveness of ultrasonic instruments in the therapy of severe periodontitis: a comparative clinical-microbiological assessment with curettes. New Microbiol, 29(2), 101-110. https://www.ncbi.nlm.nih.gov/pubmed/16841550
42. da Cruz, G. A., de Toledo, S., Sallum, E. A., Sallum, A. W., Ambrosano, G. M., de Cassia Orlandi Sardi, J., da Cruz, S. E., & Goncalves, R. B. (2008). Clinical and laboratory evaluations of non-surgical periodontal treatment in subjects with diabetes mellitus. J Periodontol, 79(7), 1150-1157. https://doi.org/10.1902/jop.2008.070503
43. Darby, I. B., Hodge, P. J., Riggio, M. P., & Kinane, D. F. (2005). Clinical and microbiological effect of scaling and root planing in smoker and non-smoker chronic and aggressive periodontitis patients. J Clin Periodontol, 32(2), 200-206. https://doi.org/10.1111/j.1600-051X.2005.00644.x
44. Darby, I. B., Mooney, J., & Kinane, D. F. (2001). Changes in subgingival microflora and humoral immune response following periodontal therapy. J Clin Periodontol, 28(8), 796-805. <https://doi.org/10.1034/j.1600-051x.2001.280812.x>
45. Del Peloso Ribeiro, E., Bittencourt, S., Sallum, E. A., Nociti, F. H., Jr., Goncalves, R. B., & Casati, M. Z. (2008). Periodontal debridement as a therapeutic approach for severe chronic periodontitis: a clinical, microbiological and immunological study. J Clin Periodontol, 35(9), 789-798. https://doi.org/10.1111/j.1600-051X.2008.01292.x
46. de Melo Soares, M. S., D'Almeida Borges, C., de Mendonça Invernici, M., Frantz, F. G., de Figueiredo, L. C., de Souza, S. L. S., Taba, M., Messora, M. R., & Novaes, A. B. (2019). Antimicrobial photodynamic therapy as adjunct to non-surgical periodontal treatment in smokers: a randomized clinical trial. Clin Oral Investig, 23(8), 3173-3182. https://doi.org/10.1007/s00784-018-2740-3
47. De Micheli, G., de Andrade, A. K., Alves, V. T., Seto, M., Pannuti, C. M., & Cai, S. (2011). Efficacy of high intensity diode laser as an adjunct to non-surgical periodontal treatment: a randomized controlled trial. Lasers Med Sci, 26(1), 43-48. https://doi.org/10.1007/s10103-009-0753-5
48. Derdilopoulou, F. V., Nonhoff, J., Neumann, K., & Kielbassa, A. M. (2007). Microbiological findings after periodontal therapy using curettes, Er:YAG laser, sonic, and ultrasonic scalers. J Clin Periodontol, 34(7), 588-598. https://doi.org/10.1111/j.1600-051X.2007.01093.x
49. De Soete, M., Dekeyser, C., Pauwels, M., Teughels, W., van Steenberghe, D., & Quirynen, M. (2005). Increase in cariogenic bacteria after initial periodontal therapy. J Dent Res, 84(1), 48-53. https://doi.org/10.1177/154405910508400108
50. Dhaliwal, P. K., Grover, V., Malhotra, R., & Kapoor, A. (2017). Clinical and Microbiological Investigation of the Effects of Probiotics Combined with Scaling and Root Planing in the Management of Chronic Periodontitis: A Randomized, Controlled Study. J Int Acad Periodontol, 19(3), 101-108. https://www.ncbi.nlm.nih.gov/pubmed/31473697
51. do Vale, H. F., Casarin, R. C., Taiete, T., Bovi Ambrosano, G. M., Ruiz, K. G., Nociti, F. H., Jr., Sallum, E. A., & Casati, M. Z. (2016). Full-mouth ultrasonic debridement associated with povidone iodine rinsing in GAgP treatment: a randomised clinical trial. Clin Oral Investig, 20(1), 141-150. https://doi.org/10.1007/s00784-015-1471-y
52. Dominguez, A., Gomez, C., Garcia-Kass, A. I., & Garcia-Nunez, J. A. (2010). IL-1beta, TNF-alpha, total antioxidative status and microbiological findings in chronic periodontitis treated with fluorescence-controlled Er:YAG laser radiation. Lasers Surg Med, 42(1), 24-31. https://doi.org/10.1002/lsm.20873
53. Eckles, T. A., Reinhardt, R. A., Dyer, J. K., Tussing, G. J., Szydlowski, W. M., & DuBous, L. M. (1990). Intracrevicular application of tetracycline in white petrolatum for the treatment of periodontal disease. J Clin Periodontol, 17(7 Pt 1), 454-462. https://doi.org/10.1111/j.1600-051x.1990.tb02344.x
54. Ehmke, B., Schmidt, H., Beikler, T., Kopp, C., Karch, H., Klaiber, B., & Flemmig, T. F. (1999). Clonal infection with Actinobacillus actinomycetemcomitans following periodontal therapy. J Dent Res, 78(9), 1518-1524. https://doi.org/10.1177/00220345990780090601
55. Eick, S., Mathey, A., Vollroth, K., Kramesberger, M., Burgin, W., Sculean, A., Ramseier, C., & Jentsch, H. (2017). Persistence of Porphyromonas gingivalis is a negative predictor in patients with moderate to severe periodontitis after nonsurgical periodontal therapy. Clin Oral Investig, 21(2), 665-674. https://doi.org/10.1007/s00784-016-1933-x
56. Eick, S., Renatus, A., Heinicke, M., Pfister, W., Stratul, S. I., & Jentsch, H. (2013). Hyaluronic Acid as an adjunct after scaling and root planing: a prospective randomized clinical trial. J Periodontol, 84(7), 941-949. https://doi.org/10.1902/jop.2012.120269
57. Euzebio Alves, V. T., de Andrade, A. K., Toaliar, J. M., Conde, M. C., Zezell, D. M., Cai, S., Pannuti, C. M., & De Micheli, G. (2013). Clinical and microbiological evaluation of high intensity diode laser adjutant to non-surgical periodontal treatment: a 6-month clinical trial. Clin Oral Investig, 17(1), 87-95. https://doi.org/10.1007/s00784-012-0703-7
58. Everett, J. D., Rossmann, J. A., Kerns, D. G., & Al-Hashimi, I. (2017). Laser Assisted Non-surgical Periodontal Therapy: A Double Blind, Randomized Clinical Trial. Open Dent J, 11, 79-90. https://doi.org/10.2174/1874210601711010079
59. Faveri, M., Gursky, L. C., Feres, M., Shibli, J. A., Salvador, S. L., & de Figueiredo, L. C. (2006). Scaling and root planing and chlorhexidine mouthrinses in the treatment of chronic periodontitis: a randomized, placebo-controlled clinical trial. J Clin Periodontol, 33(11), 819-828. https://doi.org/10.1111/j.1600-051X.2006.00994.x
60. Feres, M., Bernal, M., Matarazzo, F., Faveri, M., Duarte, P. M., & Figueiredo, L. C. (2015). Subgingival bacterial recolonization after scaling and root planing in smokers with chronic periodontitis. Aust Dent J, 60(2), 225-232. https://doi.org/10.1111/adj.12225
61. Feres, M., Gursky, L. C., Faveri, M., Tsuzuki, C. O., & Figueiredo, L. C. (2009). Clinical and microbiological benefits of strict supragingival plaque control as part of the active phase of periodontal therapy. J Clin Periodontol, 36(10), 857-867. https://doi.org/10.1111/j.1600-051X.2009.01471.x
62. Flemmig, T. F., Petersilka, G., Volp, A., Gravemeier, M., Zilly, M., Mross, D., Prior, K., Yamamoto, J., & Beikler, T. (2011). Efficacy and safety of adjunctive local moxifloxacin delivery in the treatment of periodontitis. J Periodontol, 82(1), 96-105. https://doi.org/10.1902/jop.2010.100124
63. Fonseca, D. C., Cortelli, J. R., Cortelli, S. C., Miranda Cota, L. O., Machado Costa, L. C., Moreira Castro, M. V., Oliveira Azevedo, A. M., & Costa, F. O. (2015). Clinical and Microbiologic Evaluation of Scaling and Root Planing per Quadrant and One-Stage Full-Mouth Disinfection Associated With Azithromycin or Chlorhexidine: A Clinical Randomized Controlled Trial. J Periodontol, 86(12), 1340-1351. https://doi.org/10.1902/jop.2015.150227
64. Forgas, L. B., & Gound, S. (1987). The effects of antiformin-citric acid chemical curettage on the microbial flora of the periodontal pocket. J Periodontol, 58(3), 153-158. https://doi.org/10.1902/jop.1987.58.3.153
65. Gibson, M. T., Mangat, D., Gagliano, G., Wilson, M., Fletcher, J., Bulman, J., & Newman, H. N. (1994). Evaluation of the efficacy of a redox agent in the treatment of chronic periodontitis. J Clin Periodontol, 21(10), 690-700. https://doi.org/10.1111/j.1600-051x.1994.tb00788.x
66. Gomez, C., Dominguez, A., Garcia-Kass, A. I., & Garcia-Nunez, J. A. (2011). Adjunctive Nd:YAG laser application in chronic periodontitis: clinical, immunological, and microbiological aspects. Lasers Med Sci, 26(4), 453-463. https://doi.org/10.1007/s10103-010-0795-8
67. Gomi, K., Yashima, A., Nagano, T., Kanazashi, M., Maeda, N., & Arai, T. (2007). Effects of full-mouth scaling and root planing in conjunction with systemically administered azithromycin. J Periodontol, 78(3), 422-429. https://doi.org/10.1902/jop.2007.060247
68. Gottumukkala, S. N., Koneru, S., Mannem, S., & Mandalapu, N. (2013). Effectiveness of sub gingival irrigation of an indigenous 1% curcumin solution on clinical and microbiological parameters in chronic periodontitis patients: A pilot randomized clinical trial. Contemp Clin Dent, 4(2), 186-191. https://doi.org/10.4103/0976-237X.114874
69. Greenwell, H., 3rd, & Bissada, N. F. (1984). Variations in subgingival microflora from healthy and intervention sites using probing depth and bacteriologic identification criteria. J Periodontol, 55(7), 391-397. https://doi.org/10.1902/jop.1984.55.7.391
70. Grisi, D. C., Salvador, S. L., Figueiredo, L. C., Souza, S. L., Novaes, A. B., & Grisi, M. F. (2002). Effect of a controlled-release chlorhexidine chip on clinical and microbiological parameters of periodontal syndrome. J Clin Periodontol, 29(10), 875-881. https://doi.org/10.1034/j.1600-051x.2002.291001.x
71. Grzech-Lesniak, K., Matys, J., & Dominiak, M. (2018). Comparison of the clinical and microbiological effects of antibiotic therapy in periodontal pockets following laser treatment: An in vivo study. Adv Clin Exp Med, 27(9), 1263-1270. https://doi.org/10.17219/acem/70413
72. Guentsch, A., Jentsch, H., Pfister, W., Hoffmann, T., & Eick, S. (2008). Moxifloxacin as an adjunctive antibiotic in the treatment of severe chronic periodontitis. J Periodontol, 79(10), 1894-1903. https://doi.org/10.1902/jop.2008.070493
73. Guerrero, A., Nibali, L., Lambertenghi, R., Ready, D., Suvan, J., Griffiths, G. S., Wilson, M., & Tonetti, M. S. (2014). Impact of baseline microbiological status on clinical outcomes in generalized aggressive periodontitis patients treated with or without adjunctive amoxicillin and metronidazole: an exploratory analysis from a randomized controlled clinical trial. J Clin Periodontol, 41(11), 1080-1089. https://doi.org/10.1111/jcpe.12299
74. Haffajee, A. D., Cugini, M. A., Dibart, S., Smith, C., Kent, R. L., Jr., & Socransky, S. S. (1997a). Clinical and microbiological features of subjects with adult periodontitis who responded poorly to scaling and root planing. J Clin Periodontol, 24(10), 767-776. https://doi.org/10.1111/j.1600-051x.1997.tb00195.x
75. Haffajee, A. D., Cugini, M. A., Dibart, S., Smith, C., Kent, R. L., Jr., & Socransky, S. S. (1997b). The effect of SRP on the clinical and microbiological parameters of periodontal diseases. J Clin Periodontol, 24(5), 324-334. https://doi.org/10.1111/j.1600-051x.1997.tb00765.x
76. Haffajee, A. D., Patel, M., & Socransky, S. S. (2008). Microbiological changes associated with four different periodontal therapies for the treatment of chronic periodontitis. Oral Microbiol Immunol, 23(2), 148-157. https://doi.org/10.1111/j.1399-302X.2007.00403.x
77. Hagenfeld, D., Koch, R., Junemann, S., Prior, K., Harks, I., Eickholz, P., Hoffmann, T., Kim, T. S., Kocher, T., Meyle, J., Kaner, D., Schlagenhauf, U., Ehmke, B., & Harmsen, D. (2018). Do we treat our patients or rather periodontal microbes with adjunctive antibiotics in periodontal therapy? A 16S rDNA microbial community analysis. PLoS One, 13(4), e0195534. https://doi.org/10.1371/journal.pone.0195534
78. Han, B., Emingil, G., Özdemir, G., Tervahartiala, T., Vural, C., Atilla, G., Baylas, H., & Sorsa, T. (2012). Azithromycin as an adjunctive treatment of generalized severe chronic periodontitis: clinical, microbiologic, and biochemical parameters. J Periodontol, 83(12), 1480-1491. https://doi.org/10.1902/jop.2012.110519
79. Hayakumo, S., Arakawa, S., Mano, Y., & Izumi, Y. (2013). Clinical and microbiological effects of ozone nano-bubble water irrigation as an adjunct to mechanical subgingival debridement in periodontitis patients in a randomized controlled trial. Clin Oral Investig, 17(2), 379-388. https://doi.org/10.1007/s00784-012-0711-7
80. Heller, D., Varela, V. M., Silva-Senem, M. X., Torres, M. C., Feres-Filho, E. J., & Colombo, A. P. (2011). Impact of systemic antimicrobials combined with anti-infective mechanical debridement on the microbiota of generalized aggressive periodontitis: a 6-month RCT. J Clin Periodontol, 38(4), 355-364. https://doi.org/10.1111/j.1600-051X.2011.01707.x
81. Husejnagic, S., Lettner, S., Laky, M., Georgopoulos, A., Moritz, A., & Rausch-Fan, X. (2019). Photoactivated disinfection in periodontal treatment: A randomized controlled clinical split-mouth trial. J Periodontol, 90(11), 1260-1269. https://doi.org/10.1002/JPER.18-0576
82. Ioannou, I., Dimitriadis, N., Papadimitriou, K., Sakellari, D., Vouros, I., & Konstantinidis, A. (2009). Hand instrumentation versus ultrasonic debridement in the treatment of chronic periodontitis: a randomized clinical and microbiological trial. J Clin Periodontol, 36(2), 132-141. https://doi.org/10.1111/j.1600-051X.2008.01347.x
83. Ioannou, I., Dimitriadis, N., Papadimitriou, K., Vouros, I., Sakellari, D., & Konstantinidis, A. (2011). The effect of locally delivered doxycycline in the treatment of chronic periodontitis. A clinical and microbiological cohort study. J Oral Maxillofac Res, 1(4), e1. https://doi.org/10.5037/jomr.2010.1401
84. Isola, G., Matarese, G., Williams, R. C., Siciliano, V. I., Alibrandi, A., Cordasco, G., & Ramaglia, L. (2018). The effects of a desiccant agent in the treatment of chronic periodontitis: a randomized, controlled clinical trial. Clin Oral Investig, 22(2), 791-800. https://doi.org/10.1007/s00784-017-2154-7
85. Izidoro, C., Botelho, J., Machado, V., Reis, A. M., Proenca, L., Barroso, H., Alves, R., & Mendes, J. J. (2023). Non-Surgical Periodontal Treatment Impact on Subgingival Microbiome and Intra-Oral Halitosis. *Int J Mol Sci*, *24*(3). https://doi.org/10.3390/ijms24032518
86. Jeong, S. N., Han, S. B., Lee, S. W., & Magnusson, I. (1994). Effects of tetracycline-containing gel and a mixture of tetracycline and citric acid-containing gel on non-surgical periodontal therapy. J Periodontol, 65(9), 840-847. https://doi.org/10.1902/jop.1994.65.9.840
87. Jervøe-Storm, P. M., AlAhdab, H., Semaan, E., Fimmers, R., & Jepsen, S. (2007). Microbiological outcomes of quadrant versus full-mouth root planing as monitored by real-time PCR. J Clin Periodontol, 34(2), 156-163. https://doi.org/10.1111/j.1600-051X.2006.01035.x
88. Jones, A. A., Kornman, K. S., Newbold, D. A., & Manwell, M. A. (1994). Clinical and microbiological effects of controlled-release locally delivered minocycline in periodontitis. J Periodontol, 65(11), 1058-1066. https://doi.org/10.1902/jop.1994.65.11.1058
89. Knöfler, G. U., Purschwitz, R. E., Eick, S., Pfister, W., Roedel, M., & Jentsch, H. F. (2011). Microbiologic findings 1 year after partial- and full-mouth scaling in the treatment of moderate chronic periodontitis. Quintessence Int, 42(9), e107-117. https://www.ncbi.nlm.nih.gov/pubmed/21909493
90. Koshy, G., Kawashima, Y., Kiji, M., Nitta, H., Umeda, M., Nagasawa, T., & Ishikawa, I. (2005). Effects of single-visit full-mouth ultrasonic debridement versus quadrant-wise ultrasonic debridement. J Clin Periodontol, 32(7), 734-743. https://doi.org/10.1111/j.1600-051X.2005.00775.x
91. Leonhardt, A., Bergström, C., Krok, L., & Cardaropoli, G. (2007). Microbiological effect of the use of an ultrasonic device and iodine irrigation in patients with severe chronic periodontal disease: a randomized controlled clinical study. Acta Odontol Scand, 65(1), 52-59. https://doi.org/10.1080/00016350600973078
92. Lie, T., Bruun, G., & Böe, O. E. (1998). Effects of topical metronidazole and tetracycline in treatment of adult periodontitis. J Periodontol, 69(7), 819-827. https://doi.org/10.1902/jop.1998.69.7.819
93. Lindhe, J., Liljenberg, B., & Adielsson, B. (1983). Effect of long-term tetracycline therapy on human periodontal disease. J Clin Periodontol, 10(6), 590-601. https://doi.org/10.1111/j.1600-051x.1983.tb01297.x
94. Lindhe, J., & Nyman, S. (1985). Scaling and granulation tissue removal in periodontal therapy. J Clin Periodontol, 12(5), 374-388. https://doi.org/10.1111/j.1600-051x.1985.tb00928.x
95. Lira, E. A., Ramiro, F. S., Chiarelli, F. M., Dias, R. R., Feres, M., Figueiredo, L. C., & Faveri, M. (2013). Reduction in prevalence of Archaea after periodontal therapy in subjects with generalized aggressive periodontitis. Aust Dent J, 58(4), 442-447. https://doi.org/10.1111/adj.12123
96. Liu, G., Luan, Q., Chen, F., Chen, Z., Zhang, Q., & Yu, X. (2018). Shift in the subgingival microbiome following scaling and root planing in generalized aggressive periodontitis. J Clin Periodontol, 45(4), 440-452. https://doi.org/10.1111/jcpe.12862
97. Liu, J., Zhao, J., Li, C., Yu, N., Zhang, D., & Pan, Y. (2013). Clinical and microbiologic effect of nonsurgical periodontal therapy on patients with chronic or aggressive periodontitis. Quintessence Int, 44(8), 575-583. https://doi.org/10.3290/j.qi.a29752
98. Lombardo, G., Signoretto, C., Corrocher, G., Pardo, A., Pighi, J., Rovera, A., Caccuri, F., & Nocini, P. F. (2015). A topical desiccant agent in association with ultrasonic debridement in the initial treatment of chronic periodontitis: a clinical and microbiological study. New Microbiol, 38(3), 393-407. https://www.ncbi.nlm.nih.gov/pubmed/26147153
99. Lopes, B. M., Theodoro, L. H., Melo, R. F., Thompson, G. M., & Marcantonio, R. A. (2010). Clinical and microbiologic follow-up evaluations after non-surgical periodontal treatment with erbium:YAG laser and scaling and root planing. J Periodontol, 81(5), 682-691. https://doi.org/10.1902/jop.2010.090300
100. López, N. J., Socransky, S. S., Da Silva, I., Japlit, M. R., & Haffajee, A. D. (2006). Effects of metronidazole plus amoxicillin as the only therapy on the microbiological and clinical parameters of untreated chronic periodontitis. J Clin Periodontol, 33(9), 648-660. https://doi.org/10.1111/j.1600-051X.2006.00957.x
101. Luchesi, V. H., Pimentel, S. P., Kolbe, M. F., Ribeiro, F. V., Casarin, R. C., Nociti, F. H., Sallum, E. A., & Casati, M. Z. (2013). Photodynamic therapy in the treatment of class II furcation: a randomized controlled clinical trial. J Clin Periodontol, 40(8), 781-788. https://doi.org/10.1111/jcpe.12121
102. Lu, H., He, L., Jin, D., Zhu, Y., & Meng, H. (2022). Effect of adjunctive systemic antibiotics on microbial populations compared with scaling and root planing alone for the treatment of periodontitis: A pilot randomized clinical trial. J Periodontol, 93(4), 570-583. https://doi.org/10.1002/JPER.20-0764
103. Machion, L., Andia, D. C., Saito, D., Klein, M. I., Gonçalves, R. B., Casati, M. Z., Nociti, F. H., & Sallum, E. A. (2004). Microbiological changes with the use of locally delivered doxycycline in the periodontal treatment of smokers. J Periodontol, 75(12), 1600-1604. https://doi.org/10.1902/jop.2004.75.12.1600
104. Mahendra, J., Mahendra, L., Ananthalakshmi, R., Parthiban, P. S., Cherukuri, S., & Junaid, M. (2017). Effect of Pranayama on Ppar-γ, Nf-κB Expressions and Red Complex Microorganisms in Patients with Chronic Periodontitis - A Clinical Trial. J Clin Diagn Res, 11(6), ZC82-ZC86. https://doi.org/10.7860/JCDR/2017/27846.10108
105. Malali, E., Kadir, T., & Noyan, U. (2012). Er:YAG lasers versus ultrasonic and hand instruments in periodontal therapy: clinical parameters, intracrevicular micro-organism and leukocyte counts. Photomed Laser Surg, 30(9), 543-550. https://doi.org/10.1089/pho.2011.3202
106. Manikandan, D., Balaji, V. R., Niazi, T. M., Rohini, G., Karthikeyan, B., & Jesudoss, P. (2016). Chlorhexidine varnish implemented treatment strategy for chronic periodontitis: A clinical and microbial study. J Pharm Bioallied Sci, 8(Suppl 1), S133-S137. https://doi.org/10.4103/0975-7406.191943
107. Martande, S. S., Pradeep, A. R., Singh, S. P., Kumari, M., Naik, S. B., Suke, D. K., & Singh, P. (2016). Clinical and microbiological effects of systemic azithromycin in adjunct to nonsurgical periodontal therapy in treatment of Aggregatibacter actinomycetemcomitans associated periodontitis: a randomized placebo-controlled clinical trial. J Investig Clin Dent, 7(1), 72-80. https://doi.org/10.1111/jicd.12115
108. Martelli, F. S., Fanti, E., Rosati, C., Martelli, M., Bacci, G., Martelli, M. L., & Medico, E. (2016). Long-term efficacy of microbiology-driven periodontal laser-assisted therapy. Eur J Clin Microbiol Infect Dis, 35(3), 423-431. https://doi.org/10.1007/s10096-015-2555-y
109. Matarazzo, F., Figueiredo, L. C., Cruz, S. E., Faveri, M., & Feres, M. (2008). Clinical and microbiological benefits of systemic metronidazole and amoxicillin in the treatment of smokers with chronic periodontitis: a randomized placebo-controlled study. J Clin Periodontol, 35(10), 885-896. https://doi.org/10.1111/j.1600-051X.2008.01304.x
110. Matarese, G., Ramaglia, L., Cicciù, M., Cordasco, G., & Isola, G. (2017). The Effects of Diode Laser Therapy as an Adjunct to Scaling and Root Planing in the Treatment of Aggressive Periodontitis: A 1-Year Randomized Controlled Clinical Trial. Photomed Laser Surg, 35(12), 702-709. https://doi.org/10.1089/pho.2017.4288
111. Mdala, I., Olsen, I., Haffajee, A. D., Socransky, S. S., de Blasio, B. F., & Thoresen, M. (2013). Multilevel analysis of bacterial counts from chronic periodontitis after root planing/scaling, surgery, and systemic and local antibiotics: 2-year results. J Oral Microbiol, 5. https://doi.org/10.3402/jom.v5i0.20939
112. Mestnik, M. J., Feres, M., Figueiredo, L. C., Duarte, P. M., Lira, E. A., & Faveri, M. (2010). Short-term benefits of the adjunctive use of metronidazole plus amoxicillin in the microbial profile and in the clinical parameters of subjects with generalized aggressive periodontitis. J Clin Periodontol, 37(4), 353-365. https://doi.org/10.1111/j.1600-051X.2010.01538.x
113. Mizrak, T., Guncu, G. N., Caglayan, F., Balci, T. A., Aktar, G. S., & Ipek, F. (2006). Effect of a controlled-release chlorhexidine chip on clinical and microbiological parameters and prostaglandin E2 levels in gingival crevicular fluid. J Periodontol, 77(3), 437-443. https://doi.org/10.1902/jop.2006.050105
114. Moeintaghavi, A., Talebi-ardakani, M. R., Haerian-ardakani, A., Zandi, H., Taghipour, S., Fallahzadeh, H., Pakzad, A., & Fahami, N. (2007). Adjunctive effects of systemic amoxicillin and metronidazole with scaling and root planing: a randomized, placebo controlled clinical trial. J Contemp Dent Pract, 8(5), 51-59. https://www.ncbi.nlm.nih.gov/pubmed/17618330
115. Morales, A., Gandolfo, A., Bravo, J., Carvajal, P., Silva, N., Godoy, C., Garcia-Sesnich, J., Hoare, A., Diaz, P., & Gamonal, J. (2018). Microbiological and clinical effects of probiotics and antibiotics on nonsurgical treatment of chronic periodontitis: a randomized placebo- controlled trial with 9-month follow-up. J Appl Oral Sci, 26, e20170075. https://doi.org/10.1590/1678-7757-2017-0075
116. L, M. S., Andia, D. C., Casati, M. Z., Nociti, F. H., Jr., Sallum, E. A., Gollwitzer, J., & Walker, C. B. (2007). Microbiologic changes following administration of locally delivered doxycycline in smokers: a 15-month follow-up. J Periodontol, 78(11), 2143-2149. https://doi.org/10.1902/jop.2007.070189
117. M. Shaddox., Andia, D. C., Casati, M. Z., Nociti, F. H., Jr., Sallum, E. A., Gollwitzer, J., & Walker, C. B. (2007). Microbiologic changes following administration of locally delivered doxycycline in smokers: a 15-month follow-up. J Periodontol, 78(11), 2143-2149. https://doi.org/10.1902/jop.2007.070189
118. Nie, M., Huang, P., Peng, P., Shen, D., Zhao, L., Jiang, D., Shen, Y., Wei, L., Bible, P. W., Yang, J., Wang, J., & Wu, Y. (2024). Efficacy of photodynamic therapy as an adjunct to scaling and root planing on clinical parameters and microbial composition in subgingival plaque of periodontitis patients: A split-mouth randomized clinical trial. J Periodontol, 95(6), 535-549. https://doi.org/10.1002/JPER.23-0195
119. Novaes, A. B., Jr., Schwartz-Filho, H. O., de Oliveira, R. R., Feres, M., Sato, S., & Figueiredo, L. C. (2012). Antimicrobial photodynamic therapy in the non-surgical treatment of aggressive periodontitis: microbiological profile. Lasers Med Sci, 27(2), 389-395. https://doi.org/10.1007/s10103-011-0901-6
120. Oosterwaal, P. J., Mikx, F. H., van 't Hof, M. A., & Renggli, H. H. (1991). Comparison of the antimicrobial effect of the application of chlorhexidine gel, amine fluoride gel and stannous fluoride gel in debrided periodontal pockets. J Clin Periodontol, 18(4), 245-251. https://doi.org/10.1111/j.1600-051x.1991.tb00422.x
121. Ower, P. C., Ciantar, M., Newman, H. N., Wilson, M., & Bulman, J. S. (1995). The effects on chronic periodontitis of a subgingivally-placed redox agent in a slow release device. J Clin Periodontol, 22(6), 494-500. https://doi.org/10.1111/j.1600-051x.1995.tb00184.x
122. Paolantonio, M., D'Angelo, M., Grassi, R. F., Perinetti, G., Piccolomini, R., Pizzo, G., Annunziata, M., D'Archivio, D., D'Ercole, S., Nardi, G., & Guida, L. (2008). Clinical and microbiologic effects of subgingival controlled-release delivery of chlorhexidine chip in the treatment of periodontitis: a multicenter study. J Periodontol, 79(2), 271-282. https://doi.org/10.1902/jop.2008.070308
123. Paolantonio, M., D'Ercole, S., Pilloni, A., D'Archivio, D., Lisanti, L., Graziani, F., Femminella, B., Sammartino, G., Perillo, L., Tete, S., Perfetti, G., Spoto, G., Piccolomini, R., & Perinetti, G. (2009). Clinical, microbiologic, and biochemical effects of subgingival administration of a Xanthan-based chlorhexidine gel in the treatment of periodontitis: a randomized multicenter trial. J Periodontol, 80(9), 1479-1492. https://doi.org/10.1902/jop.2009.090050
124. Park, E. J., Kwon, E. Y., Kim, H. J., Lee, J. Y., Choi, J., & Joo, J. Y. (2018). Clinical and microbiological effects of the supplementary use of an erythritol powder air-polishing device in non-surgical periodontal therapy: a randomized clinical trial. J Periodontal Implant Sci, 48(5), 295-304. https://doi.org/10.5051/jpis.2018.48.5.295
125. Patel, P. V., Patel, A., Kumar, S., & Holmes, J. C. (2012). Effect of subgingival application of topical ozonated olive oil in the treatment of chronic periodontitis: a randomized, controlled, double blind, clinical and microbiological study. Minerva Stomatol, 61(9), 381-398. https://www.ncbi.nlm.nih.gov/pubmed/22976566
126. Penala, S., Kalakonda, B., Pathakota, K. R., Jayakumar, A., Koppolu, P., Lakshmi, B. V., Pandey, R., & Mishra, A. (2016). Efficacy of local use of probiotics as an adjunct to scaling and root planing in chronic periodontitis and halitosis: A randomized controlled trial. J Res Pharm Pract, 5(2), 86-93. https://doi.org/10.4103/2279-042X.179568
127. Peralta, F. D. S., Cortelli, S. C., Rovai, E. S., Aquino, D. R., Miranda, T. B., Costa, F. O., & Cortelli, J. R. (2020). Clinical and microbiological evaluation of non-surgical periodontal therapy in obese and non-obese individuals with periodontitis: a 9-month prospective longitudinal study. J Appl Oral Sci, 28, e20190694. https://doi.org/10.1590/1678-7757-2019-0694
128. Perayil, J., Menon, K. S., Biswas, R., Fenol, A., & Vyloppillil, R. (2016). Comparison of the efficacy of subgingival irrigation with 2% povidone-iodine and tetracycline HCl in subjects with chronic moderate periodontitis: A clinico microbiological study. Dent Res J (Isfahan), 13(2), 98-109. <https://www.ncbi.nlm.nih.gov/pubmed/27076823>
129. Pereira, A. L., Cortelli, S. C., Aquino, D. R., Franco, G. C., Cogo, K., Rodrigues, E., Costa, F. O., Holzhausen, M., & Cortelli, J. R. (2012). Reduction of salivary arginine catabolic activity through periodontal therapy. Quintessence Int, 43(9), 777-787. https://www.ncbi.nlm.nih.gov/pubmed/23041992
130. Perrella, F. A., Rovai, E. D. S., De Marco, A. C., Santamaria, M. P., Feres, M., de Figueredo, L. C., Kerbauy, W. D., & Amorim, J. B. O. (2016). Clinical and Microbiological Evaluation of Povidone-Iodine 10% as an Adjunct to Nonsurgical Periodontal Therapy in Chronic Periodontitis: A Randomized Clinical Trial. J Int Acad Periodontol, 18(4), 109-119. <https://www.ncbi.nlm.nih.gov/pubmed/31473699>
131. Polansky, R., Haas, M., Heschl, A., & Wimmer, G. (2009). Clinical effectiveness of photodynamic therapy in the treatment of periodontitis. J Clin Periodontol, 36(7), 575-580. https://doi.org/10.1111/j.1600-051x.2009.01412.x
132. Pradeep, A. R., & Kathariya, R. (2011). Clarithromycin, as an adjunct to non surgical periodontal therapy for chronic periodontitis: a double blinded, placebo controlled, randomized clinical trial. Arch Oral Biol, 56(10), 1112-1119. https://doi.org/10.1016/j.archoralbio.2011.03.021
133. Pradeep, A. R., Priyanka, N., Kalra, N., & Naik, S. B. (2013). A randomized controlled clinical trial on the clinical and microbiological efficacy of systemic satranidazole in the treatment of chronic periodontitis. J Int Acad Periodontol, 15(2), 43-50. https://www.ncbi.nlm.nih.gov/pubmed/23705535
134. Pradeep, A. R., Sagar, S. V., & Daisy, H. (2008). Clinical and microbiologic effects of subgingivally delivered 0.5% azithromycin in the treatment of chronic periodontitis. J Periodontol, 79(11), 2125-2135. https://doi.org/10.1902/jop.2008.070589
135. Pradeep, A. R., Singh, S. P., Martande, S. S., Naik, S. B., Kalra, N., & Priyanka, N. (2014). Clinical and microbiological effects of levofloxacin in the treatment of Aggregatibacter actinomycetemcomitans-associated periodontitis: a randomized placebo-controlled clinical trial. J Int Acad Periodontol, 16(3), 67-77. https://www.ncbi.nlm.nih.gov/pubmed/25654959
136. Pradeep, A. R., Singh, S. P., Martande, S. S., Naik, S. B., N, P., Kalra, N., & Suke, D. K. (2015). Clinical and microbiological effects of levofloxacin in the treatment of chronic periodontitis: a randomized, placebo-controlled clinical trial. J Investig Clin Dent, 6(3), 170-178. https://doi.org/10.1111/jicd.12091
137. Preber, H., Linder, L., & Bergstrom, J. (1995). Periodontal healing and periopathogenic microflora in smokers and non-smokers. J Clin Periodontol, 22(12), 946-952. https://doi.org/10.1111/j.1600-051x.1995.tb01800.x
138. Priyanka, N., Kalra, N., Saquib, S., Kudyar, N., Malgaonkar, N., Jain, H., & Pradeep, A. R. (2015). Clinical and microbiological efficacy of 3% satranidazole gel as a local drug delivery system in the treatment of chronic periodontitis: A randomized, controlled clinical trial. Contemp Clin Dent, 6(3), 364-370. https://doi.org/10.4103/0976-237X.161891
139. Pulikkotil, S. J., Toh, C. G., Mohandas, K., & Leong, K. (2016). Effect of photodynamic therapy adjunct to scaling and root planing in periodontitis patients: A randomized clinical trial. Aust Dent J, 61(4), 440-445. https://doi.org/10.1111/adj.12409
140. Querido, S. M., Cortelli, S. C., Araujo, M. W., & Cortelli, J. R. (2004). Clinical and microbial evaluation of dental scaling associated with subgingival minocycline in chronic periodontitis subjects. Braz Oral Res, 18(2), 110-115. https://doi.org/10.1590/s1806-83242004000200004
141. Quirynen, M., Bollen, C. M., Vandekerckhove, B. N., Dekeyser, C., Papaioannou, W., & Eyssen, H. (1995). Full- vs. partial-mouth disinfection in the treatment of periodontal infections: short-term clinical and microbiological observations. J Dent Res, 74(8), 1459-1467. https://doi.org/10.1177/00220345950740080501
142. Quirynen, M., Mongardini, C., de Soete, M., Pauwels, M., Coucke, W., van Eldere, J., & van Steenberghe, D. (2000). The role of chlorhexidine in the one-stage full-mouth disinfection treatment of patients with advanced adult periodontitis. Long-term clinical and microbiological observations. J Clin Periodontol, 27(8), 578-589. https://doi.org/10.1034/j.1600-051x.2000.027008578.x
143. Quirynen, M., Mongardini, C., Pauwels, M., Bollen, C. M., Van Eldere, J., & van Steenberghe, D. (1999). One stage full- versus partial-mouth disinfection in the treatment of chronic adult or generalized early-onset periodontitis. II. Long-term impact on microbial load. J Periodontol, 70(6), 646-656. https://doi.org/10.1902/jop.1999.70.6.646
144. Raj, K. R., Musalaiah, S., Nagasri, M., Kumar, P. A., Reddy, P. I., & Greeshma, M. (2016). Evaluation of efficacy of photodynamic therapy as an adjunct to nonsurgical periodontal therapy in treatment of chronic periodontitis patients: A clinico-microbiological study. Indian J Dent Res, 27(5), 483-487. https://doi.org/10.4103/0970-9290.195622
145. Ramiro, F. S., de Lira, E., Soares, G., Retamal-Valdes, B., Feres, M., Figueiredo, L. C., & Faveri, M. (2018). Effects of different periodontal treatments in changing the prevalence and levels of Archaea present in the subgingival biofilm of subjects with periodontitis: A secondary analysis from a randomized controlled clinical trial. Int J Dent Hyg, 16(4), 569-575. https://doi.org/10.1111/idh.12347
146. Rassameemasmaung, S., Sirikulsathean, A., Amornchat, C., Maungmingsook, P., Rojanapanthu, P., & Gritsanaphan, W. (2008). Topical application of Garcinia mangostana L. pericarp gel as an adjunct to periodontal treatment. Complement Ther Med, 16(5), 262-267. https://doi.org/10.1016/j.ctim.2007.12.004
147. Ready, D., D'Aiuto, F., Spratt, D. A., Suvan, J., Tonetti, M. S., & Wilson, M. (2008). Disease severity associated with presence in subgingival plaque of Porphyromonas gingivalis, Aggregatibacter actinomycetemcomitans, and Tannerella forsythia, singly or in combination, as detected by nested multiplex PCR. J Clin Microbiol, 46(10), 3380-3383. https://doi.org/10.1128/JCM.01007-08
148. Renvert, S., Dahlen, G., & Snyder, B. (1997). Clinical and microbiological effects of subgingival antimicrobial irrigation with citric acid as evaluated by an enzyme immunoassay and culture analysis. J Periodontol, 68(4), 346-352. https://doi.org/10.1902/jop.1997.68.4.346
149. Renvert, S., Dahlen, G., & Wikstrom, M. (1998). The clinical and microbiological effects of non-surgical periodontal therapy in smokers and non-smokers. J Clin Periodontol, 25(2), 153-157. https://doi.org/10.1111/j.1600-051x.1998.tb02421.x
150. Renvert, S., Wikstrom, M., Helmersson, M., Dahlen, G., & Claffey, N. (1992). Comparative study of subgingival microbiological sampling techniques. J Periodontol, 63(10), 797-801. https://doi.org/10.1902/jop.1992.63.10.797
151. Roman-Torres, C. V. G., Bryington, M. S., Kussaba, S. T., Pimentel, A. C., Jimbo, R., Cortelli, J. R., & Romito, G. A. (2018). Comparison Of Full-Mouth Scaling and Quadrant-Wise Scaling in the Treatment of Adult Chronic Periodontitis. Braz Dent J, 29(3), 296-300. https://doi.org/10.1590/0103-6440201801715
152. Rooney, J., Wade, W. G., Sprague, S. V., Newcombe, R. G., & Addy, M. (2002). Adjunctive effects to non-surgical periodontal therapy of systemic metronidazole and amoxycillin alone and combined. A placebo controlled study. J Clin Periodontol, 29(4), 342-350. https://doi.org/10.1034/j.1600-051x.2002.290410.x
153. Rosalem, W., Rescala, B., Teles, R. P., Fischer, R. G., Gustafsson, A., & Figueredo, C. M. (2011). Effect of non-surgical treatment on chronic and aggressive periodontitis: clinical, immunologic, and microbiologic findings. J Periodontol, 82(7), 979-989. https://doi.org/10.1902/jop.2011.100579
154. Rosling, B. G., Slots, J., Webber, R. L., Christersson, L. A., & Genco, R. J. (1983). Microbiological and clinical effects of topical subgingival antimicrobial treatment on human periodontal disease. J Clin Periodontol, 10(5), 487-514. https://doi.org/10.1111/j.1600-051x.1983.tb02180.x
155. Saglam, M., Arslan, U., Buket Bozkurt, S., & Hakki, S. S. (2013). Boric acid irrigation as an adjunct to mechanical periodontal therapy in patients with chronic periodontitis: a randomized clinical trial. J Periodontol, 84(9), 1297-1308. https://doi.org/10.1902/jop.2012.120467
156. Saglam, M., Koseoglu, S., Tasdemir, I., Erbak Yilmaz, H., Savran, L., & Sutcu, R. (2017). Combined application of Er:YAG and Nd:YAG lasers in treatment of chronic periodontitis. A split-mouth, single-blind, randomized controlled trial. J Periodontal Res, 52(5), 853-862. https://doi.org/10.1111/jre.12454
157. Sakellari, D., Ioannidis, I., Antoniadou, M., Slini, T., & Konstantinidis, A. (2010). Clinical and microbiological effects of adjunctive, locally delivered chlorhexidine on patients with chronic periodontitis. J Int Acad Periodontol, 12(1), 20-26. https://www.ncbi.nlm.nih.gov/pubmed/20593635
158. Sampaio, E., Rocha, M., Figueiredo, L. C., Faveri, M., Duarte, P. M., Gomes Lira, E. A., & Feres, M. (2011). Clinical and microbiological effects of azithromycin in the treatment of generalized chronic periodontitis: a randomized placebo-controlled clinical trial. J Clin Periodontol, 38(9), 838-846. https://doi.org/10.1111/j.1600-051X.2011.01766.x
159. Sanz-Sanchez, I., Ortiz-Vigon, A., Herrera, D., & Sanz, M. (2016). Microbiological effects and recolonization patterns after adjunctive subgingival debridement with Er:YAG laser. Clin Oral Investig, 20(6), 1253-1261. https://doi.org/10.1007/s00784-015-1617-y
160. Sbordone, L., Ramaglia, L., Gulletta, E., & Iacono, V. (1990). Recolonization of the subgingival microflora after scaling and root planing in human periodontitis. J Periodontol, 61(9), 579-584. https://doi.org/10.1902/jop.1990.61.9.579
161. Schwarz, F., Sculean, A., Berakdar, M., Georg, T., Reich, E., & Becker, J. (2003a). Clinical evaluation of an Er:YAG laser combined with scaling and root planing for non-surgical periodontal treatment. A controlled, prospective clinical study. J Clin Periodontol, 30(1), 26-34. https://doi.org/10.1034/j.1600-051x.2003.300105.x
162. Schwarz, F., Sculean, A., Berakdar, M., Georg, T., Reich, E., & Becker, J. (2003b). Periodontal treatment with an Er:YAG laser or scaling and root planing. A 2-year follow-up split-mouth study. J Periodontol, 74(5), 590-596. https://doi.org/10.1902/jop.2003.74.5.590
163. Schwarz, F., Sculean, A., Georg, T., & Reich, E. (2001). Periodontal treatment with an Er: YAG laser compared to scaling and root planing. A controlled clinical study. J Periodontol, 72(3), 361-367. https://doi.org/10.1902/jop.2001.72.3.361
164. Sefton, A. M., Maskell, J. P., Beighton, D., Whiley, A., Shain, H., Foyle, D., Smith, S. R., Smales, F. C., & Williams, J. D. (1996). Azithromycin in the treatment of periodontal disease. Effect on microbial flora. J Clin Periodontol, 23(11), 998-1003. https://doi.org/10.1111/j.1600-051x.1996.tb00527.x
165. Shiloah, J., Patters, M. R., Dean, J. W., 3rd, Bland, P., & Toledo, G. (1997). The survival rate of Actinobacillus actinomycetemcomitans, Porphyromonas gingivalis, and Bacteroides forsythus following 4 randomized treatment modalities. J Periodontol, 68(8), 720-728. https://doi.org/10.1902/jop.1997.68.8.720
166. Shiloah, J., Patters, M. R., Dean, J. W., 3rd, Bland, P., & Toledo, G. (1998). The prevalence of Actinobacillus actinomycetemcomitans, Porphyromonas gingivalis, and Bacteroides forsythus in humans 1 year after 4 randomized treatment modalities. J Periodontol, 69(12), 1364-1372. https://doi.org/10.1902/jop.1998.69.12.1364
167. Silva, M. P., Feres, M., Sirotto, T. A., Soares, G. M., Mendes, J. A., Faveri, M., & Figueiredo, L. C. (2011). Clinical and microbiological benefits of metronidazole alone or with amoxicillin as adjuncts in the treatment of chronic periodontitis: a randomized placebo-controlled clinical trial. J Clin Periodontol, 38(9), 828-837. https://doi.org/10.1111/j.1600-051X.2011.01763.x
168. Silva-Boghossian, C. M., Orrico, S. R., Goncalves, D., Correa, F. O., & Colombo, A. P. (2014). Microbiological changes after periodontal therapy in diabetic patients with inadequate metabolic control. Braz Oral Res, 28. https://doi.org/10.1590/1807-3107bor-2014.vol28.0007
169. Silva-Senem, M. X., Heller, D., Varela, V. M., Torres, M. C., Feres-Filho, E. J., & Colombo, A. P. (2013). Clinical and microbiological effects of systemic antimicrobials combined to an anti-infective mechanical debridement for the management of aggressive periodontitis: a 12-month randomized controlled trial. J Clin Periodontol, 40(3), 242-251. https://doi.org/10.1111/jcpe.12052
170. Sindhura, H., Harsha, R. H., & Shilpa, R. H. (2017). Efficacy of subgingival irrigation with 10% povidone-iodine as an adjunct to scaling and root planing: A clinical and microbiological study. Indian J Dent Res, 28(5), 514-518. https://doi.org/10.4103/ijdr.IJDR_497_15
171. Soares, G. M., Mendes, J. A., Silva, M. P., Faveri, M., Teles, R., Socransky, S. S., Wang, X., Figueiredo, L. C., & Feres, M. (2014). Metronidazole alone or with amoxicillin as adjuncts to non-surgical treatment of chronic periodontitis: a secondary analysis of microbiological results from a randomized clinical trial. J Clin Periodontol, 41(4), 366-376. https://doi.org/10.1111/jcpe.12217
172. Soeroso, Y., Akase, T., Sunarto, H., Kemal, Y., Salim, R., Octavia, M., Viandita, A., Setiawan, J., & Bachtiar, B. M. (2017). The risk reduction of recurrent periodontal pathogens of local application minocycline HCl 2% gel, used as an adjunct to scaling and root planing for chronic periodontitis treatment. Ther Clin Risk Manag, 13, 307-314. https://doi.org/10.2147/TCRM.S130257
173. Spooner, R., Weigel, K. M., Harrison, P. L., Lee, K., Cangelosi, G. A., & Yilmaz, O. (2016). In Situ Anabolic Activity of Periodontal Pathogens Porphyromonas gingivalis and Filifactor alocis in Chronic Periodontitis. Sci Rep, 6, 33638. https://doi.org/10.1038/srep33638
174. Sreedhar, A., Sarkar, I., Rajan, P., Pai, J., Malagi, S., Kamath, V., & Barmappa, R. (2015). Comparative evaluation of the efficacy of curcumin gel with and without photo activation as an adjunct to scaling and root planing in the treatment of chronic periodontitis: A split mouth clinical and microbiological study. J Nat Sci Biol Med, 6(Suppl 1), S102-109. https://doi.org/10.4103/0976-9668.166100
175. Suchetha, A., & Bharwani, A. G. (2013). Efficacy of a commercially available multi-herbal formulation in periodontal therapy. J Indian Soc Periodontol, 17(2), 193-197. https://doi.org/10.4103/0972-124X.113068
176. Suryaprasanna, J., Radhika, P. L., Karunakar, P., Rekharani, K., Faizuddin, U., Manojkumar, M. G., & Jammula, S. (2018). Evaluating the effectiveness of clarithromycin as an adjunct to scaling and root planing: A randomized clinical trial. J Indian Soc Periodontol, 22(6), 529-534. https://doi.org/10.4103/jisp.jisp_254_18
177. Swierkot, K., Nonnenmacher, C. I., Mutters, R., Flores-de-Jacoby, L., & Mengel, R. (2009). One-stage full-mouth disinfection versus quadrant and full-mouth root planing. J Clin Periodontol, 36(3), 240-249. https://doi.org/10.1111/j.1600-051X.2008.01368.x
178. Tabenski, L., Moder, D., Cieplik, F., Schenke, F., Hiller, K. A., Buchalla, W., Schmalz, G., & Christgau, M. (2017). Antimicrobial photodynamic therapy vs. local minocycline in addition to non-surgical therapy of deep periodontal pockets: a controlled randomized clinical trial. Clin Oral Investig, 21(7), 2253-2264. https://doi.org/10.1007/s00784-016-2018-6
179. Talebi, M., Taliee, R., Mojahedi, M., Meymandi, M., & Torshabi, M. (2016). Microbiological Efficacy of Photodynamic Therapy as an Adjunct to Non-surgical Periodontal Treatment: A Clinical Trial. J Lasers Med Sci, 7(2), 126-130. https://doi.org/10.15171/jlms.2016.21
180. Tanaka, M. H., Rodrigues, T. O., Finoti, L. S., Teixeira, S. R., Mayer, M. P., Scarel-Caminaga, R. M., & Giro, E. M. (2015). The effect of conventional mechanical periodontal treatment on red complex microorganisms and clinical parameters in Down syndrome periodontitis patients: a pilot study. Eur J Clin Microbiol Infect Dis, 34(3), 601-608. https://doi.org/10.1007/s10096-014-2268-7
181. Tekce, M., Ince, G., Gursoy, H., Dirikan Ipci, S., Cakar, G., Kadir, T., & Yilmaz, S. (2015). Clinical and microbiological effects of probiotic lozenges in the treatment of chronic periodontitis: a 1-year follow-up study. J Clin Periodontol, 42(4), 363-372. https://doi.org/10.1111/jcpe.12387
182. Teughels, W., Durukan, A., Ozcelik, O., Pauwels, M., Quirynen, M., & Haytac, M. C. (2013). Clinical and microbiological effects of Lactobacillus reuteri probiotics in the treatment of chronic periodontitis: a randomized placebo-controlled study. J Clin Periodontol, 40(11), 1025-1035. https://doi.org/10.1111/jcpe.12155
183. Theodoro, L. H., Assem, N. Z., Longo, M., Alves, M. L. F., Duque, C., Stipp, R. N., Vizoto, N. L., & Garcia, V. G. (2018). Treatment of periodontitis in smokers with multiple sessions of antimicrobial photodynamic therapy or systemic antibiotics: A randomized clinical trial. Photodiagnosis Photodyn Ther, 22, 217-222. https://doi.org/10.1016/j.pdpdt.2018.04.003
184. Theodoro, L. H., Silva, S. P., Pires, J. R., Soares, G. H., Pontes, A. E., Zuza, E. P., Spolidorio, D. M., de Toledo, B. E., & Garcia, V. G. (2012). Clinical and microbiological effects of photodynamic therapy associated with nonsurgical periodontal treatment. A 6-month follow-up. Lasers Med Sci, 27(4), 687-693. https://doi.org/10.1007/s10103-011-0942-x
185. Timmerman, M. F., van der Weijden, G. A., van Steenbergen, T. J., Mantel, M. S., de Graaff, J., & van der Velden, U. (1996). Evaluation of the long-term efficacy and safety of locally-applied minocycline in adult periodontitis patients. J Clin Periodontol, 23(8), 707-716. https://doi.org/10.1111/j.1600-051x.1996.tb00599.x
186. Uraz, A., Karaduman, B., Isler, S. C., Gonen, S., & Cetiner, D. (2019). Ozone application as adjunctive therapy in chronic periodontitis: Clinical, microbiological and biochemical aspects. J Dent Sci, 14(1), 27-37. https://doi.org/10.1016/j.jds.2018.06.005
187. Winkel, E. G., van Winkelhoff, A. J., Barendregt, D. S., van der Weijden, G. A., Timmerman, M. F., & van der Velden, U. (1999). Clinical and microbiological effects of initial periodontal therapy in conjunction with amoxicillin and clavulanic acid in patients with adult periodontitis. A randomised double-blind, placebo-controlled study. J Clin Periodontol, 26(7), 461-468. https://doi.org/10.1034/j.1600-051x.1999.260708.x
188. Winkel, E. G., Van Winkelhoff, A. J., Timmerman, M. F., Van der Velden, U., & Van der Weijden, G. A. (2001). Amoxicillin plus metronidazole in the treatment of adult periodontitis patients. A double-blind placebo-controlled study. J Clin Periodontol, 28(4), 296-305. https://doi.org/10.1034/j.1600-051x.2001.028004296.x
189. Xajigeorgiou, C., Sakellari, D., Slini, T., Baka, A., & Konstantinidis, A. (2006). Clinical and microbiological effects of different antimicrobials on generalized aggressive periodontitis. J Clin Periodontol, 33(4), 254-264. https://doi.org/10.1111/j.1600-051X.2006.00905.x
190. Yashima, A., Gomi, K., Maeda, N., & Arai, T. (2009). One-stage full-mouth versus partial-mouth scaling and root planing during the effective half-life of systemically administered azithromycin. J Periodontol, 80(9), 1406-1413. https://doi.org/10.1902/jop.2009.090067
191. Yek, E. C., Cintan, S., Topcuoglu, N., Kulekci, G., Issever, H., & Kantarci, A. (2010). Efficacy of amoxicillin and metronidazole combination for the management of generalized aggressive periodontitis. J Periodontol, 81(7), 964-974. https://doi.org/10.1902/jop.2010.090522
192. Yeom, H. R., Park, Y. J., Lee, S. J., Rhyu, I. C., Chung, C. P., & Nisengard, R. J. (1997). Clinical and microbiological effects of minocycline-loaded microcapsules in adult periodontitis. J Periodontol, 68(11), 1102-1109. https://doi.org/10.1902/jop.1997.68.11.1102
193. Yilmaz, S., Algan, S., Gursoy, H., Noyan, U., Kuru, B. E., & Kadir, T. (2013). Evaluation of the clinical and antimicrobial effects of the Er:YAG laser or topical gaseous ozone as adjuncts to initial periodontal therapy. Photomed Laser Surg, 31(6), 293-298. https://doi.org/10.1089/pho.2012.3379
194. Yilmaz, S., Kut, B., Gursoy, H., Eren-Kuru, B., Noyan, U., & Kadir, T. (2012). Er:YAG laser versus systemic metronidazole as an adjunct to nonsurgical periodontal therapy: a clinical and microbiological study. Photomed Laser Surg, 30(6), 325-330. https://doi.org/10.1089/pho.2010.2762
195. Zengin Celik, T., Saglam, E., Ercan, C., Akbas, F., Nazaroglu, K., & Tunali, M. (2019). Clinical and Microbiological Effects of the Use of Erbium: Yttrium-Aluminum-Garnet Laser on Chronic Periodontitis in Addition to Nonsurgical Periodontal Treatment: A Randomized Clinical Trial-6 Months Follow-Up. Photobiomodul Photomed Laser Surg, 37(3), 182-190. https://doi.org/10.1089/photob.2018.4510
196. Zijnge, V., Meijer, H. F., Lie, M. A., Tromp, J. A., Degener, J. E., Harmsen, H. J., & Abbas, F. (2010). The recolonization hypothesis in a full-mouth or multiple-session treatment protocol: a blinded, randomized clinical trial. J Clin Periodontol, 37(6), 518-525. https://doi.org/10.1111/j.1600-051X.2010.01562.x

**Appendix S6.** Primary outcome reporting of checkerboard DNA-DNA hybridization technology studies (n = 41).

| **Authors, year** | **Sampling site** | **Method** | **Pooled/ SS** | **Species** | **Variability** | **X** |
| --- | --- | --- | --- | --- | --- | --- |
| Apatzidou et al. 2014 | Deepest site per quadrant, PPD ≤ 5 mm | Curette | Pooled | *Pg, Td* and *Tf* | SD |  |
| Borekci et al. 2019 | Five distinct sites, initial PPD ≤ 5 mm | PP | Pooled | *Aa, Pg, Pi, Td* and *Tf* | SD |  |
| Bozoglan et al. 2017 | Deepest site PPD ≤ 5 mm | PP | Pooled | *Aa, Pg, Td, Tf, Pi, Pm, Fn, Cr, En, Ec* and *Capn. Sp*. | P | (c) |
| Brochut et al. 2005 | 4 deepest site PPD ≤ 5 mm | PP | SS | *Aa, Pg, Td* and *Tf* | SD |  |
| Carvalho et al. 2005 | 4 sites PPD 6 - 10 mm | Curette | SS | Panel | P |  |
| Christgau et al. 2006 | Deepest site per quadrant | PP | Pooled | *Aa, Pg, Td* and *Tf* | SD |  |
| Christgau et al. 2007 | Deepest site per quadrant | PP | Pooled | *Aa, Pg, Td* and *Tf* | SD |  |
| Colombo et al. 2005 | 10 deepest sites PPD ≤ 5 mm | Curette | SS | Panel | P |  |
| Cugini et al. 2000 | Mesiobuccal aspect of each tooth | Curette | SS | Panel | P | (b) |
| De Melo Soares et al. 2019 | Contralateral sites of proximal tooth surface as test and control, PPD ≥ 5 mm | Curette | SS | Complexes | None | (a) |
| Feres et al. 2009 | 6 proximal sites per subject PPD 5 - 7mm | Curette | SS | Panel | P |  |
| Feres et al. 2015 | 6 proximal sites per subject PPD 5 - 7 mm | Curette | SS | Panel | P |  |
| Haffajee et al. 1997_2 | Mesiobuccal aspect of each tooth | Curette | SS | Panel | SE | (b) |
| Haffajee et al. 1997 | Mesiobuccal aspect of each tooth | Curette | SS | Panel | SE | (b) |
| Haffajee et al. 2008 | Mesiobuccal aspect of each tooth | Curette | SS | Panel | P |  |
| Heller et al. 2011 | 5 sites PPD 4–6 mm and 5 sites PPD 7 mm | Curette | SS | Panel | P |  |
| Ioannou et al. 2009 | 2 sites PPD 4-6 mm and 2 sites PPD < 6 mm | Curette | SS | *Aa, Pg, Td* and *Tf* | SE |  |
| Ioannou et al. 2011 | 2 sites PPD 4-6 mm and 2 sites PPD < 6 mm | Curette | SS | *Pg, Td* and *Tf* | SE |  |
| Isola et al. 2018 | 6 interproximal sites | PP | SS | Panel | P |  |
| Jones et al. 1994 | 2 sites | N/I | SS | *Pg, Pi, Cr,* *Ec* and *Fn* | None | (a) |
| Leonhardt et al. 2007 | 1 deepest site | PP | SS | *Aa, Td, Pg, Tf, Pi, Fn, Pn* and *Pm* | P | (a) |
| Lopez et al. 2006 | Mesiobuccal aspect of each tooth | Curette | SS | Panel | SE |  |
| Matarazzo et al. 2008 | 3 interproximal sites PPD 4-6 mm and 3 interproximal sites PPD < 6 mm | Curette | SS | Panel | P | (c) |
| Matarese et al. 2017 | 8 interproximal sites | PP | SS | Panel | P |  |
| Mdala et al. 2013 | Mesiobuccal aspect of each tooth | Curette | SS | *Aa* and red complex | None | (b) |
| Mestnik et al. 2010 | 3 interproximal sites PPD 4-6 mm and 3 interproximal sites PPD < 6 mm | Curette | SS | Panel | P |  |
| Moreira et al. 2015 | 8 interproximal sites | Curette | SS | Panel | P |  |
| Novaes et al. 2012 | 10 contra-lateral single rooted teeth | Curette | SS | Panel | SD/ P |  |
| Perrella et al. 2016 | 3 interproximal sites PPD 4-6 mm and 3 interproximal sites PPD < 6 mm | Curette | SS | Panel | P |  |
| Rosalem et al. 2011 | Mesiobuccal aspect of each tooth in 2 quadrants | Curette | SS | Panel | P |  |
| Sakellari et al. 2010 | 4 sites with Periochip^®^ (Group 1) and 4 sites PPD ≥ 5 mm and ≤ 7mm | Curette | SS | *Pg, Tf, Td, Pn, Pi, Fn, Cr* and *Vp* | SE |  |
| Sampaio et al. 2011 | 3 interproximal sites PPD 4-6 mm and 3 interproximal sites PPD < 6 mm | Curette | SS | Panel | P |  |
| Shiloa et al. 1997 | 1 site per quadrant, split-mouth design | PP | SS | *Aa, Pg* and *Tf* | SE |  |
| Shiloa et al. 1998 | 1 site per quadrant, split-mouth design | PP | SS | *Aa, Pg* and *Tf* | SE | (b) |
| Silva 2011 | 3 interproximal sites PPD 4-6 mm and 3 interproximal sites PPD < 6 mm | Curette | SS | Panel | P |  |
| Silva-Boghossian et al. 2014 | 4 deep sites (PPD ≥ 5 mm, CAL ≥ 4 mm plus FMBS) non-adjacent teeth | Curette | SS | Panel | SE |  |
| Silva-Senem et al. 2013 | 5 sites PPD 4–6 mm and  5 sites PPD ≥ 7 mm | Curette | SS | Panel | P | (b) |
| Soares et al. 2014 | 3 interproximal sites PPD 4-6 mm and  3 interproximal sites PPD < 6 mm | Curette | SS | Panel | P |  |
| Tabenski et al. 2017 | 4 deepest sites | N/I | N/I | *Aa, Tf, Pg* and *Td* | P | (b) |
| Timmerman et al. 1996 | 4 deepest sites | PP | Pooled | *Aa, Pg, Pi, Fn, Cr* and *Td* | SD |  |
| Xajigeorgiou et al. 2006 | mb sites, 10 pre-selected teeth PPD 5 > mm | Curette | SS | *Aa, Pg, Td* and *Tf* | None | (a) |
| PP: paper point, SS: site specific, N/I: no information, *Aa*: *Aggregatibacter actinomycetemcomitans*, *An*: *Actinomyces naeslundii*, *Cg:* *Capnocytophaga gingivalis*, *Cr*: *Camphylobacter rectus*, *Ec*: *Eikenella corrodens*, *Fn*: *Fusobacterium nucleatum*, *Pi*: *Prevotella intermedia*, Pg: *Porphyromonas gingivalis*, Pm: *Peptostreptococcus micra*: *Pn*: *Prevotella nigrescens*, *Td*: *Treponema denticola*, *Tf:* *Tannerella forsythia*, *So*: *Streptococcus oralis*, *Capn*. *sp*.: *Caphnophaga species*, SD: standard deviation, P: p-value, SE: standard error, CI: confidence interval, X: reason for exclusion from meta-analysis, (a): no measure of variation, (b): no mean values, (c): p-values, but no sig. differences | | | | | | |

**Appendix S7.** Microbiological findings in checkerboard DNA-DNA hybridization technology studies without adjunctive therapy at 3 months after NSPT, (A) according to periodontal diagnosis, blue: chronic periodontitis, yellow: aggressive periodontitis and (B) smoking status, blue: non-smokers, yellow: smokers. Turquoise indicates a decrease in the mean counts, grey no changes in the mean counts, and orange an increase in mean counts of subgingival bacteria after NSPT. Black indicates that microbiological data is not reported. * Indicates results that were reported as statistically significant (p-value ≤ 0.05). PC: weekly professional plaque control. Bacterial abbreviations: *Ag: A. gerencseriae, Ai: A. israelli, An: A. naeslundii, Ao: A. odontolyticus, Vp: V. parvula, Sg: S. gordonii, Si: S. intermedius, Sm: S. mitis, So: S. oralis, Ss: S. sanguis, Aa: A. actinomycetemcomitans a, Cg: C. gingivalis, Co: C. ochracea, Csp: C. sputigena, Ec: E. corrodens, Cg: C. gracilis, Cr: C. rectus, Csh: C. showae, En: E. nodatum, Fnn: F. n. nucleatum, Fnp: F. n. polymorphum, Fnv: F. n. vincentii, Fp: F. periodonticum, Pm: P. micros, Pi: P. intermedia, Pn: P. nigrescens, Sc: S. constellatus, Tf: T. forsythia, Pg: P. gingivalis, Td: T. denticola, Es: E. saburreum, Gm: G. morbillorum, Lb: L. buccalis, Nm: N. mucosa, Pa: P. acnes, Pme: P. melaninogenica, Sa: S. anginosus, Sn: S. noxia, Ts: T. socranskii.*

**Appendix S8.** Microbiological findings in Checkerboard DNA-DNA hybridization technology studies according to adjunctive therapy, blue: no adjunctive therapy, yellow: with adjunctive therapy. Turquoise indicates a decrease in the mean counts, grey no changes in the mean counts, and orange an increase in mean counts of subgingival bacteria after NSPT. Black indicates that microbiological data is not reported. * Indicates results that were reported as statistically significant (p-value ≤ 0.05).PC: weekly professional plaque control, Bacterial abbreviations: *Ag: A. gerencseriae, Ai: A. israelli, An: A. naeslundii, Ao: A. odontolyticus, Vp: V. parvula, Sg: S. gordonii, Si: S. intermedius, Sm: S. mitis, So: S. oralis, Ss: S. sanguis, Aa: A. actinomycetemcomitans a, Cg: C. gingivalis, Co: C. ochracea, Csp: C. sputigena, Ec: E. corrodens, Cg: C. gracilis, Cr: C. rectus, Csh: C. showae, En: E. nodatum, Fnn: F. n. nucleatum, Fnp: F. n. polymorphum, Fnv: F. n. vincentii, Fp: F. periodonticum, Pm: P. micros, Pi: P. intermedia, Pn: P. nigrescens, Sc: S. constellatus, Tf: T. forsythia, Pg: P. gingivalis, Td: T. denticola, Es: E. saburreum, Gm: G. morbillorum, Lb: L. buccalis, Nm: N. mucosa, Pa: P. acnes, Pme: P. melaninogenica, Sa: S. anginosus, Sn: S. noxia, Ts: T. socranskii.*

**Appendix S9**. Microbiological findings in Checkerboard DNA-DNA hybridization technology studies according to supportive periodontal care (SPC) provision in studies without adjunctive therapies, blue: without SPC, yellow with SPC. Turquoise indicates a decrease in the mean counts, grey no changes in the mean counts, and orange an increase in mean counts of subgingival bacteria after NSPT. Black indicates that microbiological data is not reported. * Indicates results that were reported as statistically significant (p-value ≤ 0.05). PC: weekly professional plaque control, SPC: supportive periodontal care, Bacterial abbreviations: *Ag: A. gerencseriae, Ai: A. israelli, An: A. naeslundii, Ao: A. odontolyticus, Vp: V. parvula, Sg: S. gordonii, Si: S. intermedius, Sm: S. mitis, So: S. oralis, Ss: S. sanguis, Aa: A. actinomycetemcomitans a, Cg: C. gingivalis, Co: C. ochracea, Csp: C. sputigena, Ec: E. corrodens, Cg: C. gracilis, Cr: C. rectus, Csh: C. showae, En: E. nodatum, Fnn: F. n. nucleatum, Fnp: F. n. polymorphum, Fnv: F. n. vincentii, Fp: F. periodonticum, Pm: P. micros, Pi: P. intermedia, Pn: P. nigrescens, Sc: S. constellatus, Tf: T. forsythia, Pg: P. gingivalis, Td: T. denticola, Es: E. saburreum, Gm: G. morbillorum, Lb: L. buccalis, Nm: N. mucosa, Pa: P. acnes, Pme: P. melaninogenica, Sa: S. anginosus, Sn: S. noxia, Ts: T. socranskii.*

**Appendix S10.** Bacterial species on checkerboard panel, organised according to Socransky complexes.

| Blue *(actinomyces)* | *Actinomyces gerenecseriae*  *Actinomyces israelii*  *Actinomyces naeslundii*  *Actinomyces oris* |
| --- | --- |
| Purple | *Actinomyces odontolyticus*  *Veinon parvula* |
| Yellow | *Streptococcus gordonii*  *Streptococcus intermedius*  *Streptococcus mitis*  *Streptococcus oralis*  *Streptococcus sanguis* |
| Green | *Aggregatibacter actinomycetemcomitans*  *Capnocytophaga gingivalis*  *Capnocytophaga ochracea*  *Capnocytophaga sputigena*  *Eikenella corrodens* |
| Orange | *Camphylobacter rectus*  *Camphylobacter gracilis*  *Camphylobacter showae*  *Eubacterium nodatum*  *Fusobacterium nucleatum nucleatum*  *Fusobacterium nucleatum polymorphum*  *Fusobacterium nucleatum vincentii*  *Fusobacterium periodonticum*  *Prevotella intermedia*  *Peptostreptococcus micros*  *Prevotella nigrescens*  *Streptococcus constellatus* |
| Red | *Porphyromonas gingivalis*  *Tannerella. forsythia*  *Treponema denticola* |
| White (other) | *Eubacterium saburreum*  *Gemella morbillorum*  *Leptotrichia buccalis*  *Neisseria mucosa*  *Cutibacterium acnes*  *Prevotella melaninogenica*  *Streptococcus anginosus*  *Selenomonas noxia*  *Treponema socranskii* |

**Appendix S11.** Mean values of Socransky complexes ^9^ before and at different time-points after NSPT; red/horizontal lines: red complex, white: other bacteria, blue: *actinomyces species*, purple/dotted: purple complex, yellow/checkerboard: yellow complex, green/vertical lines: green complex and orange/diagonal lines: orange complex; a decrease of red complex bacteria is observed after NSPT, which is maintained up to 12-month follow-up. The decrease in disease associated species (red complex bacteria) is accompanied by an increase of health associated.

**Appendix S12. Forest Plots for checkerboard DNA-DNA hybridization technology studies**

***A. actinomycetemcomitans*, baseline – 3 months, subgroup analysis with and without adjunctive therapy**

**
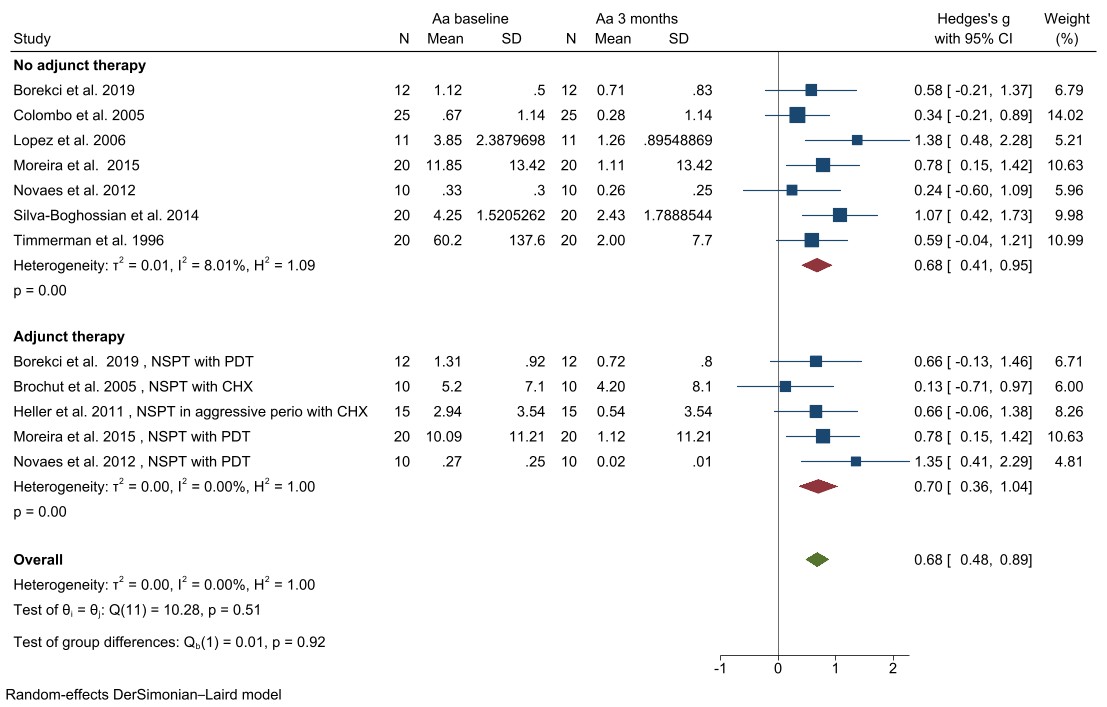
**

***P. gingivalis,* baseline – 3 months, subgroup analysis with and without adjunctive therapy**

**
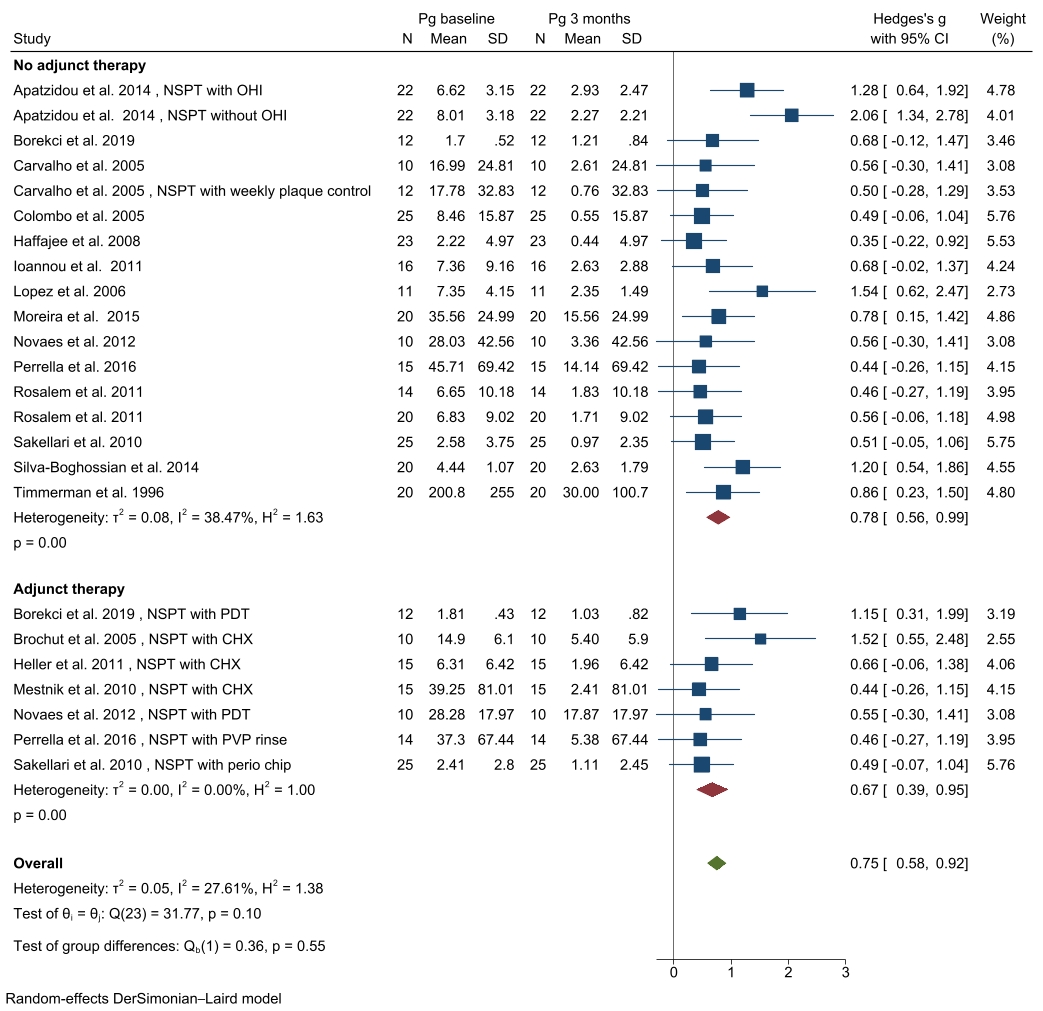
**

***P. gingivalis,* baseline – 6 months, subgroup analysis with and without adjunctive therapy**

**
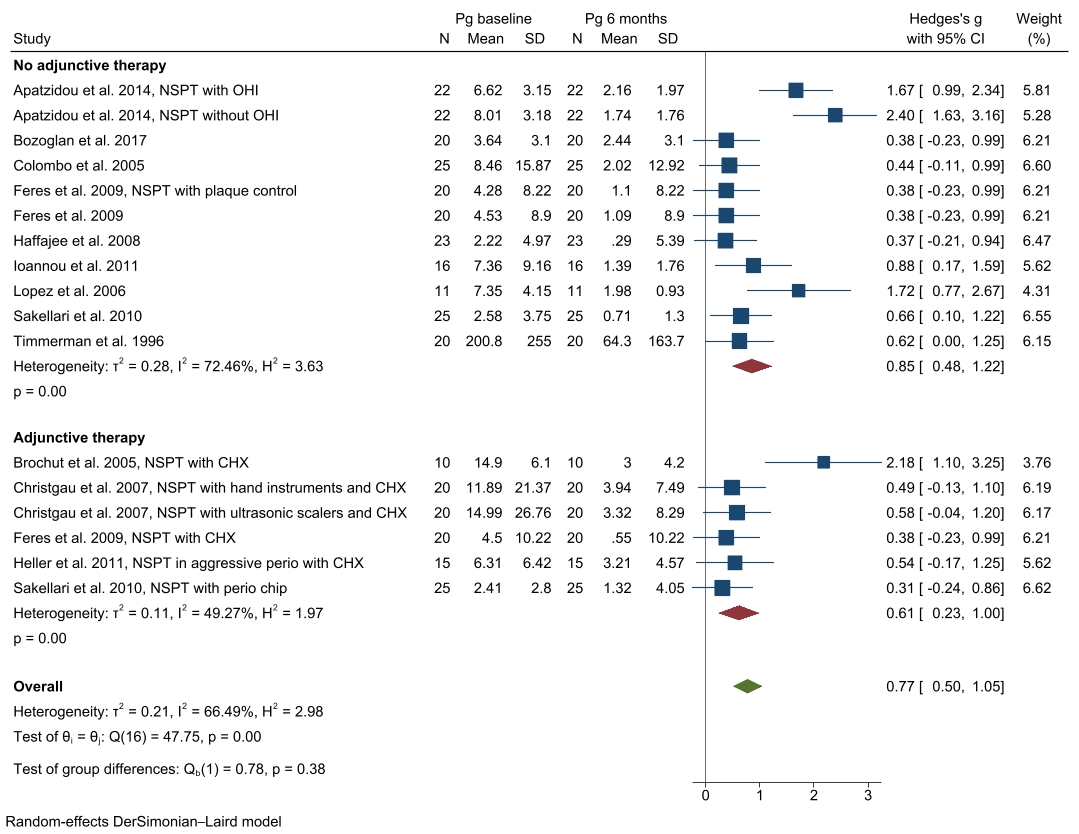
**

***T. forsythia,* baseline – 3 months, subgroup analysis with and without adjunctive therapy**

**
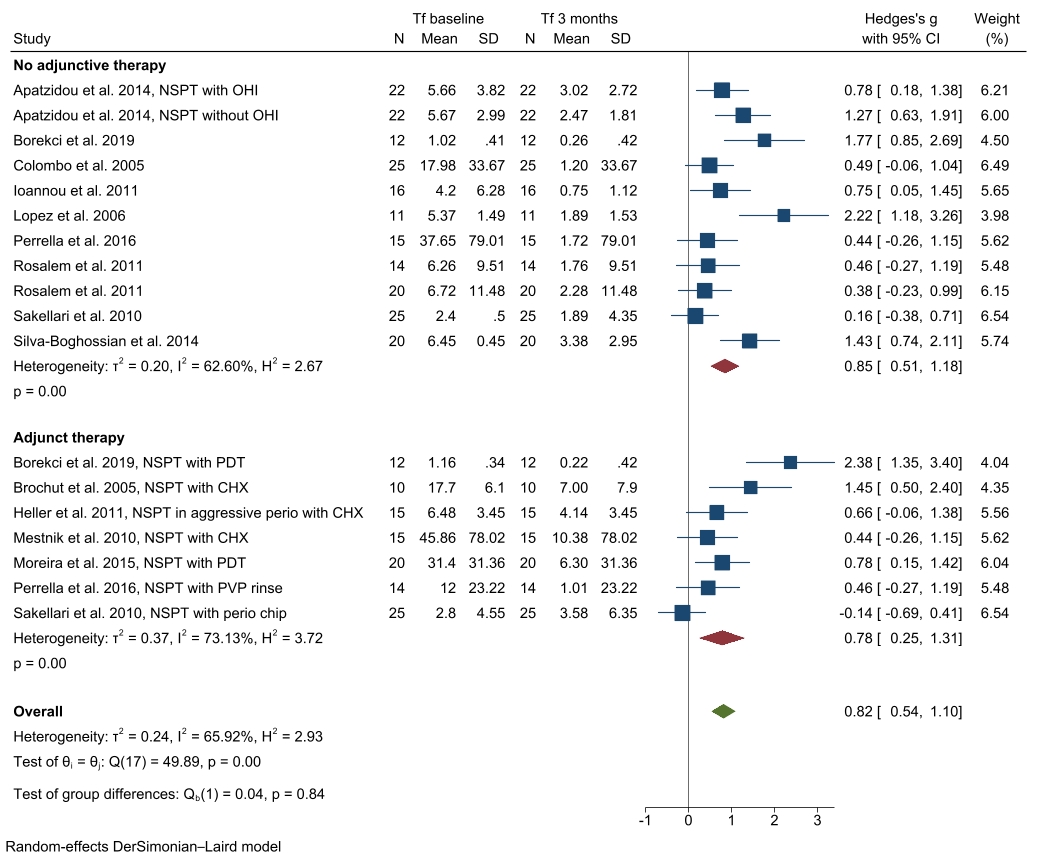
**

***T. forsythia,* baseline – 6 months, subgroup analysis with and without adjunctive therapy**

**
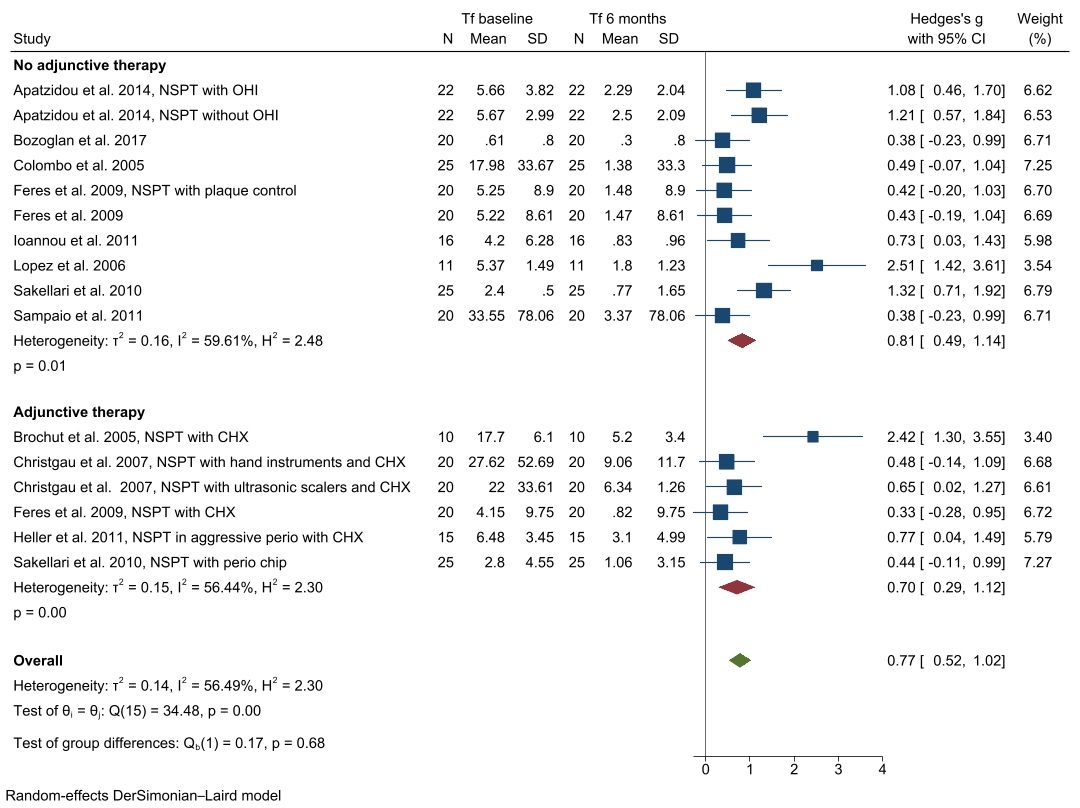
**

***T. denticola*, baseline – 3 months, without adjunctive therapy**

**
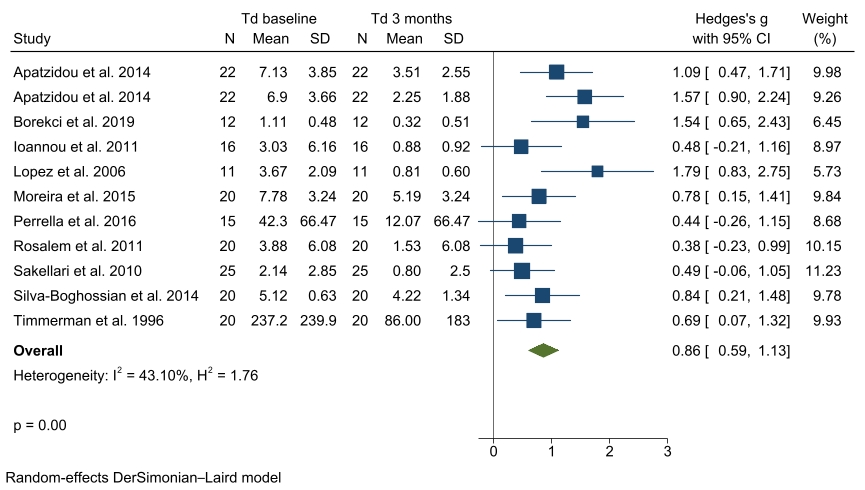
**

***T. denticola*, baseline – 6 months, subgroup analysis with and without adjunctive therapy**

**
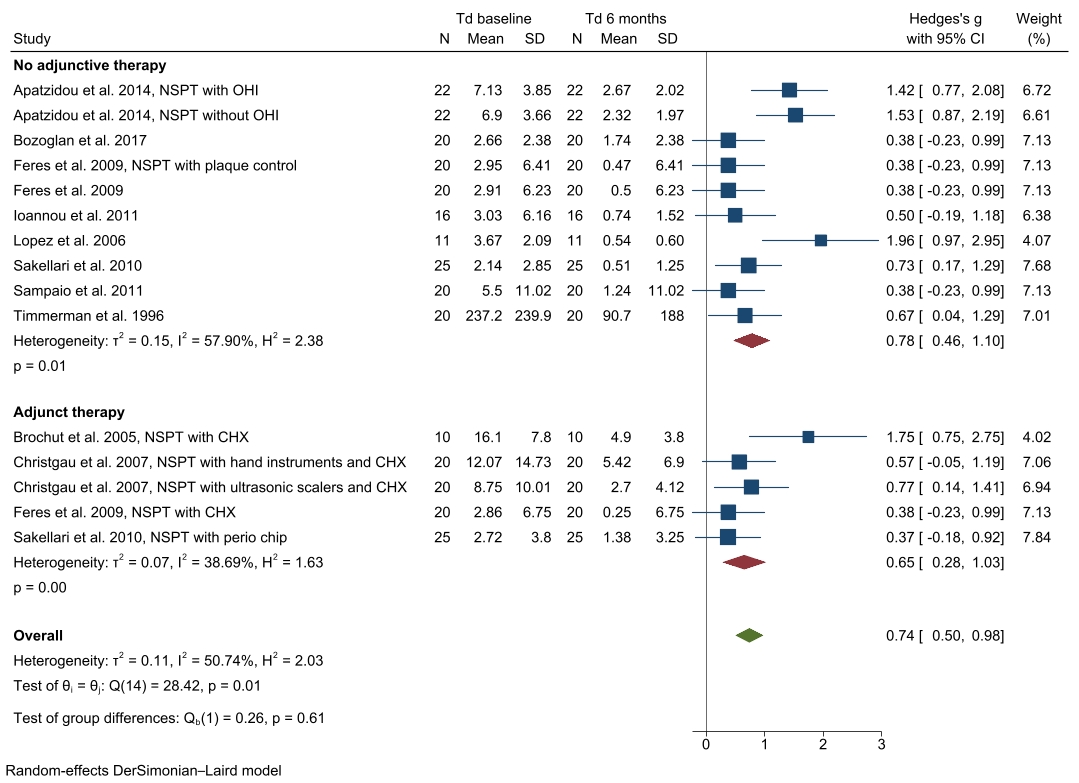
**

***E. nodadum*, baseline – 3 months, without adjunctive therapy**

**
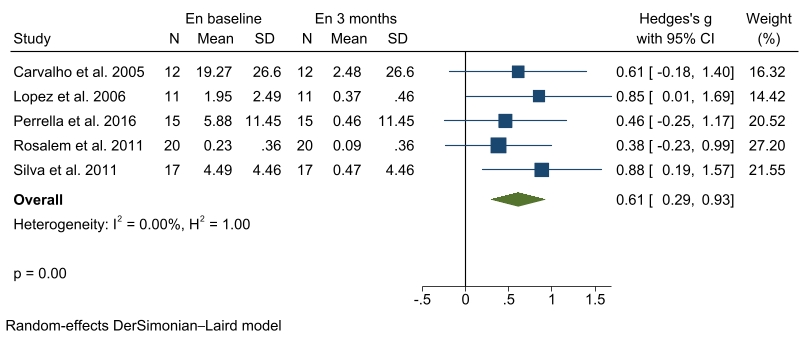
**

***P. intermedia,* baseline – 3 months, without adjunctive therapy**

**
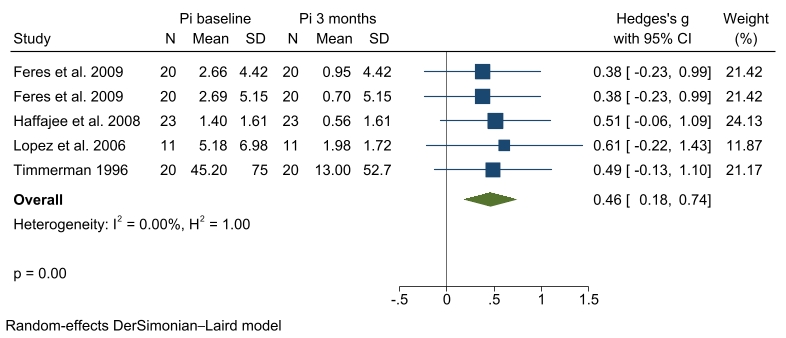
**

***C. rectus*, baseline – 6 months, without adjunctive therapy**

**
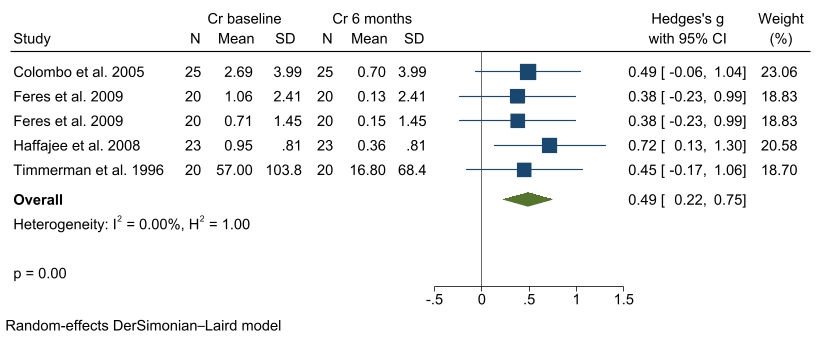
**

**Appendix S13: Forest plots, the influence of supportive periodontal care (SPC) on the effect sizes in checkerboard DNA-DNA hybridization technology studies**

***P. gingivalis, baseline – 6 months with and without SPC***


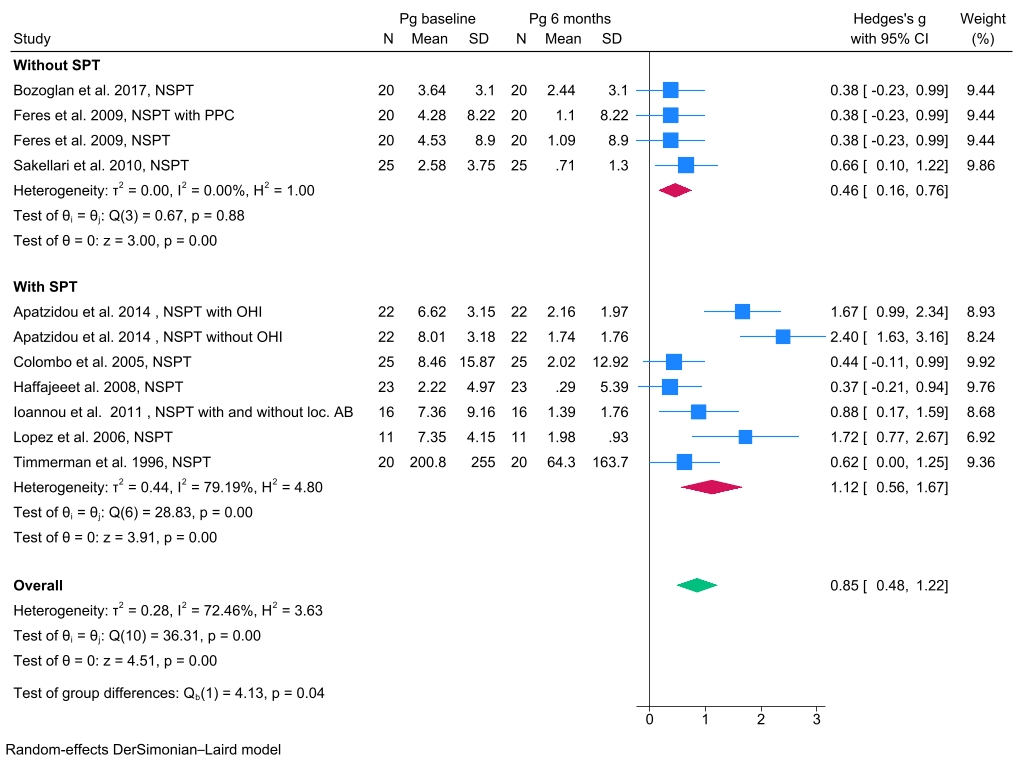


***T. denticola, baseline – 6 months with and without SPC***


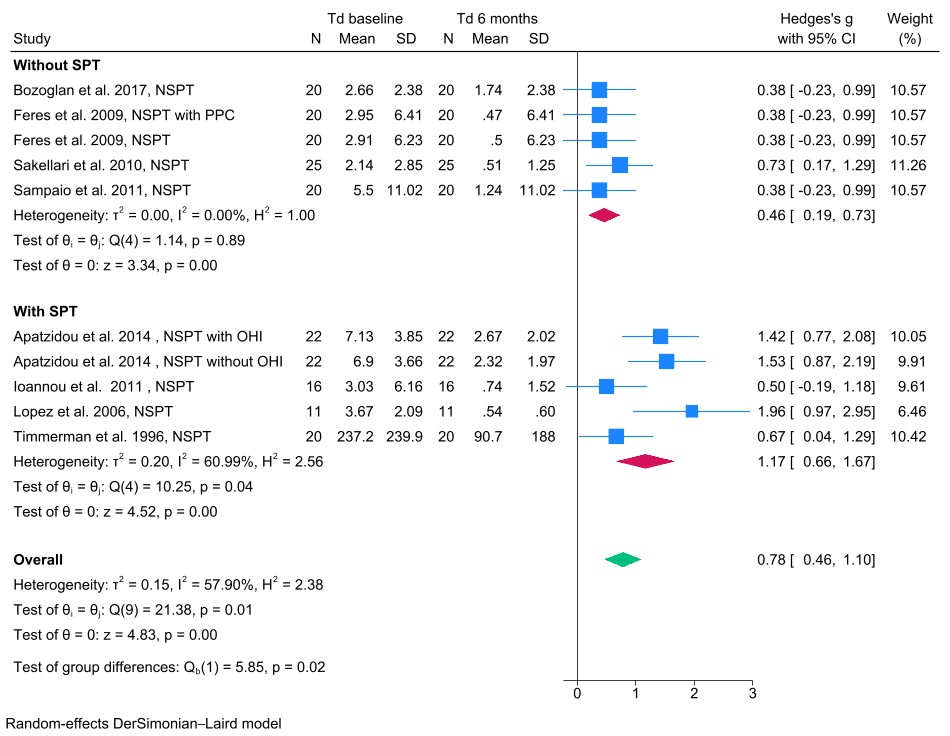


***T. forsythia, baseline – 6 months with and without SPC***


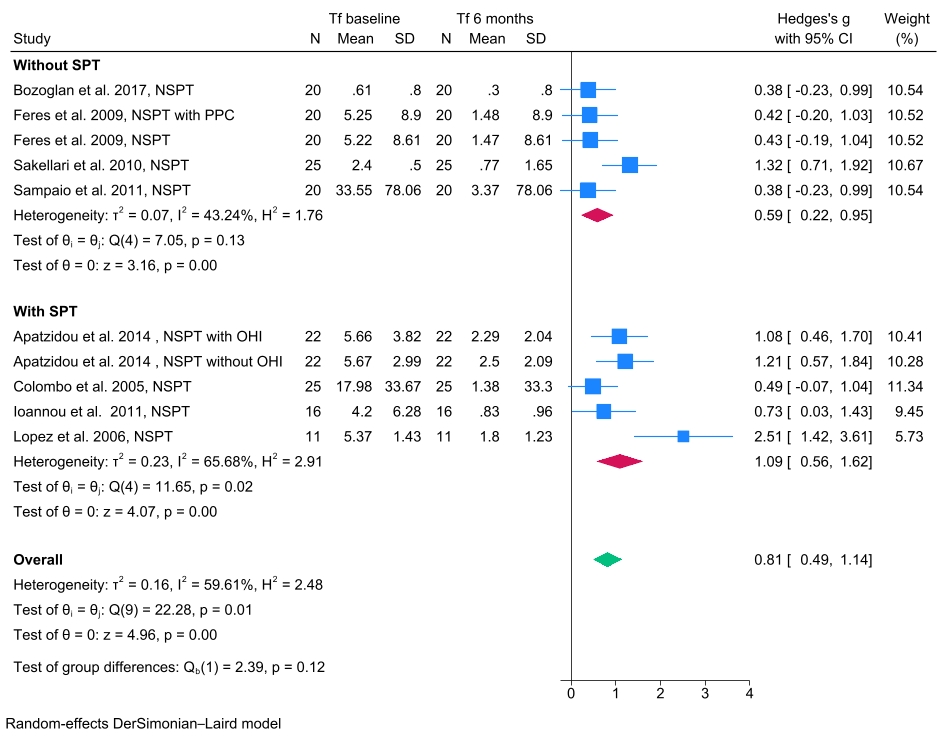


**Appendix S14**. Primary outcome reporting of real-time quantitative qPCR studies (n=30)

| **Authors, year** | **Sampling site** | **Method** | **Pooled/ SS** | **Bacterial species** | **Variability** | **X** |
| --- | --- | --- | --- | --- | --- | --- |
| Bergamaschi et al. 2016 | 5 sites PPD > 6mm at BL | PP | Pooled | *Aa, Pg* and *Tf* | SE |  |
| Buzinin et al. 2014 | Deepest site in each quadrant | PP | Pooled | *Aa, Pg* and *Tf* | SD |  |
| Chitsazi et al. 2014 | Deepest site test and control tooth | PP | SS | *Aa* | None | (a) |
| Cirino et al. 2019 | At random 2 moderate sites PPD>5-6 mm and 2 deep sites PPD>7 mm | PP | SS | *Aa* and *Pg* | CI | (b) |
| Cortelli et al. 2015 | Mesial first molar and central incisor | PP | Pooled | *Aa, Td, Pg, Tf, So* and *An* | P | (a) |
| Cosgarea et al. 2019 | 4 deepest sites per quadrant | PP | Pooled | *Aa, Td, Pg* and *Tf* | CI |  |
| Del Peloso Ribeiro et al. 2008 | 1 site PPD ≤ 5 mm and 1 site PPD ≤ 7 mm at baseline | PP | SS | *Aa, Pg* and *Tf* | SD |  |
| Do Vale et al. 2016 | 1 site PPD ≤ 5 mm and 1 site PPD ≤ 7 mm at baseline | PP | SS | *Aa* and *Pg* | CI |  |
| Eick et al. 2013 | Deepest sites premolar and molar | PP | Pooled | *Aa, Td, Pg* and *Tf* | None | (a) |
| Fonseca et al. 2015 | 2 sites per quadrant PPD ≤ 4 mm | PP | Pooled | *Aa, Td, Pg* and *Tf* | SD |  |
| Grzech-Lesniak et al. 2018 | No information | PP | N/I | *Aa, Td, Pg, Tf, Pi, Cg, Fn* and *En* | P | (b) |
| Guentsch et al. 2008 | 3 sites PPD ≤ 5 mm | PP | N/I | *Aa, Td, Pg* and *Tf* | P | (b) |
| Han et al. 2012 | 2 sites single rooted tooth PPD ≤ 6 mm | PP | SS | *Aa, Td, Pg* and *Tf* | SD |  |
| Hayakume et al. 2013 | 1 site PPD ≤ 4 mm | PP | SS | *Pg* and *Tf* | SD | (d) |
| Jervoe-Storm et al. 2007 | Deepest site of single rooted, PPD ≤ 5 mm | Both | SS | *Aa, Td, Pg* and *Tf* | CI | (b) |
| Liu et al. 2013 | Mesiobuccal sites of each involved tooth | PP | Pooled | *Aa, Pg* and *Tf* | P | (b) |
| Luchesi et al. 2013 | Furcation site | PP | SS | *Aa, Td, Pg* and *Tf* | SE |  |
| Park et al. 2018 | Site(s) with deepest PPD | Curette | SS | *Aa, Td, Pg, Tf, Pi, Fn, Cr* and *Ec* | SD |  |
| Peralta et al. 2020 | Mesial first molar and central incisor | Both | Pooled | *Aa, Td, Pg and Tf* | SE |  |
| Pulikkotil et al. 2016 | Random selection of sites | PP | Pooled | *Aa* | SD |  |
| Ramiro et al. 2018 | 3 sites PPD ≤ 5 mm | Curette | SS | Archaea | SD | (c) |
| Saglam et al. 2013 | 3 sites single rooted teeth PPD ≤ 5 mm | Curette | SS | *Td, Pg* and *Tf* | SD |  |
| Saglam et al. 2017 | 3 sites single rooted teeth PPD ≤ 5 mm | Curette | SS | *Td, Pg* and *Tf* | SD |  |
| Soeroso et al. 2017 | No information | N/I | N/I | *Td, Pg* and *Tf* | SD |  |
| Spooner et al. 2016 | Selected sites | PP | N/I | *Td, Pg* and *Tf* | P | (d) |
| Swierkot et al. 2009 | 4 deepest sites | PP | Pooled | *Aa* and *Pg* | None | (a) |
| Tanaka et al. 2015 | 2 sites PPD ≤ 4 mm and FMBS | PP | SS | *Td, Pg* and *Tf* | CI |  |
| Teughels et al. 2013 | Deepest sites single rooted teeth each quadrant | Curette | Pooled | *Aa, Pg* and *Tf* | SD |  |
| Theodoro et al. 2018 | Sites PPD ≤ 5 mm and PPD ≤ 7 mm at baseline | PP | SS | *Pg, Pi* and *Pn* | SE |  |
| Zengin Celik et al. 2019 | Deepest sites single rooted teeth each quadrant | PP | Pooled | *Td, Pg* and *Tf* | None | (a) |
| PP. paper point, SS: site specific, N/I. no information, *Aa: Aggregatibacter actinomycetemcomitans, An: Actinomyces naeslundii, Cg: Capnocytophaga gingivalis, Cr: Camphylobacter rectus, Ec: Eikenella corrodens, Fn: Fusobacterium nucleatum, Pi: Prevotella intermedia, Pg: Porphyromonas gingivalis, Pm: Peptostreptococcus micros: Pn: Prevotella nigrescens, Td: Treponema denticola, Tf: Tannerella forsythia, So: Streptococcus oralis*, SD: standard deviation, P: p-value, SE: standard error, CI: confidence interval, X: reason for exclusion from meta-analysis, (a): no measure of variation, (b): no mean values, (c): species, (d): time-points | | | | | | |

**Appendix S15.** Microbiological findings of **real-time** **quantitative PCR studies:** (A) non-smokers (blue) versus smokers (yellow) (B) chronic (blue) versus aggressive (yellow) periodontitis, (C) without SPC (blue) versus with SPC (yellow) and (D) without adjunctive therapy (blue) versus with adjunctive therapy (yellow). Turquoise indicates a decrease in the mean counts, grey no changes in the mean counts, and orange an increase in mean counts of subgingival bacteria after NSPT. Black indicates that microbiological data is not reported. * Indicates results that were reported as statistically significant (p-value ≤ 0.05). M: months, bacterial abbreviations: *An: A. naeslundii, So: S. oralis, Aa: A. actinomycetemcomitans a, Cg: C. gingivalis, Ec: E. corrodens, Cg: C. gracilis, Cr: C. rectus, En: E. nodatum, Fn: F. n. nucleatum, Pi: P. intermedia, P.m: P. micros, Pn: P. nigrescens, Tf: T. forsythia, Pg: P. gingivalis, Td: T. denticola, BPB: black pigmented bacteria*

**Appendix S16. Forest Plots for real-time quantitative qPCR**

***A. actinomycetemcomitans*, baseline – 3 months, subgroup analysis with and without adjunctive therapy**


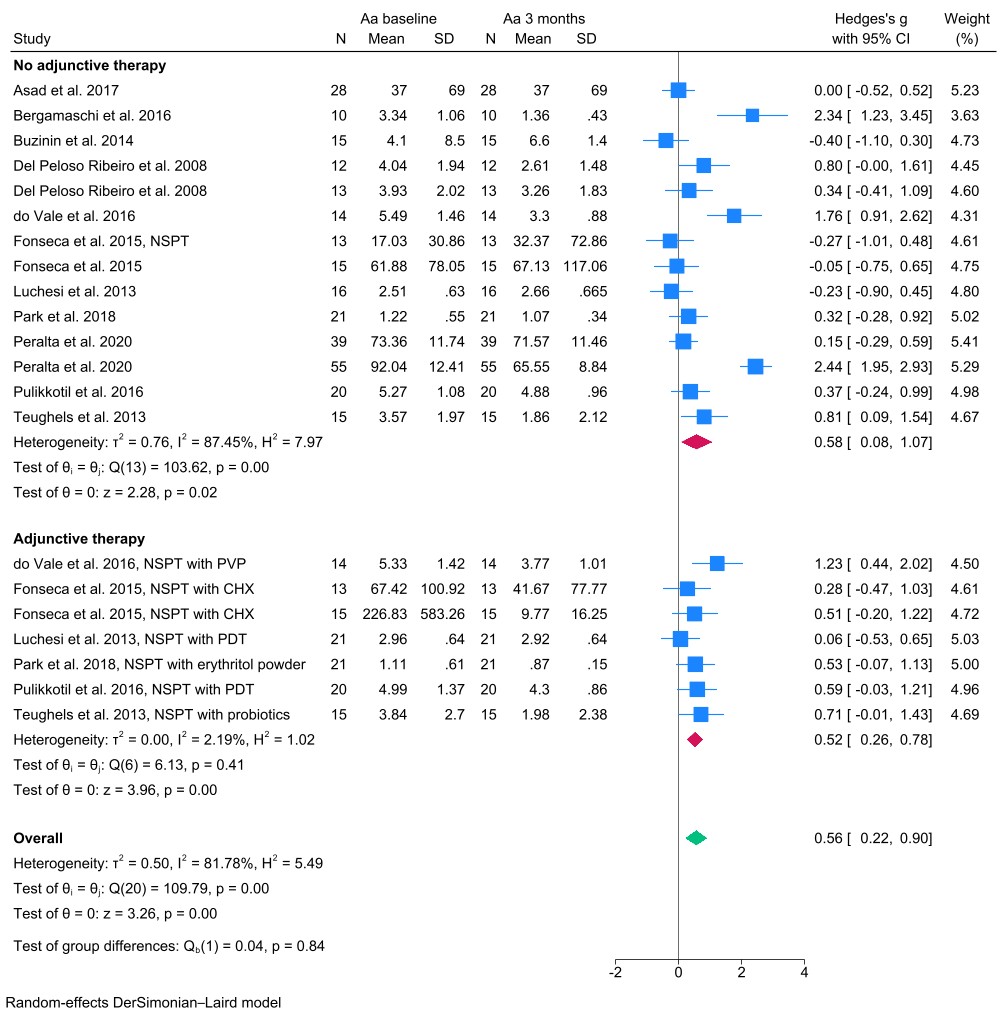


***A. actinomycetemcomitans*, baseline – 6 months, without adjunctive therapy**


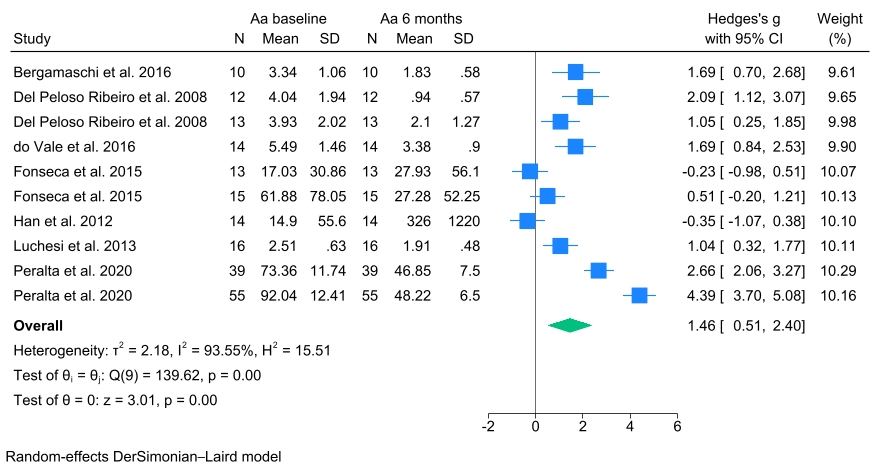


***P. gingivalis,* baseline – 1.5 months, subgroup analysis with and without adjunctive therapy**


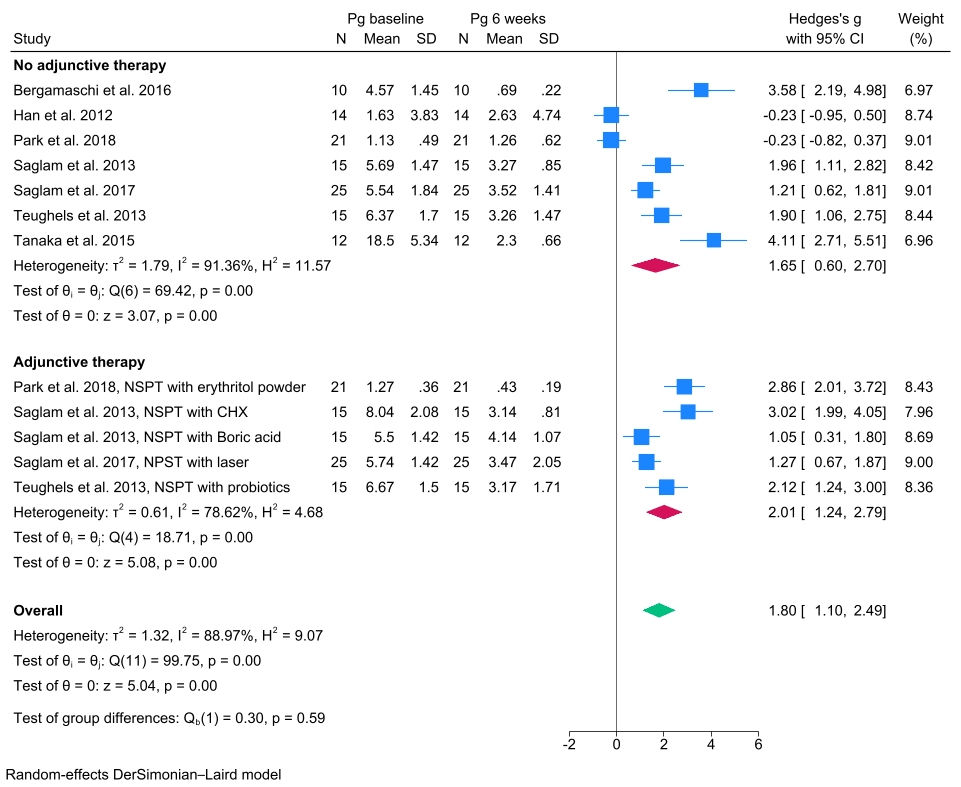


***P. gingivalis,* baseline – 3 months, subgroup analysis with and without adjunctive therapy**


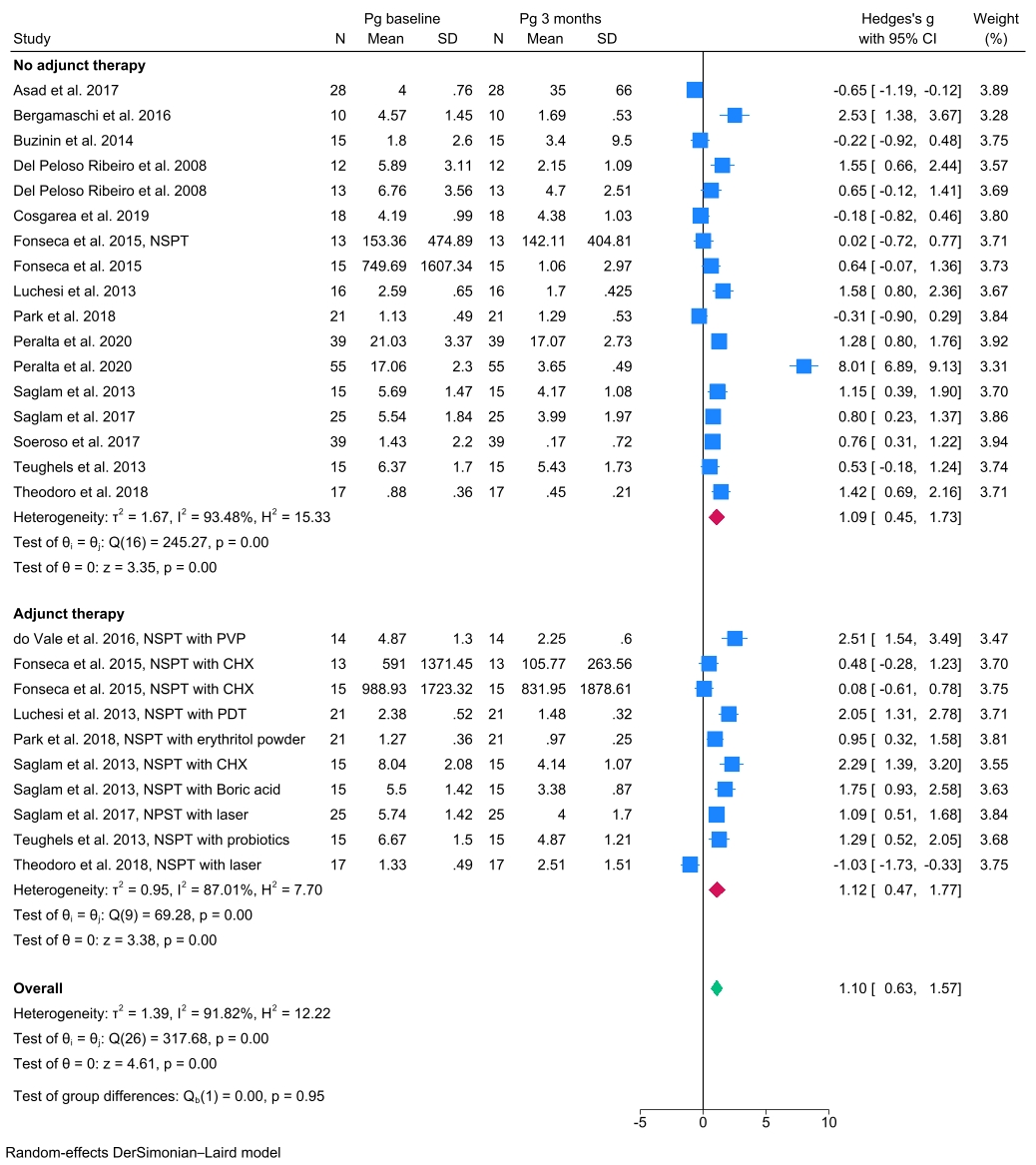


***P. gingivalis,* baseline – 6 months, subgroup analysis with and without adjunctive therapy**


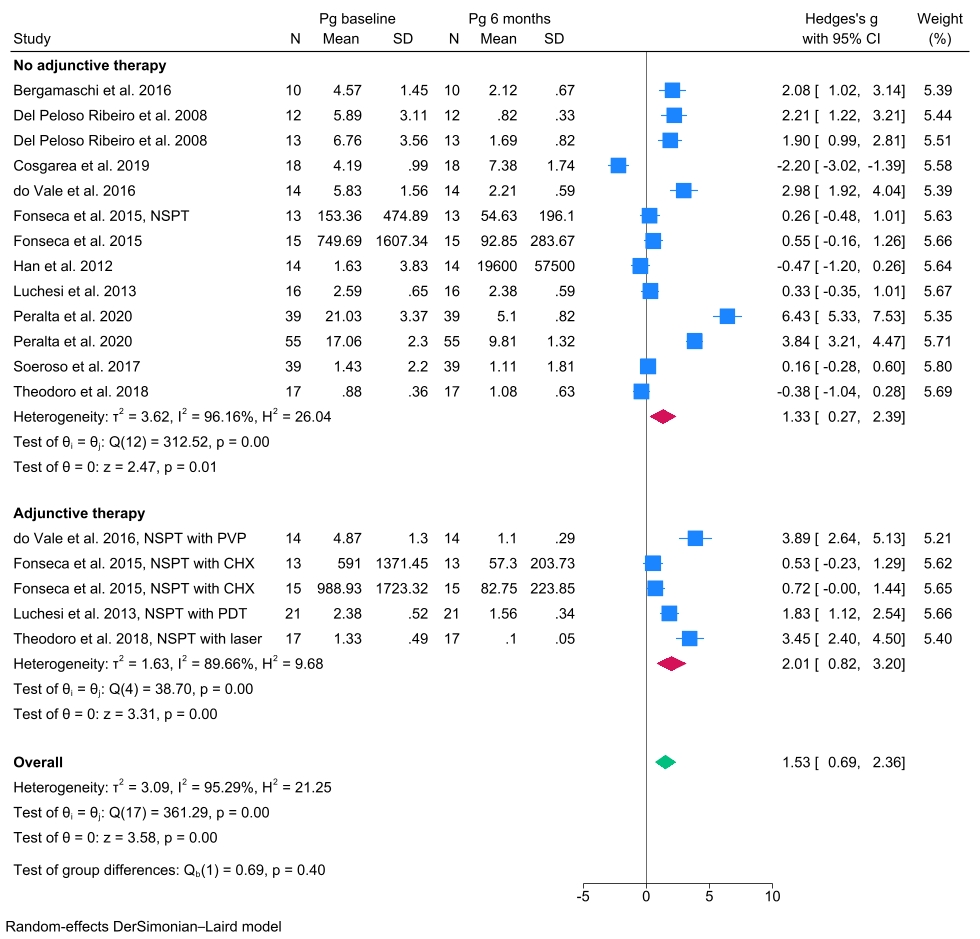


***T. forsythia,* baseline – 1.5 months, subgroup analysis with and without adjunctive therapy**


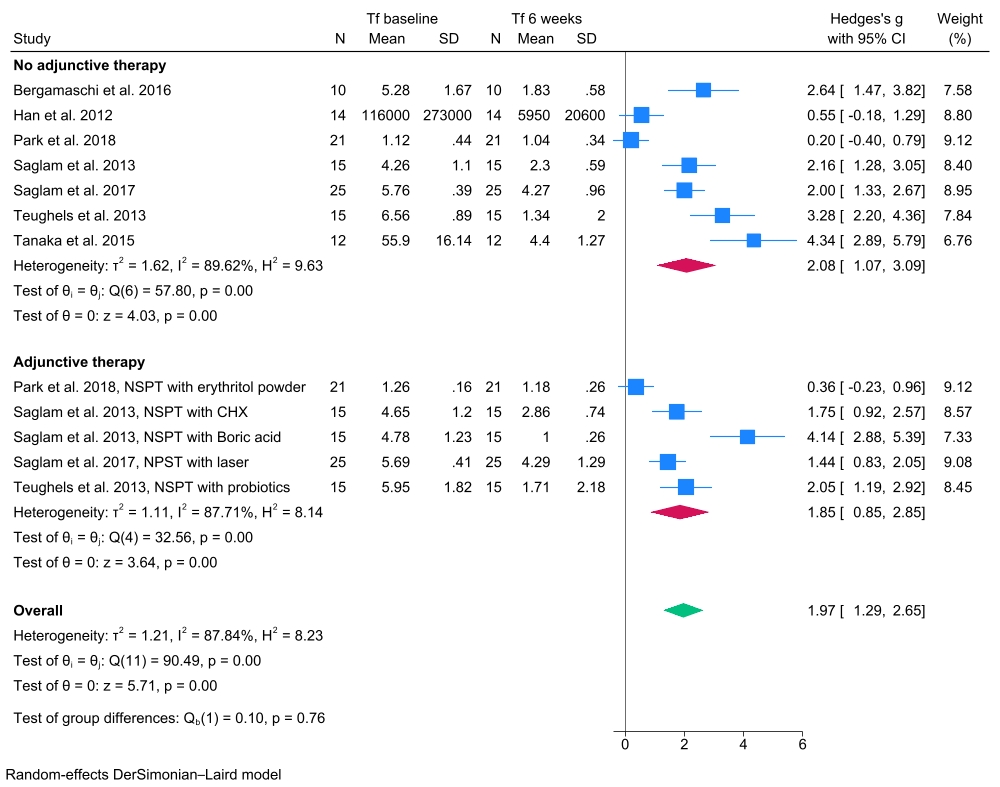


***T. forsythia,* baseline – 3 months, subgroup analysis with and without adjunctive therapy**


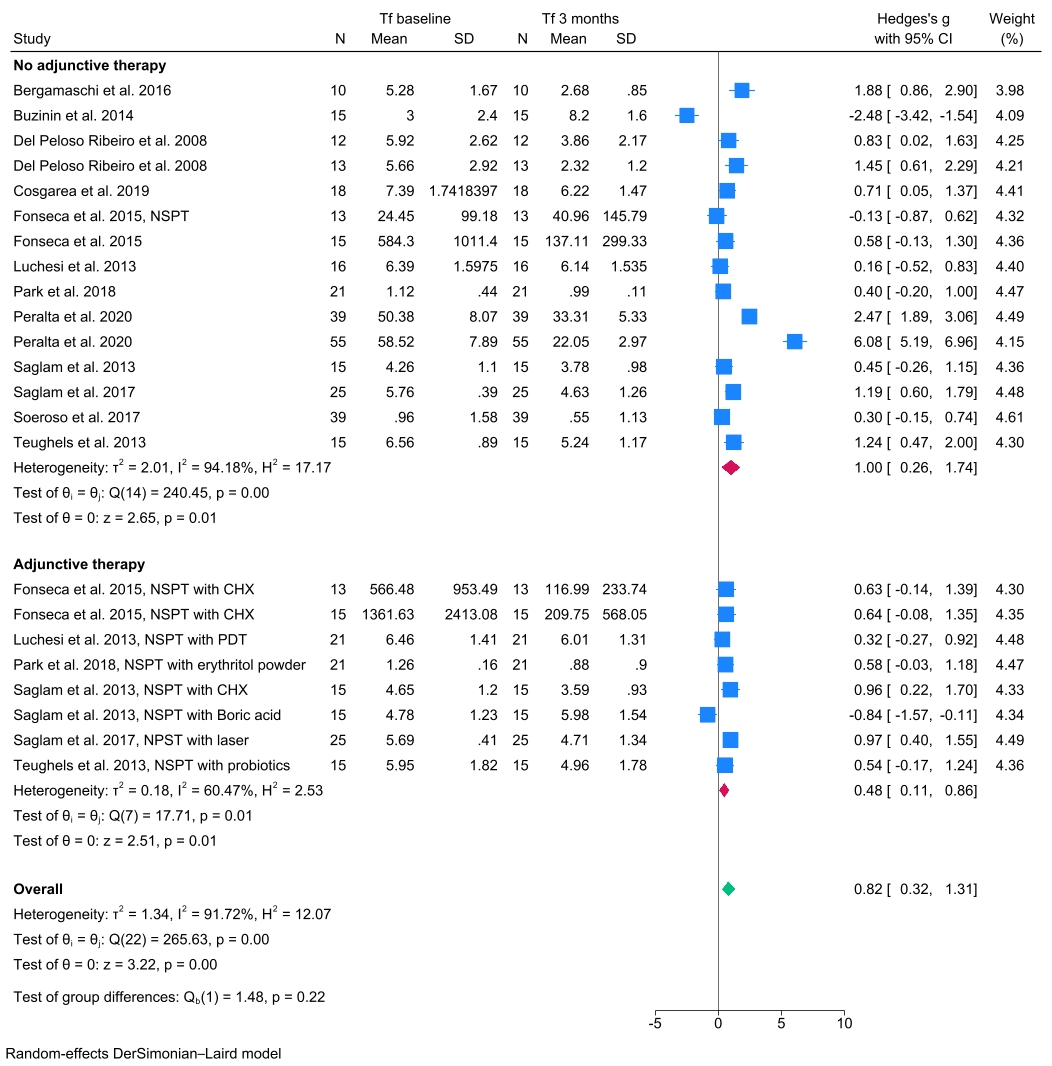


***T. forsythia,* baseline – 6 months, without adjunctive therapy**

**
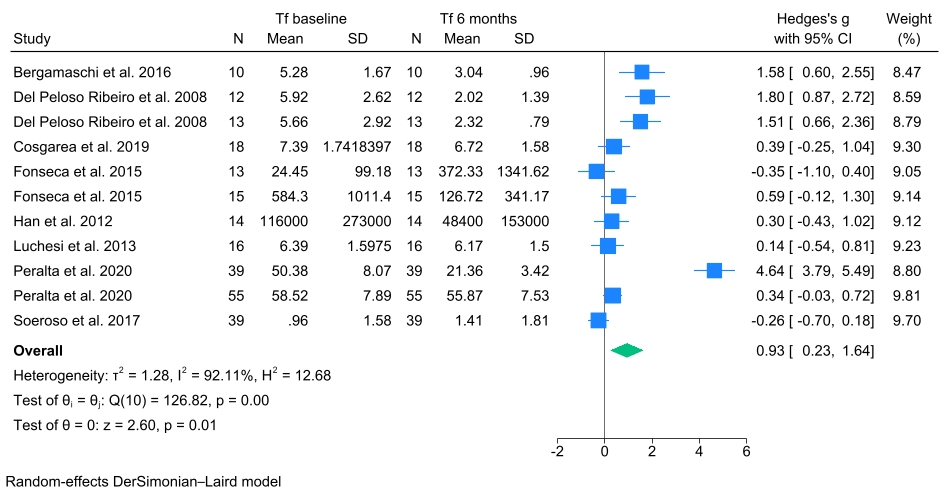
**

***T. denticola*, baseline – 3 months, subgroup analysis with and without adjunctive therapy**


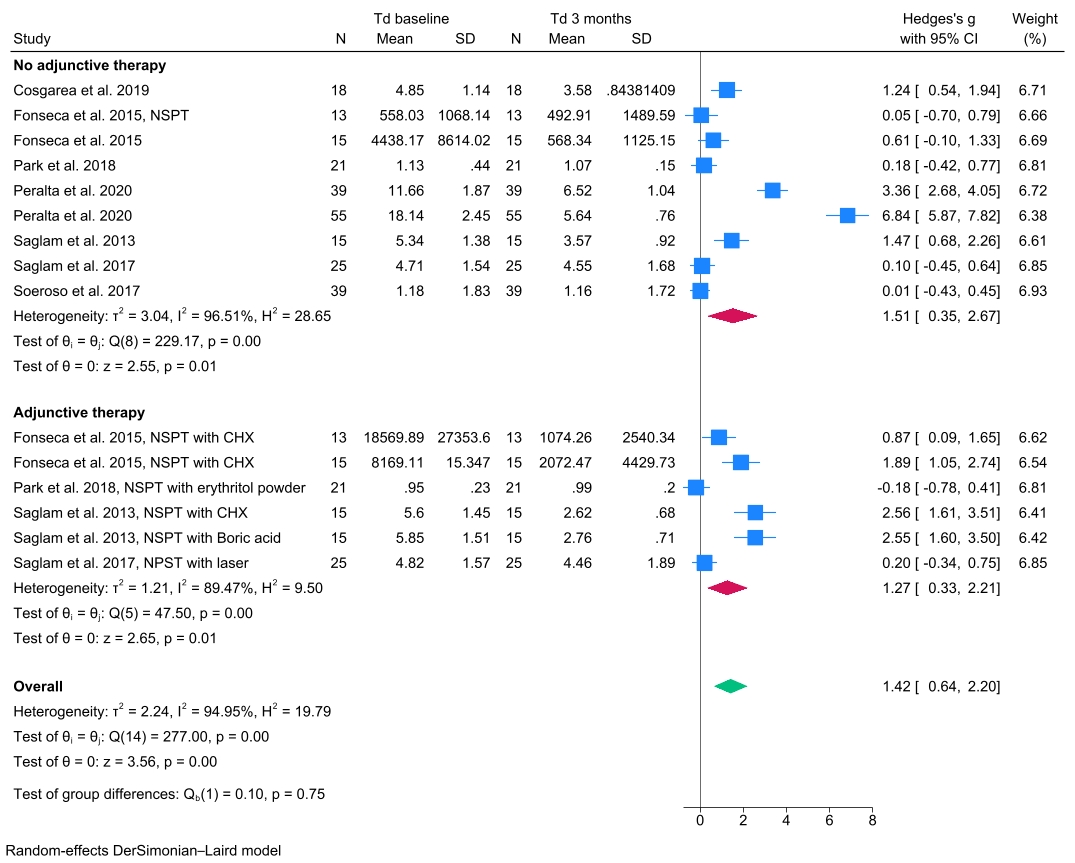


***T. denticola*, baseline – 6 months, without adjunctive therapy**


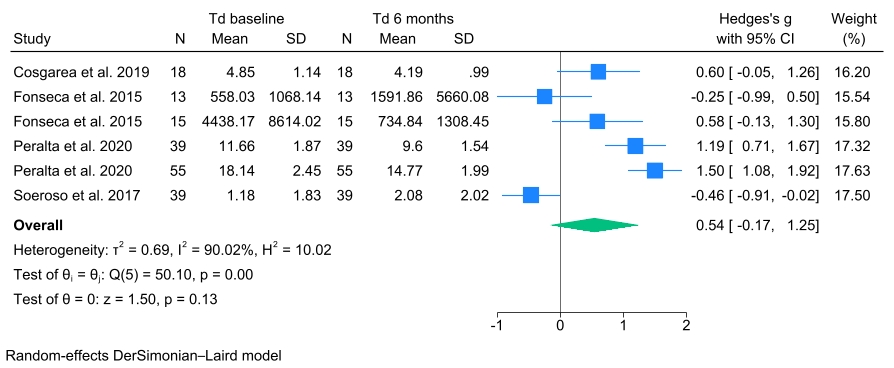


**Appendix S17.** Primary outcome reporting of bacterial culture studies (n = 36)

| **Author/ year** | **Sampling site** | **Sampling method** | **Pooled/ SS** | **Culture plates** | **Inc. (d)** | **Species** | **Variability** | **X** |
| --- | --- | --- | --- | --- | --- | --- | --- | --- |
| Ali et al. 1992 | 7-10 sites per patients | PP, VMGA | Pooled | BA, CAP, TSBV | 5 | *Aa, Pg, Pi & Capnocytophaga spp.* | SE |  |
| Annaji et al. 2016 | Deepest site per quadrant | Curette, RTF | SS | BA & TSBV | 3 | *Aa* & *BPB* | SE |  |
| Ardila et al. 2015 | 6 deepest sites | PP, VMGA | Pooled | BA & TSBV | 7/ 4 | *Aa, Pg* & *Tf* | None | (a) |
| Ardila et al. 2017 | 6 deepest sites | PP, VMGA | Pooled | BA & TSBV | 7/ 4 | *Aa* | None | (e) |
| Berglundh et al. 1998 | 3 deepest interproximal sites | PP, VMGA | Pooled | BA & TSBV | 7.00 | *Aa, Pg* & *Pi* | None | (a) |
| Bhatia et al. 2014 | No information | Curette, TM | N/I | N/I | N/I | *Pg, Pi, Fn* & *Capnocytophagia spp*. | SD |  |
| Bizzarro et al. 2016_2 | Deepest site per quadrant | PP, RTF | Pooled | BA & TSBV | 14/ 7 | *Aa, Pg, Pi, Fn, Pm, Tf* & *Cr* | SD |  |
| De Micheli et al. 2011 | 2 sites with PPD ≥ 5 mm | PP, VMGA | SS | BA & TSBV | 7/ 3 | *Aa, Pg* & *P*i | SD |  |
| De Soete et al. 2005 | Reference pockets | PP, RTF | Pooled | TYCSB & Rogosa | N/I | *S. mutans* & *lactobacilli* | None | (a) |
| Dhaliwal et al. 2017 | Experimental sites | PP, RTF | SS | BA & dentaid | 3/4 | *Aa, Pg* & *Pi* | SD |  |
| Dominguez et al. 2010 | For deep sites | PP, RTF | SS | BA | 7-14/ 2-3 | *Aa, Pg, Pi, Pm, Fn, Cr, Ec, Tf, En* & *Capnocytophagia spp.* | SD |  |
| Euzebio Alves et al. 2013 | No information | PP, VMGA | Pooled | TSBV | 3 | *Aa, Pg* & *Pi* | None | (a) |
| Gibson et al. 1994 | No information | PP, saline | N/I | WC | 7-14 | *Anaerobes* | None | (a) |
| Gomez et al. 2011 | 4 deep sites | PP, RTF | SS | BA | 7-14 | *Aa, Pg, Pi, Pm, Fn, Cr, Ec, Tf, En* & *Capnocytophagia spp.* | SD |  |
| Lombardo et al. 2015 | No information | Curette, TM | SS | CB & Schaedler KV | 2 | Aerobic & anaerobic | Box plot | (b) |
| Manikandan et al. 2016 | deepest pocket | Curette, TM | SS | ETS | 7-10 | Anaerobic | Values | (c) |
| Martande et al. 2016 | Deepest site per quadrant | PP, Ringer | Pooled | TSBV | 3.00 | *Aa* | None | (a) |
| Moeintaghavi et al. 2007 | No information | Curette, RTF | N/I | Sheep agar & CBRSA | N/I | *Aa, Pg* & *Pi* | SD |  |
| Morales et al. 2018 | 4 sites with PPD ≥ 4 mm | PP, RTF | SS | BA | 14 | *Aa, Pg* & *Tf* | SD |  |
| Pradeep et al. 2014 | Deepest site per quadrant | PP, Ringer | Pooled | TSBV | 3 | *Aa* | None | (a) |
| Preber et al. 1995 | 1 experimental site | PP, VMGA | SS | TSBV, BA | 3-5/10 | *Aa, Pg* & *Pi* | None | (a) |
| Quirynen et al. 1995 | 3 deepest pockets of single and multirooted tooth | PP, RTF | Pooled | TSBV, BA | 3/ 5 | *Pg, Pi, Fn, Pm* & *En* | SD |  |
| Quirynen et al. 1999 | 4 deep sites | PP, RTF | Pooled | TSBV, BA & Hammond | 3/ 5 | *BPB* | SD | (c) |
| Quirynen et al. 2000 | 4 deep sites | PP, RTF | Pooled | TSBV, BA & Hammond | 3/ 5 | Total number of bacteria | None | (c) |
| Renvert et al. 1998 | 1 experimental site PPD ≥ 6 mm | PP, VMGA | SS | TSBV, BA | 5/ 7-9 | *Aa, Pg* & *Pi/Pn* | SD |  |
| Roman-Torres et al. 2018 | Sites with greater probing depth | PP, Ringer | Pooled | BA | 10 | *Pg* & *Pi* | P value | (b) |
| Rooney et al. 2002 | 1 site per quadrant with PPD ≥ 6 mm | Curette, RTF | Pooled | FAA, BA & TSBV | 7/ 7/ 5 | *Aa, Pg* & *Pi* | None | (a) |
| Sanz-Sanchez et al. 2016 | Deepest site per quadrant | PP, RTF | Pooled | Dentaid-1, BA | 3/ 7-14 | *Aa, Pg, Pi/Pn, Pm, Fn, Cr* & *Tf* | None | (a) |
| Sefton et al. 1996 | 1 site per quadrant with PPD ≥ 6 mm | PP, FAB | SS | FAA, TSBV & MSA | 5/ 3/ 5 | *Aa, Pg, Pi*, *anaerobes,* *spirochaetes* | None | (a) |
| Sreedhar et al. 2015 | Deepest site per quadrant | Curette, RTF | SS | TSBV, BA | 3 | *Aa* & *BPB* | SD |  |
| Suchetha et al. 2013 | No information | PP, RCMM | N/I | TSBV & ETS | 2 | *Aerobic* & *anaerobic* | SD | (c) |
| Suryaprasanna et al. 2018 | Deep periodontal pocket | Curette, RCMM | SS | BA, chocolate agar | 3 | *Aa* & *Pg* | None | (a) |
| Tekce et al. 2015 | 2 approximal sites with PPD 5-7 mm per quadrant | PP, PBS | Pooled | TSBV, two plates | 7-10/ 4 | *Anaerobes* | SD | (c) |
| Winkel et al. 2001 | Deepest site with FMBS per quadrant | PP, RTF | Pooled | BA & TSBV | 14/ 5 | *Aa, Pg, Pi, Pm, Fn* & *Tf* | SD |  |
| Yilmaz et al. 2012 | No information | PP, PBS | N/I | TSBV, two plates | 7-10/ 4 | Obligate anaerobes | SD | (c) |
| Yilmaz et al. 2013 | 1 site per quadrant with PPD ≥ 5 mm | PP, PBS | Pooled | TSBV, two plates | 7-10/ 4 | Obligate anaerobes | SD | (c) |
| PP. paper point, N/I. no information, BA: Blood agar, CAP; Capnocytophaga plates, TSBV: Soy-serum-bacitracin-vancomycin, BBL: Brucella agar, WC: Wilkins-Chalgren agar, CB: Columbia blood agar, ETS: Enriched trypticase soya agar, CBRSA: Chocolate blood tripticase soy agar, FAA: Fastidious Anaerobe Agar, MSA: Mitis salivarius agar, PBS: phosphate-buffered saline, FAB: Fastidious Anaerobe Broth, RCMM: Robertson's cooked meat medium, TM: Thioglycollate medium, SS: site specific, BPB: Black pigmented bacteria, *Aa: Aggregatibacter actinomycetemcomitans, Cr: Camphylobacter rectus, Ec: Eikenella corrodens, Fn: Fusobacterium nucleatum, Pi: Prevotella intermedia, Pg: Porphyromonas gingivalis, Pm: Peptostreptococcus micros, Pn: Prevotella nigrescens, Tf: Tannerella forsythia*, BPB: black pigmented bacteria, SD: standard deviation, P: p-value, SE: standard error, CI: confidence interval, X: reason for exclusion from meta-analysis, (a): no measure of variation, (b): no mean values, (c): species, (d): time-points, (e): patients from previous study | | | | | | | | |

**Appendix S18.** Microbiological findings of **bacterial culture studies:** (A) non-smokers (blue) versus smokers (yellow) (B) chronic (blue) versus aggressive (yellow) periodontitis, (C) without SPC (blue) versus with SPC (yellow) and (D) without adjunctive therapy (blue) versus with adjunctive therapy (yellow). Turquoise indicates a decrease in the mean counts, grey no changes in the mean counts, and orange an increase in mean counts of subgingival bacteria after NSPT. Black indicates that microbiological data is not reported. * Indicates results that were reported as statistically significant (p-value ≤ 0.05). M: months, bacterial abbreviations: *BPB: black pigmented bacteria, Anaer.: anaerobic bacteria, Aer. Aerobic bacteria, Aa: A. actinomycetemcomitans, Ec: E. corrodens Cr: C. rectus, En: E. nodatum, Fn: F. nucleatum, P.m: P. micros, Pi: P. intermedia, Tf: T. forsythia, Pg: P. gingivalis, Cap: Capnocytophaga, Eu: Eubacterium*

**Appendix S19.** Longitudinal meta-analysis of bacterial culture studies comparing mean counts of *A. actinomycetemcomitans* at baseline and 3 months after NSPT. Squares on the left side of the vertical zero line indicate fewer bacteria found at baseline, squares on the right side of the vertical zero line indicate fewer bacteria found at 3 months. The green rhombus indicates the effect size. At 3 months after NSPT there was significantly lower mean counts of. *A. actinomycetemcomitans* compared to baseline (p-value = 0.00)


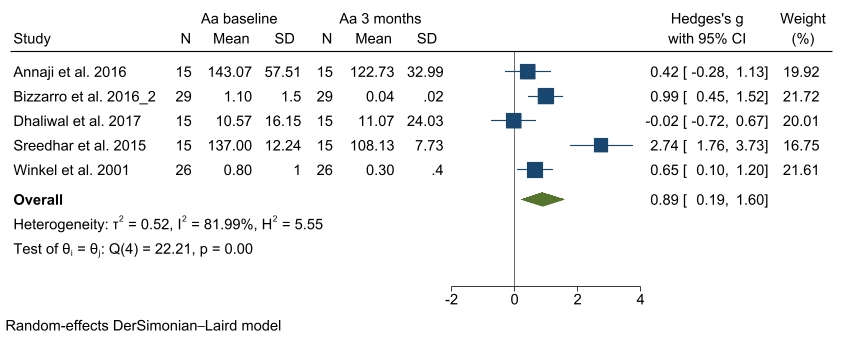


**Appendix S20.** Primary outcome reporting of 16S rRNA gene sequencing studies (n=8).

| **Author, year** | **Sampling site** | **Sampling method** | **Pooled/ SS** | **Power calculation** | **Species Richness** | **Genera** | **Diversity** |
| --- | --- | --- | --- | --- | --- | --- | --- |
| Belstrom et al. 2018 | 4 deepest sites | Curette | Pooled | Saliva alpha diversity | 507 | N/I | Shannon Index |
| Bizzarro et al. 2016 | N/I | N/I | Pooled | N/I | N/I | 195 | Shannon Index  PCA  Bray-Curtis |
| Hagenfeld et al. 2018 | 4 sites with PPD ≤ 6 mm | PP | Pooled | N/I | 360 | 87 | Shannon Index  Bray-Curtis |
| Izidoro et al. 2023 | N/I | Curette | N/I | N/I | N/I | N/I | Not reported |
| Liu et al. 2018 | 4 x 4 sites different locations | PP | Pooled | N/I | 307 | 147 | Shannon Index  PCoA |
| Lu et al. 2021 | Ramfjord teeth, bucco-mesial site | Curette | Pooled | Difference in genus *Porphyromonas* between BL and T1 | 334 | 111 | Chao 1  Shannon  PCoA  Rel. abundance  MCoON |
| Martelli et al. 2016 | 4 deepest sites | PP | Pooled | N/I | N/I | N/I | Not reported |
| Nie et al. 2024 | N/I | Curette | SS | PPD | N/I | N/I | Species  Richness  Shannon Index  Bray Curtis |
| N/I: no information, PP: paper point, SS: site specific, PPD: probing pocket depth, PCA: Principal Component Analysis, PCoA: Principal Coordinate Analysis, MCoON: Microbial co-occurrence networks | | | | | | | |

**Appendix S21.** Comparision of effect sizes between whole-mouth and split-mouth design studies

| **Species** |  | **Checkerboard DNA-DNA hybridization technology** | | **Real-time quantitative PCR** | |
| --- | --- | --- | --- | --- | --- |
|  |  | Whole mouth | Split mouth | Whole mouth | Split mouth |
| *A. actinomycetemcomitans* | N | 5 | 2 | 12 | 2 |
|  | Effect size | 0.72 | 0.68 | 0.62 | 0.35 |
|  | CI | 0.37 - 1.08 | 0.08 - 1.10 | 0.03 - 1.20 | -0.08 - 0.78 |
|  | p-value | NS | | NS | |
| *P. gingivalis* | N | 15 | 2 | 15 | 2 |
|  | Effect size | 0.79 | 0.7 | 0.85 | 0.25 |
|  | CI | 0.55 - 1.03 | 0.20 - 1.21 | -0.05 - 1.75 | -0.84 - 1.34 |
|  | p-value | NS | | NS | |
| *T. forsythia* | N |  |  | 13 | 2 |
|  | Effect size |  |  | 1.04 | 0.8 |
|  | CI |  |  | 0.16 - 1.91 | 0.02 - 1.58 |
|  | p-value |  |  | NS | |
| *T. denticola* | N |  |  | 7 | 2 |
|  | Effect size |  |  | 1.92 | 0.13 |
|  | CI |  |  | 0.40 - 3.44 | -0.27 - 0.54 |
|  | p-value |  |  | 0.03 | |
| N: number of studies which reported on the specific follow up time-point, CI: confidence interval, effect size: Hedges’s g, p-value test of group difference, NS. not significant | | | | | |

**Appendix S22. Secondary outcome reported across all four microbiological detection techniques (n=115)**

| **Author, year** | **Method** | **Plaque** | **Bleeding** | **PPD** | **CAL** |
| --- | --- | --- | --- | --- | --- |
| Ali et al. 1992 | Culture | No | FMBS | Yes | No |
| Annaji et al. 2016 | Culture | PI | FMBS/SBI | Yes | Yes |
| Apatzidou et al. 2014 | Checkerboard | FMPS | FMBS | Yes | Yes |
| Ardila et al. 2015 | Culture | FMPS | FMBS | Yes | Yes |
| Ardila et al. 2017 | Culture | FMPS | FMBS | Yes | Yes |
| Belstrom et al. 2018 | 16S | FMPS | FMBS | Yes | Yes |
| Bergamaschi et al. 2016 | qPCR | VPI | GBI | Yes | No |
| Berglundh et al. 1998 | Culture | PI | FMBS | Yes | Yes |
| Bhatia et al. 2014 | Culture | PI | SBI | Yes | Yes |
| Bizzarro et al. 2016 | 16S | FMPS | FMBS | Yes | Yes |
| Bizzarro et al. 2016_2 | Culture | FMPS | FMBS | Yes | Yes |
| Borekci et al. 2019 | Checkerboard | PI | GBI | Yes | Yes |
| Bozoglan et al. 2017 | Checkerboard | PI | FMBS | Yes | Yes |
| Brochut et al. 2005 | Checkerboard | PI | FMBS | Yes | No |
| Buzinin et al. 2014 | qPCR | FMPS | FMBS | Yes | Yes |
| Carvalho et al. 2005 | Checkerboard | FMPS | FMBS | Yes | Yes |
| Chitsazi et al. 2014 | qPCR | None | FMBS | Yes | Yes |
| Christgau et al. 2006 | Checkerboard | API | PBI | Yes | No |
| Christgau et al. 2007 | Checkerboard | API | PBI | Yes | No |
| Cirino et al. 2019 | qPCR | FMPS | FMBS | Yes | Yes |
| Colombo et al. 2005 | Checkerboard | FMPS | FMBS | Yes | Yes |
| Cortellini et al. 2015 | qPCR | PI | No | Yes | Yes |
| Cosgarea et al. 2019 | qPCR | FMPS | FMBS | Yes | Yes |
| Cugini et al. 2000 | Checkerboard | FMPS | FMBS | Yes | Yes |
| DelPelosoRibeiro et al. 2008 | qPCR | VPI | FMBS | Yes | No |
| deMeloSoares et al. 2019 | Checkerboard | FMPS | FMBS | Yes | Yes |
| DeMicheli et al. 2011 | Culture | PI | FMBS | Yes | Yes |
| DeSoete et al. 2005 | Culture | No | No | Yes | No |
| Dhaliwal et al. 2017 | Culture | PI | GI | Yes | Yes |
| Dominguez et al. 2010 | Culture | PI | FMBS | Yes | Yes |
| doVale et al. 2016 | qPCR | FMPS | FMBS | Yes | No |
| Eick et al. 2013 | qPCR | FMPS | FMBS | Yes | Yes |
| EuzebioAlves et al. 2013 | Culture | PI | FMBS | Yes | Yes |
| Feres et al. 2009 | Checkerboard | FMPS | FMBS | Yes | Yes |
| Feres et al. 2015 | Checkerboard | FMPS | FMBS | Yes | Yes |
| Fonseca et al. 2015 | qPCR | PI | GI | Yes | Yes |
| Gibson et al. 1994 | Culture | PI | FMBS/ GI | Yes | No |
| Gomez et al. 2011 | Culture | PI | FMBS | Yes | No |
| Grzech-Lesniak et al. 2018 | qPCR | FMPS | FMBS | Yes | Yes |
| Guentsch et al. 2008 | qPCR | FMPS | FMBS | Yes | Yes |
| Haffajee et al. 1997 | Checkerboard | FMPS | FMBS | Yes | Yes |
| Haffajee et al. 1997_2 | Checkerboard | FMPS | FMBS | Yes | Yes |
| Haffajee et al. 2008 | Checkerboard | FMPS | FMBS | Yes | Yes |
| Hagenfeld et al. 2018 | 16S | No | FMBS | Yes | No |
| Han et al. 2012 | qPCR | FMPS | FMBS | Yes | Yes |
| Hayakume et al. 2013 | qPCR | None | FMBS | Yes | Yes |
| Heller et al. 2011 | Checkerboard | FMPS | FMBS | Yes | Yes |
| Ioannou et al. 2009 | Checkerboard | FMPS | GBI | Yes | Yes |
| Ioannou et al. 2011 | Checkerboard | FMPS | GBI | Yes | Yes |
| Isola et al. 2018 | Checkerboard | FMPS | FMBS | Yes | Yes |
| Iziodora et al. 2023 | 16S | No | No | Yes | Yes |
| Jervoe-Storm et al. 2007 | qPCR | FMPS | FMBS | Yes | No |
| Jones et al. 1994 | Checkerboard | PI | FMBS | Yes | No |
| Leonhardt et al. 2007 | Checkerboard | FMPS | FMBS | Yes | No |
| Liu et al. 2013 | qPCR | None | GBI | Yes | Yes |
| Liu et al. 2018 | 16S | PI | BI | Yes | Yes |
| Lombardo et al. 2015 | Culture | VPI | FMBS/ GI | Yes | Yes |
| Lopez et al. 2006 | Checkerboard | FMPS | FMBS | Yes | Yes |
| Luchesi et al. 2013 | qPCR | FMPS | FMBS | Yes | No |
| Lu et al. 2021 | 16S | PI | BI | Yes | Yes |
| Manikandan et al. 2016 | Culture | PI | FMBS/ GI | Yes | No |
| Martande et al. 2016 | Culture | PI | GI | Yes | Yes |
| Martelli et al. 2016 | 16S | No | FMBS | Yes | No |
| Matarazzo et al. 2008 | Checkerboard | FMPS | FMBS | Yes | Yes |
| Matarese et al. 2017 | Checkerboard | FMPS | FMBS | Yes | Yes |
| Mdala et al. 2013 | Checkerboard | FMPS | FMBS | Yes | Yes |
| Mestnik et al. 2010 | Checkerboard | FMPS | FMBS | Yes | Yes |
| Moeintagavi et al. 2007 | Culture | PI | FMBS | Yes | Yes |
| Morales et al. 2018 | Culture | FMPS | FMBS | Yes | Yes |
| Moreira et al. 2015 | Checkerboard | FMPS | FMBS | Yes | Yes |
| Nie et al. 2024 | 16S | No | SBI | Yes | Yes |
| Novaes et al. 2012 | Checkerboard | PI | FMBS | Yes | Yes |
| Park et al. 2018 | qPCR | FMPS | FMBS | Yes | Yes |
| Peralta et al. 2020 | qPCR | PI | GI | Yes | Yes |
| Perrella et al. 2016 | Checkerboard | FMPS | FMBS | Yes | Yes |
| Pradeep et al. 2014 | Culture | PI | FMBS | Yes | Yes |
| Preber et al. 1995 | Culture | PI | GI | Yes | No |
| Pulikkotil et al. 2016 | qPCR | FMPS | FMBS | Yes | Yes |
| Quirynen et al. 1995 | Culture | PI | GI | Yes | Yes |
| Quirynen et al. 1999 | Culture | No | No | Yes | No |
| Quirynen et al. 2000 | Culture | PI | FMBS/ SBI | Yes | Yes |
| Ramiro et al. 2018 | qPCR | None | No | Yes | Yes |
| Renvert et al. 1998 | Culture | FMPS | FMBS | Yes | Yes |
| Roman-Torres et al. 2018 | Culture | PI | GI | Yes | Yes |
| Rooney et al. 2002 | Culture | PI | FMBS | No | No |
| Rosalem et al. 2011 | Checkerboard | No | FMBS | Yes | Yes |
| Saglam et al. 2013 | qPCR | PI | FMBS | Yes | Yes |
| Saglam et al. 2017 | qPCR | PI | FMBS | Yes | Yes |
| Sakellari et al. 2010 | Checkerboard | No | FMBS | Yes | Yes |
| Sampaio et al. 2011 | Checkerboard | FMPS | FMBS | Yes | Yes |
| Sanz-Sanchez et al. 2016 | Culture | PI | FMBS | Yes | Yes |
| Schwarzenberg 2014 | 16S | PPD reported in context with changes to microbiota | | | |
| Sefton et al. 1996 | Culture | PI | BI | Yes | No |
| Shiloa et al. 1997 | Checkerboard | PI | GI | No | No |
| Shiloa et al. 1998 | Checkerboard | PI | GI | No | No |
| Silva et al. 2011 | Checkerboard | FMPS | FMBS | Yes | Yes |
| Silva-Boghossian et al. 2014 | Checkerboard | FMPS | FMBS | Yes | Yes |
| Silva-Senem et al. 2013 | Checkerboard | FMPS | FMBS | No | No |
| Soares et al. 2014 | Checkerboard | FMPS | FMBS | Yes | Yes |
| Soeroso et al. 2017 | qPCR | PI | PBI | Yes | Yes |
| Spooner et al. 2016 | qPCR | None | FMBS | Yes | Yes |
| Sreedhar et al. 2015 | Culture | PI | SBI | Yes | Yes |
| Suchetha et al. 2013 | Culture | PI | GI | No | No |
| Suryaprasanna et al. 2018 | Culture | No | GI | Yes | Yes |
| Swierkot et al. 2009 | qPCR | API | FMBS | Yes | Yes |
| Tabenski et al. 2017 | Checkerboard | API | PBI | No | No |
| Tanaka et al. 2015 | qPCR | FMPS | FMBS | Yes | Yes |
| Tekce et al. 2015 | Culture | PI | FMBS/ GI | Yes | No |
| Teughels et al. 2013 | qPCR | FMPS | FMBS | Yes | Yes |
| Theodoro et al. 2018 | qPCR | None | FMBS | Yes | Yes |
| Timmerman et al. 1996 | Checkerboard | PI | PBI | Yes | Yes |
| Winkel et al. 2001 | Culture | PI | BI | Yes | Yes |
| Xajigeorgiou et al. 2006 | Checkerboard | No | FMBS | Yes | Yes |
| Yilmaz et al. 2012 | Culture | PI | GI | Yes | Yes |
| Yilmaz et al. 2013 | Culture | PI | SBI | Yes | Yes |
| Zengin Celik et al. 2019 | qPCR | PI | FMBS | Yes | Yes |
| FMPS: full mouths plaque scores, PI: plaque index, VPI: visual plaque index, API: approximal plaque index, FMBS: full mouth bleeding on probing, SBI; sulcus bleeding index, GI: gingival index, GBI: gingival bleeding index, PBI: papilla bleeding index, PPD: full mouth probing pocket depth, CAL: full mouth clinical attachment loss | | | | | |

**Figure 23**. Mean clinical parameters ± SD at baseline and 3 months after NSPT in the checkerboard DNA-DNA hybridization technology, real-time quantitative PCR, bacterial culture and 16S rRNA gene sequencing studies. FMBS and FMPS in %, FM-PPD and FM-CAL in mm.


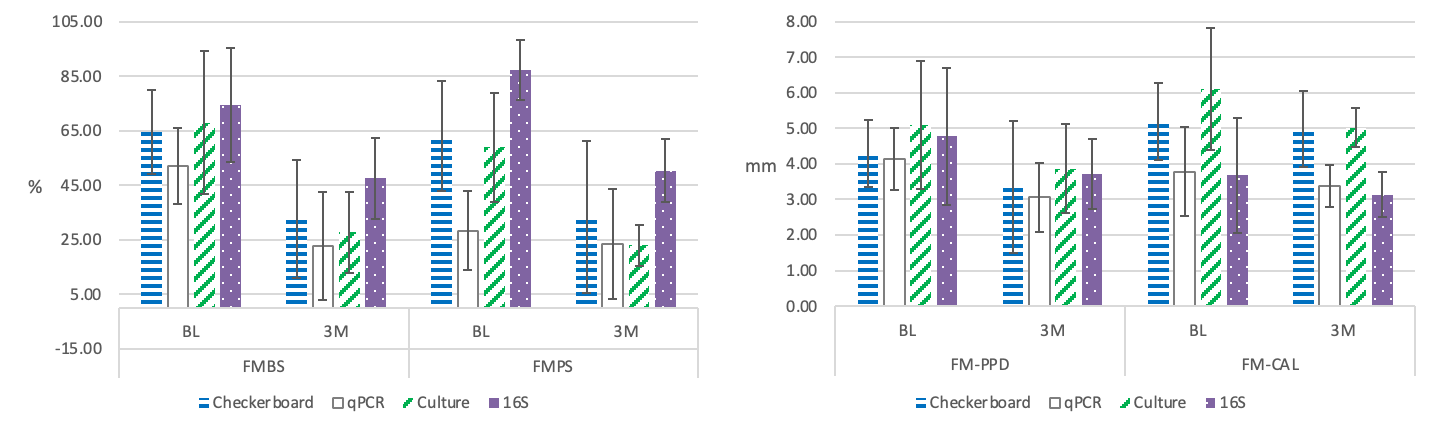


**Appendix S24.** Risk of bias assessment, ROB2 tool for all randomised clinical trials: (A) checkerboard DNA-DNA hybridization technology studies, (B) real-time quantitative PCR studies, (C) bacterial culture studies and (D) 16S rRNA gene sequencing studies according to different domains.

**Appendix S25.** Risk of bias assessment for all other studies, ROBINS-I tool: (A) checkerboard DNA-DNA hybridization technology studies, (B) real-time quantitative PCR studies, (C) bacterial culture studies and (D) 16S rRNA gene sequencing studies according to different domains.

(A)

(B)

(C)

(D)


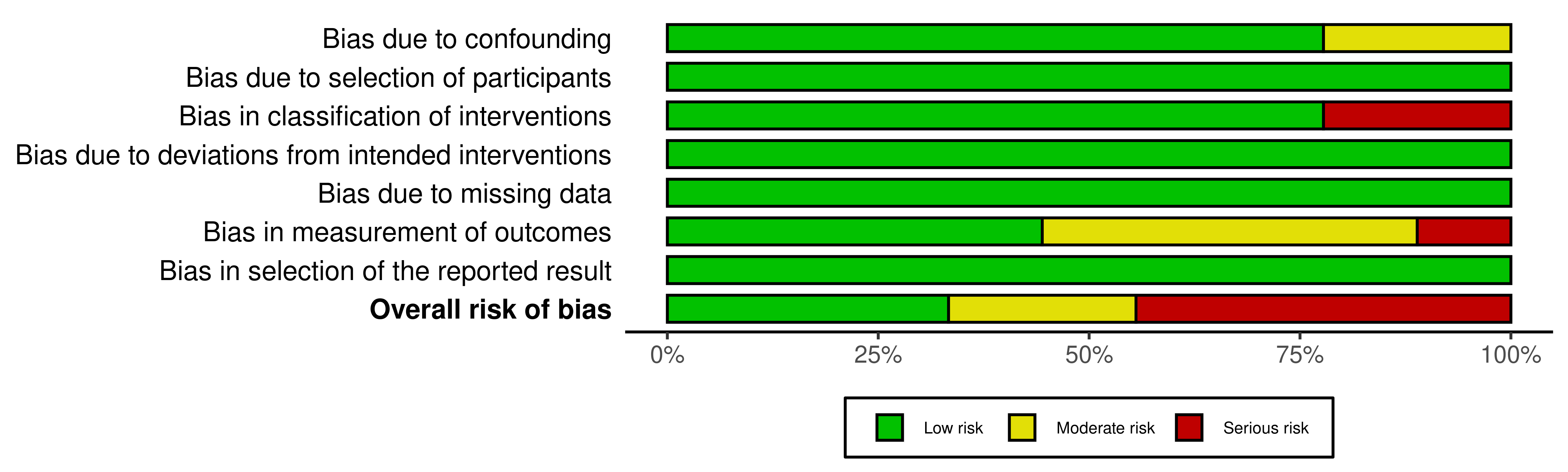

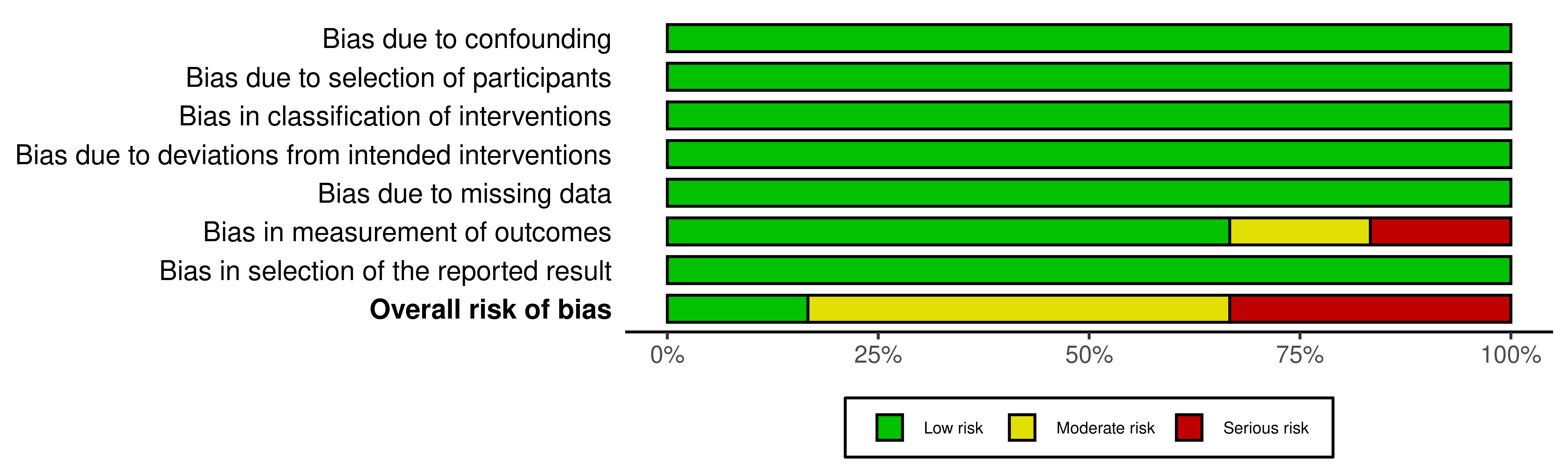

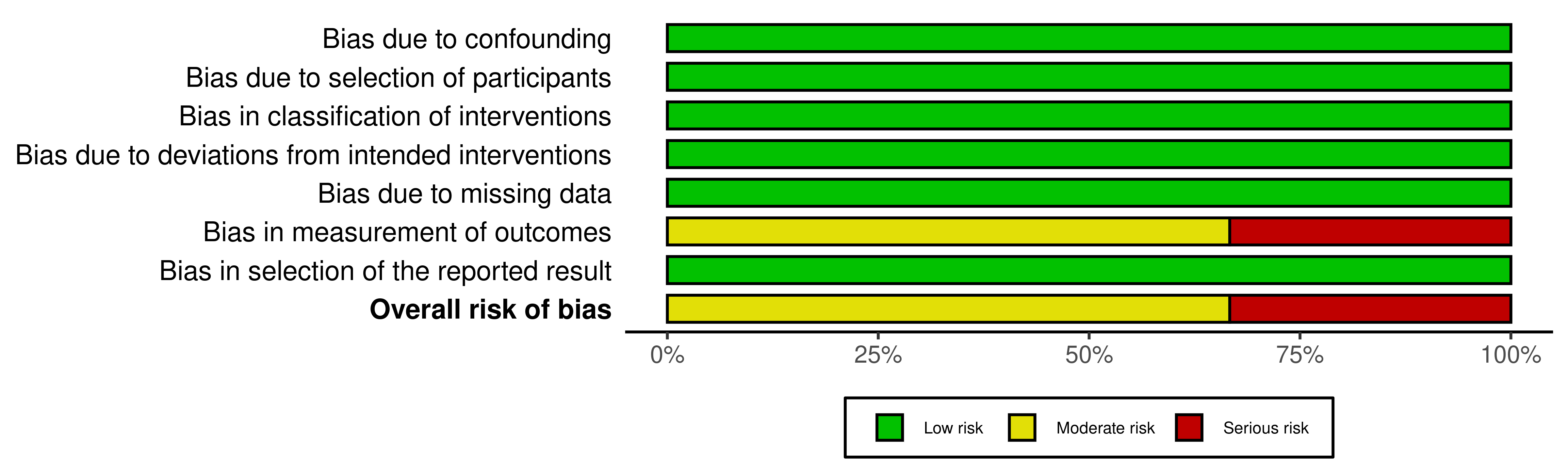

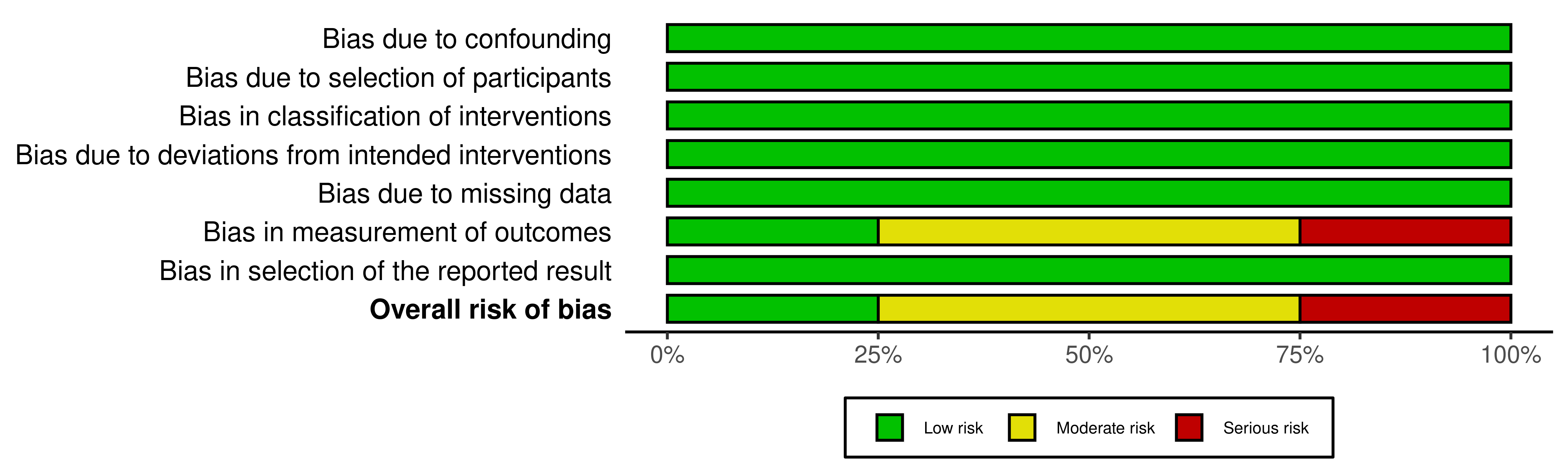


**Appendix S26. Advantage and disadvantages of commonly used hypervariable regions in 16S rRNA gene sequencing**

| Region | bp | Advantages | Disadvantages |
| --- | --- | --- | --- |
| V4 | 252 | - Short lengths enable deeper sequencing of a sample | - High degree of sequence conservation, therefore less able to distinguish between species - Differentiate oral streptococci spp. poorly |
| V3-4 | 427 | - Provide profiles representative of diverse communities at the genus level - Widely used in oral microbiome studies | - High degree of sequence conservation, therefore less able to distinguish between species - Differentiates oral streptococci spp. poorly - Recommended to be used in combination with primers targeting another region on rRNA to allow for species level characterisation of bacterial community |
| V1-2 | 326 | - Greatest nucleotide heterogeneity leading to good discriminatory power between species - Capable of identifying most streptococci at species level | - Less commonly used in oral microbiome studies and therefore less data available for comparisons |
| V5-7 | 426 |  | - High degree of sequence conservation, therefore less able to distinguish between species |
